# Supplementary material for: Nanobody-mediated control of gene expression and epigenetic memory
Source: Nat Commun. 2021 Jan 22;12:537. doi: 10.1038/s41467-020-20757-1 (PMC7822885; doi:10.1038/s41467-020-20757-1)
Supplement: Supplementary file 1 — Supplementary Information [file 41467_2020_20757_MOESM1_ESM.pdf]

Supplementary Information

Nanobody-mediated control of gene expression and epigenetic memory

Mike V. Van[1], Taihei Fujimori[2], Lacramioara Bintu[2†]

1. Department of Biology, Stanford University, Stanford, CA 94305, USA.
2. Department of Bioengineering, Stanford University, Stanford, CA 94305, USA.

†: Corresponding author. Email: lbintu@stanford.edu

Correspondence and requests for materials should be addressed to L.B. (email: lbintu@stanford.edu)

Supplementary Figure 1

Supplementary Figure 2

Supplementary Figure 3

Supplementary Figure 4

Supplementary Figure 5

Supplementary Figure 6

Supplementary Table 1

Supplementary Table 2

Supplementary Table 3

Supplementary Table 4

Supplementary Note

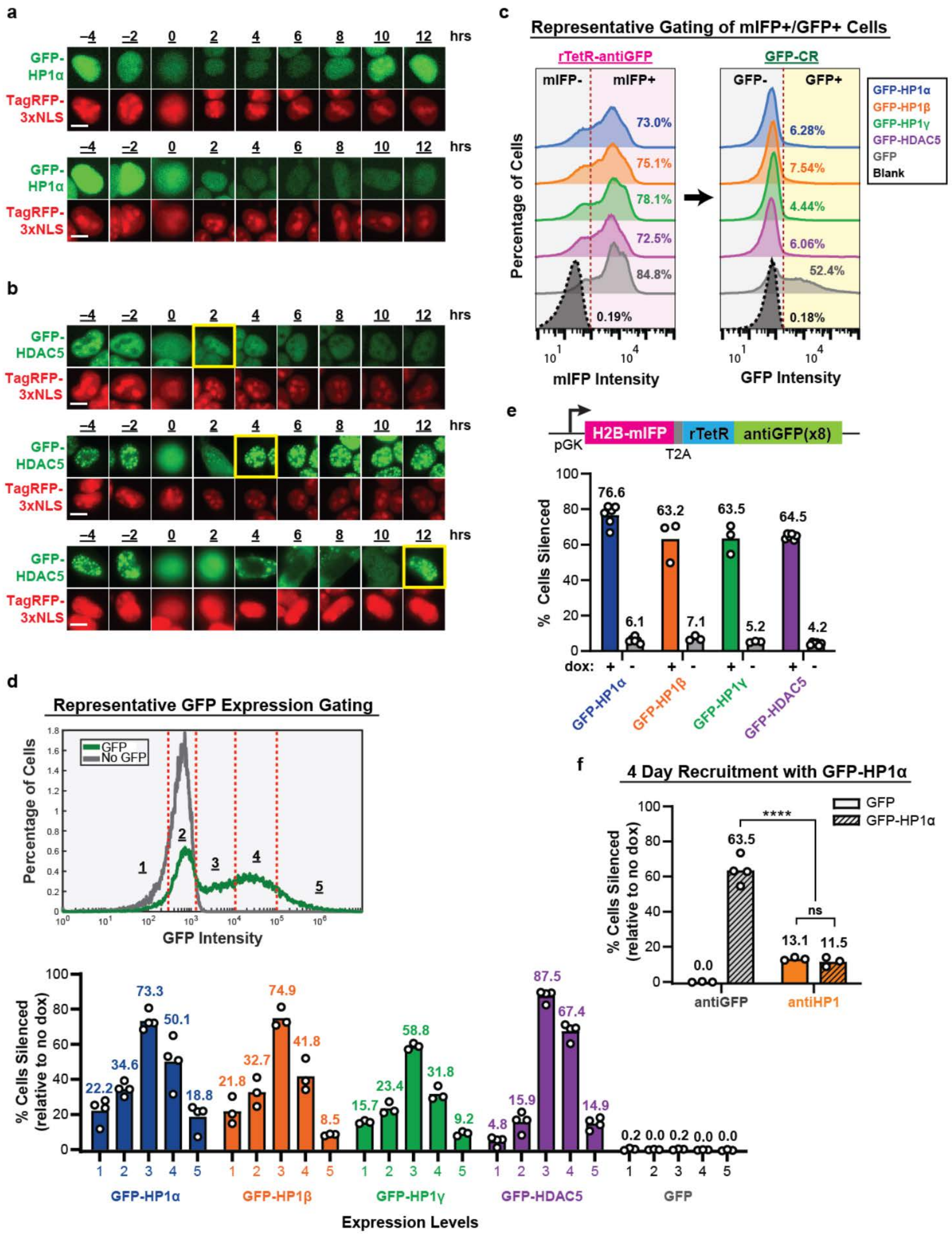
a
GFP-HP1α
TagRFP-3xNLS
−4 −2 0 2 4 6 8 10 12 hrs
b
GFP-HDAC5
TagRFP-3xNLS
c
Representative Gating of mIFP+/GFP+ Cells
rTetR-antiGFP
GFP-CR
mIFP- mIFP+
GFP- GFP+
73.0%
75.1%
78.1%
72.5%
84.8%
0.19%
6.28%
7.54%
4.44%
6.06%
52.4%
0.18%
GFP-HP1α
GFP-HP1β
GFP-HP1γ
GFP-HDAC5
GFP
Blank
Percentage of Cells
mIFP Intensity
GFP Intensity
d
Representative GFP Expression Gating
GFP
No GFP
Percentage of Cells
GFP Intensity
% Cells Silenced (relative to no dox)
Expression Levels
e
pGK
H2B-mIFP
T2A
rTetR
antiGFP(x8)
% Cells Silenced
dox:
76.6
6.1
63.2
7.1
63.5
5.2
64.5
4.2
f
4 Day Recruitment with GFP-HP1α
GFP
GFP-HP1α
% Cells Silenced (relative to no dox)
0.0
63.5
13.1
11.5
****
ns
antiGFP
antiHP1

Supplementary Figure 1: Localization dynamics of GFP-tagged chromatin regulators and recruitment GFP-tagged chromatin regulators

Time-lapse images of cells transiently expressed with **(a)** GFP-tagged HP1α and **(b)** HDAC5. Cells undergoing cell division are represented at time 0 hours. Yellow boxes highlight the re-entry of GFP-tagged chromatin regulators into the nucleus. Time-lapse experiment from 1 biological replicate. White scale bars represent 10 μm. (**c**) Example of mIFP and GFP gating for data in Fig. 1d. This is representative of the gating used for flow cytometry analysis throughout this manuscript. (**d**) Data in Fig. 1d were gated based on different GFP expression levels and analyzed for the percentage of cells with the TagRFP reporter silenced at day 4 of dox. (**e**) Percent cells with reporter silenced after recruitment of an 8x repeat array of antiGFP nanobodies (+dox for 5 days). The experimental set up is the same as in Fig. 1a&d, with the reporter and nanobody array constructs stably integrated, and GFP-CRs transiently transfected. Each dot is an independently transfected biological replicate (GFP-HP1α: n = 6; GFP-HP1β: n = 3; GFP-HP1γ: n = 3; GFP-HDAC5: n = 6). (**f**) Percentage of cells with TagRFP silenced after four days of recruitment with antiGFP (grey) or antiHP1 nanobody (orange). Cells were also co-expressed with GFP or GFP-HP1α (diagonal lines). Means are from 3 replicates; statistical analysis by two-tailed unpaired *t*-test (antiGFP + GFP-HP1α vs. antiHP1 + GFP: ****p = 8.8e-7; antiGFP + GFP-HP1α vs. antiHP1 + GFP-HP1α: ****p = 6.7e-7).

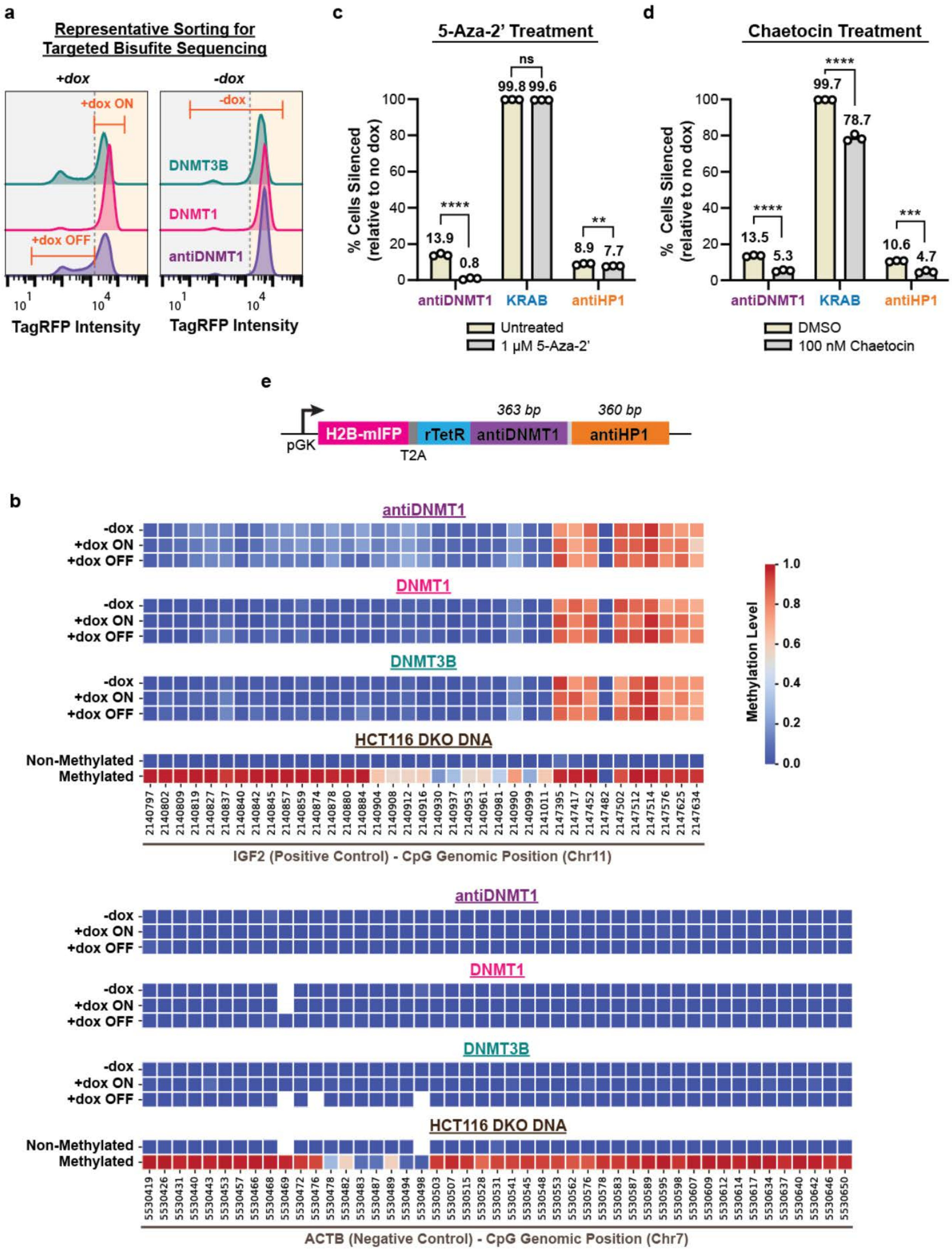
a
Representative Sorting for Targeted Bisufite Sequencing
+dox
-dox
+dox ON
-dox
DNMT3B
DNMT1
+dox OFF
antiDNMT1
TagRFP Intensity
TagRFP Intensity
c
5-Aza-2' Treatment
% Cells Silenced (relative to no dox)
13.9
0.8
99.8
99.6
8.9
7.7
****
ns
**
antiDNMT1
KRAB
antiHP1
Untreated
1 µM 5-Aza-2'
d
Chaetocin Treatment
% Cells Silenced (relative to no dox)
13.5
5.3
99.7
78.7
10.6
4.7
****
****
***
antiDNMT1
KRAB
antiHP1
DMSO
100 nM Chaetocin
e
pGK
H2B-mIFP
T2A
rTetR
363 bp
antiDNMT1
360 bp
antiHP1
b
antiDNMT1
DNMT1
DNMT3B
HCT116 DKO DNA
-dox
+dox ON
+dox OFF
Non-Methylated
Methylated
Methylation Level
1.0
0.8
0.6
0.4
0.2
0.0
2140797 2140802 2140809 2140819 2140827 2140837 2140840 2140842 2140845 2140857 2140859 2140874 2140878 2140880 2140884 2140904 2140908 2140912 2140916 2140930 2140937 2140953 2140961 2140981 2140990 2140999 2141011 2147395 2147417 2147452 2147482 2147502 2147512 2147514 2147576 2147625 2147634
IGF2 (Positive Control) - CpG Genomic Position (Chr11)
5530419 5530426 5530431 5530440 5530443 5530453 5530457 5530466 5530468 5530469 5530472 5530476 5530478 5530482 5530483 5530487 5530489 5530494 5530498 5530503 5530507 5530515 5530528 5530531 5530541 5530545 5530548 5530553 5530562 5530576 5530578 5530583 5530587 5530589 5530595 5530598 5530607 5530609 5530612 5530614 5530617 5530634 5530637 5530640 5530642 5530646 5530650
ACTB (Negative Control) - CpG Genomic Position (Chr7)

Supplementary Figure 2: Targeted bisulfite sequencing controls and treatment of cells with DNA or histone methylation inhibitors

(**a**) Representative sorting for targeted bisulfite sequencing after 5 days of recruitment with antiDNMT1, DNMT1, and DNMT3B (left) and in the absence of dox (right). (**b**) Targeted bisulfite sequencing of two control genes, IGF2 (silent gene with imprinted DNA methylation) and ACTB (active gene, no DNA methylation expected) in the same cell populations as in Fig 2c. Also included are non-methylated DNA controls from the human HCT116 DKO cell line that contains knockouts of DNA methyltransferases DNMT1 and DNMT3B. Methylated DNA was obtained from the non-methylated HCT116 DKO genomic DNA by *in vitro* treatment with the M. SssI methyltransferase. **(c)** Graphs comparing the percentage of cells with TagRFP silenced after four days of recruitment with doxycycline and 5-aza-2'-deoxycytidine (5-Aza-2'), a DNA methyltransferase inhibitor, or **(d)** chaetocin, a broad-spectrum inhibitor of lysine histone methyltransferases. Means are from 3 replicates; statistical analysis by two-tailed unpaired *t*-test (5-Aza-2': antiDNMT1 ****p = 8.0e-14; antiHP1 **p = 0.0046 | Chaetocin: antiDNMT1 ****p = 2.8e-5; KRAB ****p = 3.5e-5, antiHP1 ***p = 0.00019). (**e**) Expression vector for H2B-mIFP and the rTetR-antiDNMT1-antiHP1 fusion under a pGK constitutive promoter with sizes of the DNA encoding for the nanobodies shown in base pairs (bp).

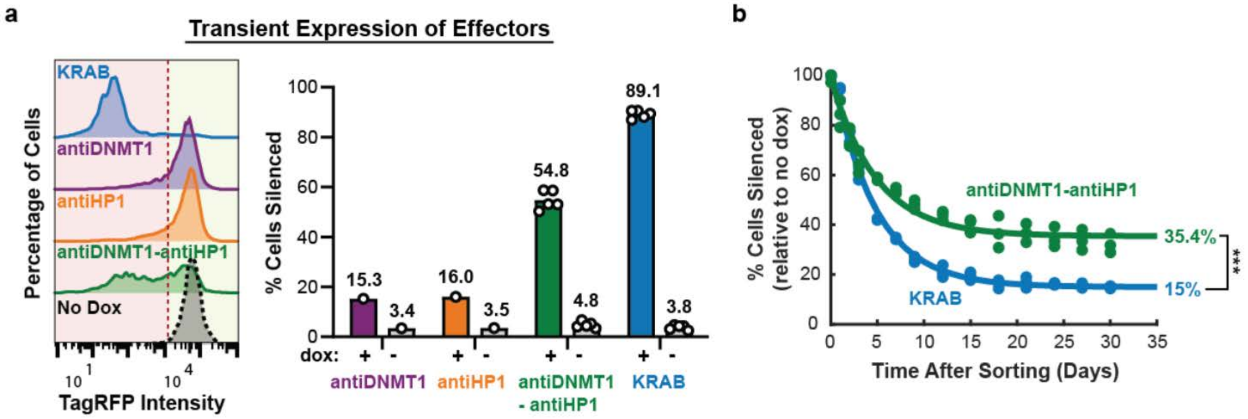

Supplementary Figure 3: Transient expression and recruitment of nanobodies can also silence gene expression and confer memory

(**a**) (left) Reporter fluorescent distributions and percent cells silent (right) after transient expression of rTetR-effector fusions and 5 days of dox treatment. Each dot is an independently transfected biological replicate (antiDNMT1: n = 1; antiHP1: n = 1; antiDNMT1-antiHP1: n = 5; KRAB: n = 5). (**b**) Cells silenced by KRAB and antiDNMT1-antiHP1 in (e) were sorted after 5 days of dox treatment and analyzed by flow cytometry for memory. Each time point contains 3 biological replicates (individual dots). Data were fitted with an exponential decay curve (lines, Methods). Statistical analysis by two-tailed Tukey-test at day 30 after sorting (KRAB vs. antiDNMT1-antiHP1: ***p = 0.00060).

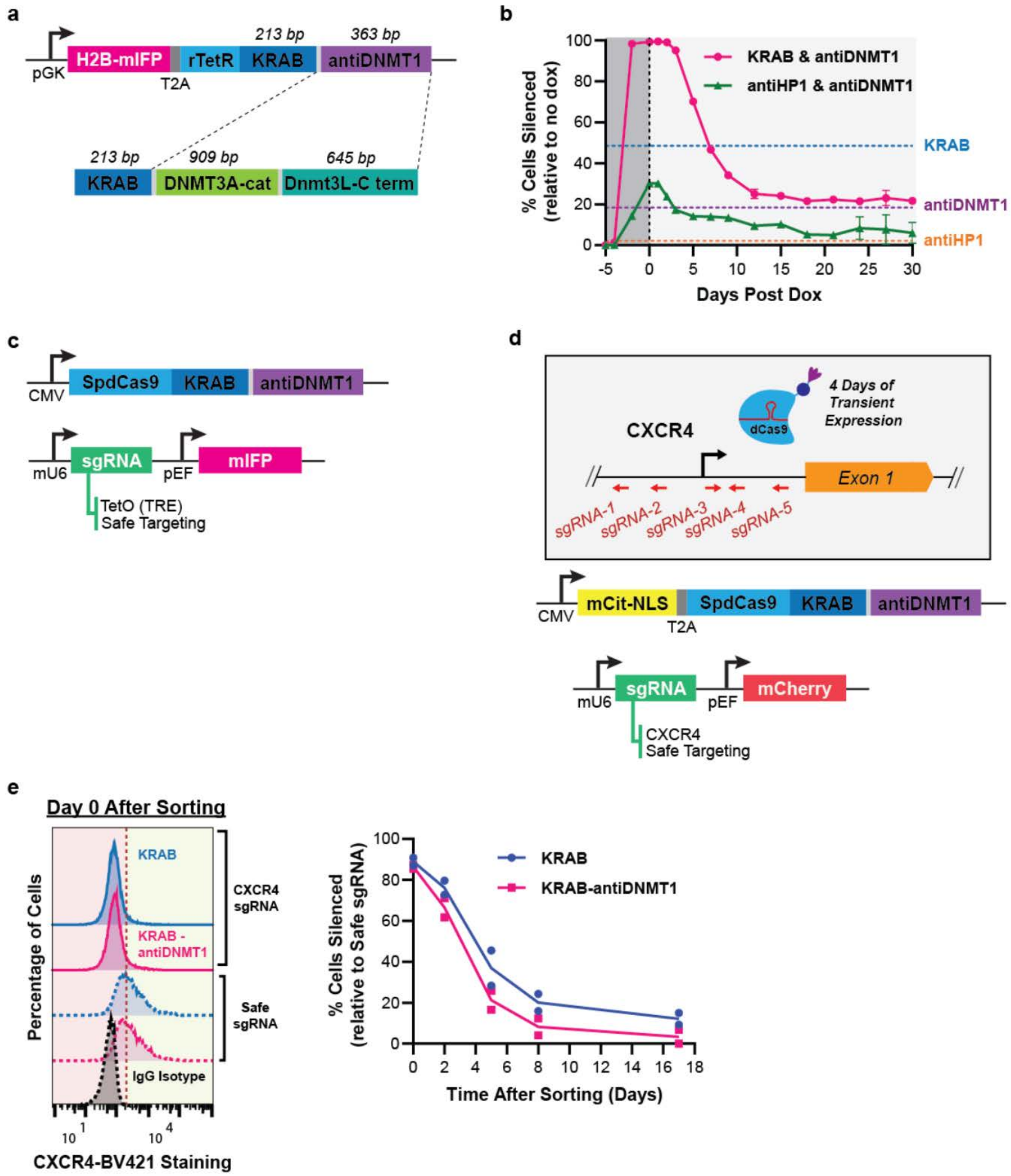

Supplementary Figure 4: Separate co-recruitment of regulators to the reporter gene and *CXCR4* endogenous gene silencing

(**a**) Expression vector for rTetR-KRAB-antiDNMT1 compared to the previously published KRAB-DNMT3A-3L fusion. (**b**) Percent cells with reporter silenced (relative to no dox controls) after co-recruitment of separate fusion of rTetR-effectors at the TagRFP reporter gene. Experimental setup is the same as in Fig. 2a&d. Included for reference are the percentages of cells permanently silenced after individual recruitment with KRAB, antiDNMT1, or antiHP1 (dashed

lines) taken from Fig. 2e. (**c**) Expression vector for dCas9-KRAB-antiDNMT1 under a CMV constitutive promoter. The sgRNA (targeting either the TetO site or a safe genomic site with no annotated function) was expressed on a different vector driven by the mouse U6 promoter and contained a mIFP marker. (**d**) Diagram of sgRNA binding sites for the targeting of dCas9-KRAB-antiDNMT1 to the endogenous *CXCR4* gene. *CXCR4* is a cell surface transmembrane protein, which enables us to use conjugated fluorescent antibodies with flow cytometry to quantify gene expression. We cloned 5 sgRNAs (Table S2) spanning the upstream region of the transcriptional start site of this gene, targeting either the template or non-template strand. The dCas9 and sgRNA constructs were modified to express mCitrine and mCherry, respectively, to allow for cell sorting. (**e**) After transient expression and targeting at the endogenous *CXCR4* gene for 4 days, cells were sorted for the presence of both dCas9 (mCitrine positive) and sgRNA (mCherry positive). Cells were then immunostained for *CXCR4* expression and analyzed by flow cytometry (left). Means of percent cells with silent *CXCR4* from 2 replicates are shown throughout 17 days after sorting (right).

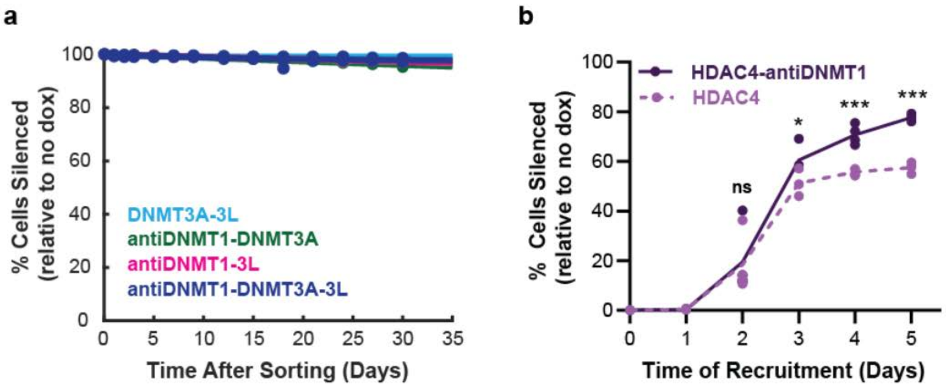

Supplementary Figure 5: Fusion of antiDNMT1 nanobody to DNMT3A-3L and HDAC4

(**a**) After the rTetR-based DNA methyltransferases combinations were transiently transfected and treated with dox for 5 days, silenced cells were sorted, and reactivation was measured by flow cytometry throughout 30 days. Each dot is a biological replicate (DNMT3A-3L: n = 1; antiDNMT1-DNMT3A: n = 1; antiDNMT1-DNMT3L: n = 1; antiDNMT1-DNMT3A-3L: n = 3) and the data are fitted with an exponential decay curve (Methods). (**b**) Transient expression and recruitment of the rTetR-HDAC4-antiDNMT1 fusion to the reporter gene throughout 5 days (n = 4 replicates). Statistical analysis by two-tailed unpaired *t*-test (HDAC4 vs. HDAC4-antiDNMT1: $*p_3 = 0.043$, $***p_4 = 0.00035$, $***p_5 = 0.00024$).

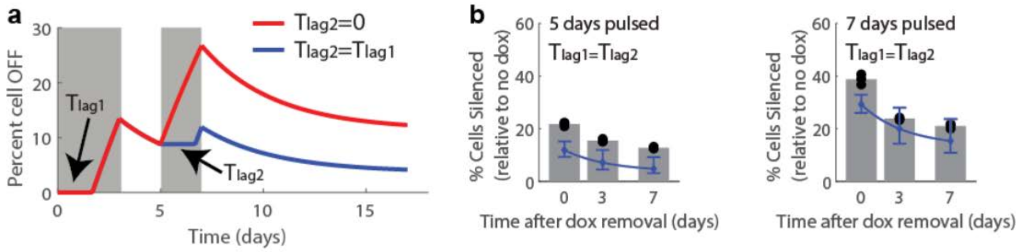

Supplementary Figure 6: Comparison of different models of antiDNMT1-mediated silencing for pulsed recruitment

(a) Predictions of 3-state silencing model for pulsed recruitment with different values of the second lag time between dox addition and start of silencing (T_{lag2}). The predictions of the model shown in red (T_{lag2} = 0) are plotted next to experimental data in Fig. 4f-g. **(b)** Comparison of model predictions and experimental data for the 5 days (left) or 7 day (right) pulsed data for the model where the lag time between dox addition and silencing on the second recruitment pulse is equal to the one on the first pulse. Experimental data from 3 replicates shown as black dots, means as gray bars, and model predictions with 95% CI in blue (Methods).

Supplementary Table 1: List of chromatin regulators used in this study

Included for each CR is the type of chromatin modification it is primarily associated with and a list of repressors known to interact with it (based on the NCBI gene database - https://www.ncbi.nlm.nih.gov/gene).

Name	Type of Modification	Associated Repressors
DNMT1	CpG methylation	DNMT3A, DNMT3B, HP1β, SUV39H1, HDAC1, HDAC2, EZH2
DNMT3A	CpG methylation	DNMT1, DNMT3B, DNMT3L, HP1β, SETDB1, SUV39H1, HDAC1, EZH2
DNMT3B	CpG methylation	DNMT1, DNMT3A, DNMT3L, HP1, SUV39H1, HDAC1, HDAC2, EZH2
DNMT3L	CpG methylation	DNMT3A, DNMT3B, HDAC1
HDAC4	Histone deacetylation	HP1α, HDAC3, HDAC9
HDAC5	Histone deacetylation	HP1α, NCOR1, NCOR2
HP1α	H3K9 Methylation	HP1, SUV39H1, KAP1, HDAC4, HDAC5, HDAC9, DNMT3B
HP1β	H3K9 methylation	HP1, SUV39H1, KAP1, DNMT1, DNMT3A, DNMT3B
HP1γ	H3K9 methylation	HP1, KAP1, DNMT3B
KRAB	H3K9 methylation	HP1, KAP1, SETDB1, CHD3 (NuRD complex)
MeCP2	H3K9 methylation, binds CpG methylated DNA	NCOR1, SIN3A

Supplementary Table 2: List of sgRNA Sequences

Sequences of sgRNAs used in this study are listed below. For endogenous gene targeting of *CXCR4*, sgRNA naming is based on the binding coordinates relative to the transcription start site (TSS), and whether they target the template (T) or non-template (NT) strand. A negative coordinate indicates a binding location upstream of the TSS and a positive coordinate indicates a binding location downstream of the TSS.

Target Gene	sgRNA Sequence	Notes
TetO_sgRNA	GTACGTTCTCTATCACTGATA	Targets the 5x tetracycline operator (TetO) sequence.
Safe_sgRNA-1	GATAGGCACAGGAAATTTGG	Safe guide control used when targeting the reporter gene.
Safe_sgRNA-2	GCACATTTGGATTTCATGTC	Safe guide control used when targeting the *CXCR4* gene.
*CXCR4*_sgRNA-1 (NT, -204)	GAGGCATTTCCTAAGTTTGA	
*CXCR4*_sgRNA-2 (NT, -126)	GCGCGGCTTGGGAAGCCCAG	
*CXCR4*_sgRNA-3 (T, +36)	CAGGTAGCAAAGTGACGCCGA	Previously validated in Yeo et al. 2018.
*CXCR4*_sgRNA-4 (NT, +102)	AACCGCTGGTTCTCCAGATG	
*CXCR4*_sgRNA-5 (NT, +501)	TTCCCCAAGGAAGAGACCGG	

Supplementary Table 3: List of primers used during targeted bisulfite sequencing

Primers used during targeted bisulfite sequencing are listed below. The primers against the pEF promoter were designed specifically to amplify the pEF promoter at the reporter gene and not the endogenous pEF promoter. Primers that overlap CpG sites were designed with a pyrimidine (Y: C or T) at the CpG cytosine in the forward primer, or a purine (R: A or G) in the reverse primer.

Region	Direction	Sequence 5'-3'
5x TetO Site	Fwd	GGGTTAGTAAAGTTTAGGAAGAYGT
	Rev	CTCATACATTCCCACCRTACAC
pEF promoter	Fwd	GTGTAYGGTGGGAATGTATGAG
	Rev	CACACACRACACTTACCTATATTCTAAC
TagRFP-T	Fwd	GGGTTTTATATGGGAGAGAGTTATTATATA
	Rev	TATATAATCTTAAAATTACAAATCAAATAACCCC
ACTB	Fwd	GTYGTTTYGAAAGTTGTTTTTATGGTT
	Rev	CRATTCCCCCCCCATAC
IGF2 #1	Fwd	GTGAGTTGTTGTGGTTTGTGGTTTA
	Rev	CCCACACCTACTCTAACCCCTAA
UGF2 #2	Fwd	TATTGTTTTTAGTTTTTTTTAAATTTGGGTATTGTTTT
	Rev	AACACCRACRAAACAACAACAAATAC

Supplementary Table 4: List of reactivation rates and percentages of cells irreversibly silent after 5 days of recruitment of different regulators to the reporter gene

The percentages of cells silent during the release period τ are fit to an exponential decay: $S(\tau) = (100 - I_1)e^{-k_A \tau} + I_1$, where I_1 is the percentage of cells irreversibly silenced at the end of the recruitment period, and k_A is the rate of reactivation.

Regulator (type of delivery)	Reactivation rate per day (95% confidence interval)	Percentage of cells irreversibly silent (95% confidence interval)	Figure number
rTetR-KRAB (stable)	0.19 (0.17 - 0.21)	48.6 (47.5 - 49.8)	2e
rTetR-antiHP1 (stable)	0.67 (0.53 - 0.81)	2.1 (1.8 - 2.5)	2e
rTetR-antiDNMT1 (stable)	0.61 (0.27 - 0.94)	18.4 (17.7 - 19.2)	2e
rTetR-antiDNMT1-antiHP1 (stable)	0.75 (0.39 - 1.12)	61.4 (60.2 - 62.6)	2e
rTetR-KRAB (transient)	0.22 (0.20 - 0.24)	15.0 (13.5 - 16.5)	S3b
rTetR-antiDNMT1-antiHP1 (transient)	0.21 (0.18 - 0.23)	35.4 (33.3 - 37.5)	S3b
rTetR-KRAB-antiDNMT1 (transient)	0.11 (0.093 - 0.12)	33.5 (30.6 - 36.5)	3b
rTetR-KRAB-antiDNMT1-antiHP1 (transient)	0.11 (0.103 - 0.12)	29.6 (27.9 - 31.3)	3b
dCas9-KRAB (transient)	0.20 (0.17 - 0.23)	18.1 (15.5 - 20.8)	3d
dCas9-KRAB-antiDNMT1 (transient)	0.17 (0.13 - 0.22)	27.2 (23.3 - 31.1)	3d
dCas9-KRAB-MeCP2 (transient)	0.16 (0.14 - 0.18)	17.5 (15.0 - 20.0)	3d
dCas9-KRAB-MeCP2-antiDNMT1 (transient)	0.14 (0.12 - 0.16)	33.0 (30.7 - 35.4)	3d

Supplementary Note: Modeling of Gene Control

Gene silencing and memory upon recruitment of antiDNMT1 at a gene can be described by a kinetic model consisting of 3 gene states (Fig. 4d). The time evolution for the fraction of cells in each of the three states - active (A), reversibly silent (R), and irreversibly silent (I) - can be calculated from the set of differential equations associated with the kinetic rates in Fig. 4d, during recruitment and release, respectively. We denoted the cells in each state with a “+” subscript (i.e. A_+), to indicate when the equations describe the “+dox” silencing period, and with a “-” subscript for the “-dox” period (i.e. A_-). At the end, we combine them into a single function that describes the behavior across the two periods (no subscript, i.e. A).

Derivation of the fractions of cells in each gene state during antiDNMT1 recruitment (+dox)

During recruitment, we denote the time of dox induction as *t*, and can describe the fraction of cells in each state according to the kinetic model in Fig. 4d, top:

$$\frac{dA_+}{dt} = -k_S A_+ \qquad \frac{dR_+}{dt} = k_S A_+ - k_I R_+ \qquad \frac{dI_+}{dt} = k_I R_+$$

We can solve for the fraction of cells active during recruitment directly from the first equation above:

$$A_+(t) = A_0 e^{-k_S t},$$

where A_0 is the fraction of cells active at the beginning of recruitment. Since the total fractions of silent and active cells must add up to 1, the total fraction of silent cells (S) can be described as: $S_+(t) = 1 - A_0 e^{-k_S t}$. This equation is sufficient to describe and fit the silencing phase. Note that in order to express percentages of cell silent as a function of time (as presented in the main figures), we can simply multiply each equation by 100.

To understand how many cells are committed to long term memory (and thus the behavior after CR release), we need to know how silent cells partition into reversibly versus irreversibly silent over time: To solve for $R_+(t)$, we replace $A_+(t) = A_0 e^{-k_S t}$, into $dR_+/dt = k_S A_+ - k_I R_+$:

$$\frac{dR_+}{dt} = k_S A_0 e^{-k_S t} - k_I R_+.$$

We can solve this differential equation to get:

$$R_+(t) = \frac{k_S}{k_I - k_S} A_0 (e^{-k_S t} - e^{-k_I t}) + R_0 e^{-k_I t}$$

The fraction of irreversible silenced cells over time is: $I_+(t) = 1 - A_+(t) - R_+(t)$.

In summary, the function describing the fraction of cells in each of the three states for a given silencing dox signal of duration t (and no reactivation period):

$$A_+(t) = A_0 e^{-k_S t}$$

$$R_+(t) = \frac{k_S}{k_I - k_S} A_0 (e^{-k_S t} - e^{-k_I t}) + R_0\, e^{-k_I t} \qquad (1)$$

$$\mathrm{I}_+(t) = 1 - A_+(t) - \mathrm{R}_+(t) = 1 - \frac{k_I}{k_I - k_S} A_0 e^{-k_S t} + \left(\frac{k_S}{k_I - k_S} A_0 - R_0\right) e^{-k_I t}$$

Derivation of the fractions of cells in each gene state during antiDNMT1 release (-dox)

During release (-dox), we denote the time since dox was removed as τ, and can describe the fraction of cells in each state according to the kinetic model in Fig. 4d, bottom:

$$\frac{dA_-}{d\tau} = k_A R_- \qquad \frac{dR_-}{d\tau} = -k_A R_- \qquad \frac{dI_-}{d\tau} = 0$$

We can solve for the fraction of cells reversibly silent during release from the dR_-/dt equation above:

$$R_-(\tau) = R_1 e^{-k_A \tau},$$

where $R_1$is the fraction of reversibly silenced cells at the beginning of release (end of recruitment).

During release, the irreversible fraction stays constant, at the value reached at the end of recruitment, I_1: $I_-(\tau) = I_1$

The total fraction of cells silent over time in the release phase, $S_-(\tau) = R_-(\tau) + I_-(\tau)$, is described by an exponential decay to the irreversible fraction:

$$S_-(\tau) = R_1 e^{-k_A \tau} + I_1.$$

The fraction of cells active is: $A_-(\tau) = 1 - I_-(\tau) - R_-(\tau)$.

In summary:

$$A_-(\tau) = 1 - I_1 - R_1 e^{-k_A \tau}$$
$$R_-(\tau) = R_1 e^{-k_A \tau} \qquad (2)$$
$$I_-(\tau) = I_1$$

General solution and additional considerations for fitting the data

We can combine the results of the two previous sections to obtain general solutions that describe the cells in each state across both recruitment and release times. The starting fractions of cells R_1, I_1, at the beginning of the –dox release, depend on the duration of +dox period t: $R_1 = R_+(t)$, and $I_1 = I_+(t)$. Therefore, based on the solutions derived in the release section above (equations (2)):

$$A(t,\tau) = 1 - I_+(t) - R_+(t) e^{-k_A \tau}$$
$$R(t,\tau) = R_+(t)\, e^{-k_A \tau}$$
$$I(\mathrm{t},\mathrm{\tau}) = \mathrm{I}_+(\mathrm{t})$$

By replacing $A_+(t)$, $R_+(t)$, and $I_+(t)$ with the set of equations (1) derived in the recruitment section, the general solution after a recruitment signal (+dox) of duration *t*, and reactivation period (-dox) of duration τ becomes:

$$A(t,\tau) = \frac{k_I}{k_I - k_S} A_0 e^{-k_S t} - (\frac{k_S}{k_I - k_S} A_0 - R_0)\, e^{-k_I t} - \left(\frac{k_S}{k_I - k_S} A_0 (e^{-k_S t} - e^{-k_I t}) + R_0\, e^{-k_I t}\right) e^{-k_A \tau}$$
$$R(t,\tau) = \left(\frac{k_S}{k_I - k_S} A_0 (e^{-k_S t} - e^{-k_I t}) + R_0\, e^{-k_I t}\right) e^{-k_A \tau} \qquad (3)$$
$$I(\mathrm{t},\mathrm{\tau}) = 1 - \frac{k_I}{k_I - k_S} A_0 e^{-k_S t} + (\frac{k_S}{k_I - k_S} A_0 - R_0)\, e^{-k_I t}$$

During recruitment, we observe a time lag between dox addition and the onset of silencing (T_{lag}). Therefore, for fitting purposes, in the equations above t becomes $t - T_{lag}$, for recruitment times larger than T_{lag}, and no changes in the fractions of silent/active cells are allowed at shorter times. The final equations used to calculate the fraction of cells silent over time during a period of +dox induction are:

$$A_+(t) = \begin{cases} A_0 & \text{, for } t < T_{lag} \\ A_0 e^{-k_S(t - T_{lag})} & \text{, for } t \geq T_{lag} \end{cases}$$

$$R_+(t) = \begin{cases} R_0 & \text{, for } t < T_{lag} \\ \frac{k_S}{k_I - k_S} A_0 (e^{-k_S(t-T_{lag})} - e^{-k_I(t-T_{lag})}) + R_0\, e^{-k_I(t-T_{lag})} & \text{, for } t \geq T_{lag} \end{cases}$$

$$\mathrm{I}_+(\mathrm{t}) = \begin{cases} 1 - A_0 - R_0 & \text{, for } t < T_{lag} \\ 1 - \frac{k_I}{k_I - k_S} A_0 e^{-k_S(t-T_{lag})} + (\frac{k_S}{k_I - k_S} A_0 - R_0)\, e^{-k_I(t-T_{lag})} & \text{, for } t \geq T_{lag} \end{cases}$$

For fitting a continuous dox signal and the reactivation following it, we start with all cells silenced: $A_0 = 1, R_0 = 0, I_0 = 0$. Therefore, the equations for silencing simplify to:

$$A_+(t) = \begin{cases} 1 & \text{, for } t < T_{lag} \\ e^{-k_S(t - T_{lag})} & \text{, for } t \geq T_{lag} \end{cases}$$

$$R_+(t) = \begin{cases} 0 & \text{, for } t < T_{lag} \\ \frac{k_S}{k_I - k_S} (e^{-k_S(t-T_{lag})} - e^{-k_I(t-T_{lag})}) & \text{, for } t \geq T_{lag} \end{cases}$$

$$\mathrm{I}_+(\mathrm{t}) = \begin{cases} 0 & \text{, for } t < T_{lag} \\ 1 - \frac{k_I}{k_I - k_S} e^{-k_S(t-T_{lag})} + \frac{k_S}{k_I - k_S}\, e^{-k_I(t-T_{lag})} & \text{, for } t \geq T_{lag} \end{cases}$$

The general solution in the case when we start with all cells active ($A_0 = 1, R_0 = 0, I_0 = 0$) becomes:

$$A(t,\tau) = \frac{k_I}{k_I - k_S} e^{-k_S(t-T_{lag})} - \frac{k_S}{k_I - k_S}\, e^{-k_I(t-T_{lag})} - \frac{k_S}{k_I - k_S} (e^{-k_S(t-T_{lag})} - e^{-k_I(t-T_{lag})}) e^{-k_A \tau},$$

$$R(t,\tau) = \frac{k_S}{k_I - k_S} (e^{-k_S(t-T_{lag})} - e^{-k_I(t-T_{lag})})\, e^{-k_A \tau}\ , \tag{4}$$

$$I(t,\tau) = 1 - \frac{k_I}{k_I - k_S} e^{-k_S(t-T_{lag})} + \frac{k_S}{k_I - k_S}\, e^{-k_I(t-T_{lag})}\ ,$$

for all recruitment times $t \geq T_{lag}$, and $A(t,\tau) = 1, R(t,\tau) = 0\ , I(t,\tau) = 0$ for $t < T_{lag}$.
