# Supplementary material for: Nanobody-mediated control of gene expression and epigenetic memory
Source: Nat Commun. 2021 Jan 22;12:537. doi: 10.1038/s41467-020-20757-1 (PMC7822885; doi:10.1038/s41467-020-20757-1)
Supplement: Supplementary file 2 — Reporting Summary [file 41467_2020_20757_MOESM2_ESM.pdf]

nature research

Corresponding author(s): Lacramioara Bintu (NCOMMS-20-32169A)

Last updated by author(s): Dec 14, 2020

Reporting Summary

Nature Research wishes to improve the reproducibility of the work that we publish. This form provides structure for consistency and transparency in reporting. For further information on Nature Research policies, see our Editorial Policies and the Editorial Policy Checklist.

Statistics

For all statistical analyses, confirm that the following items are present in the figure legend, table legend, main text, or Methods section.

n/a	Confirmed	
☐	☒	The exact sample size (*n*) for each experimental group/condition, given as a discrete number and unit of measurement
☐	☒	A statement on whether measurements were taken from distinct samples or whether the same sample was measured repeatedly
☐	☒	The statistical test(s) used AND whether they are one- or two-sided<br>*Only common tests should be described solely by name; describe more complex techniques in the Methods section.*
☒	☐	A description of all covariates tested
☐	☒	A description of any assumptions or corrections, such as tests of normality and adjustment for multiple comparisons
☐	☒	A full description of the statistical parameters including central tendency (e.g. means) or other basic estimates (e.g. regression coefficient) AND variation (e.g. standard deviation) or associated estimates of uncertainty (e.g. confidence intervals)
☐	☒	For null hypothesis testing, the test statistic (e.g. *F*, *t*, *r*) with confidence intervals, effect sizes, degrees of freedom and *P* value noted<br>*Give P values as exact values whenever suitable.*
☒	☐	For Bayesian analysis, information on the choice of priors and Markov chain Monte Carlo settings
☒	☐	For hierarchical and complex designs, identification of the appropriate level for tests and full reporting of outcomes
☐	☒	Estimates of effect sizes (e.g. Cohen's *d*, Pearson's *r*), indicating how they were calculated

Our web collection on statistics for biologists contains articles on many of the points above.

Software and code

Policy information about availability of computer code

Data collection	Time-lapse images were acquired using the LAS X software (ver 3.7.4; Leica Microsystems). Flow cytometry data were collected using CytExpert Software (ver 2.4; Beckman Coulter).
Data analysis	A custom MATLAB (R2016a) program called EasyFlow v0.3.17 (https://antebilab.github.io/easyflow/) was used to analyze data generated by flow cytometry. Time-lapse movies were analyzed using ImageJ (ver 1.52p) and plots of cell lineage were plotted using MATLAB (R2016a). Exponential decay curves for memory/reactivation data and the model for gene control was derived using MATLAB (R2016a). Statistical significance was evaluated using GraphPad Prism 8. Targeted bisulfite sequencing reads were identified using Illumina base-calling software (ver 2.6) and then was analyzed in Python (ver 3.9).

For manuscripts utilizing custom algorithms or software that are central to the research but not yet described in published literature, software must be made available to editors and reviewers. We strongly encourage code deposition in a community repository (e.g. GitHub). See the Nature Research guidelines for submitting code & software for further information.

Data

Policy information about availability of data

All manuscripts must include a data availability statement. This statement should provide the following information, where applicable:
- Accession codes, unique identifiers, or web links for publicly available datasets
- A list of figures that have associated raw data
- A description of any restrictions on data availability

All data generated during this study are included in this manuscript and available from the corresponding author upon request. Data showing protein interactions listed in Supplementary Table 1 are available from the NCBI gene database. Targeted bisulfite sequencing data are available from the NCBI SRA database (Accession #: PRJNA684608).

Field-specific reporting

Please select the one below that is the best fit for your research. If you are not sure, read the appropriate sections before making your selection.

- [x] Life sciences
- [ ] Behavioural & social sciences
- [ ] Ecological, evolutionary & environmental sciences

For a reference copy of the document with all sections, see nature.com/documents/nr-reporting-summary-flat.pdf

Life sciences study design

All studies must disclose on these points even when the disclosure is negative.

Sample size	No sample size estimates were performed. The sample sizes used in this study are consistent with those used by similar genome editing and gene regulation studies such as: Gao Y, et al. 2016. Nature Methods. 13:1043-1049; Yeo N, et al. 2018. Nature Methods. 15:611-616.
Data exclusions	No samples/data were excluded from analysis.
Replication	All experiments were completed in replicates (at least 2 times) to verify the reproducibility of the findings, except for experiments with transient expression and recruitment of antiHP1 or antiDNMT1 to the reporter gene (Fig. S3a). This is because we believe replicates for these two nanobodies (when stably integrated) in Fig. 2b were sufficient. All attempts at replication were successful.
Randomization	No randomization was performed. This was not relevant to the study because experimental samples were derived from the same cell line.
Blinding	No blinding was performed. Blinding was not relevant to this study. Identification of samples was key to preventing mix up of experimental samples during stable cell line generation, transfections of effectors, doxycycline treatment, and cell sorting.

Reporting for specific materials, systems and methods

We require information from authors about some types of materials, experimental systems and methods used in many studies. Here, indicate whether each material, system or method listed is relevant to your study. If you are not sure if a list item applies to your research, read the appropriate section before selecting a response.

Materials & experimental systems

n/a	Involved in the study
[ ]	[x] Antibodies
[ ]	[x] Eukaryotic cell lines
[x]	[ ] Palaeontology and archaeology
[x]	[ ] Animals and other organisms
[x]	[ ] Human research participants
[x]	[ ] Clinical data
[x]	[ ] Dual use research of concern

Methods

n/a	Involved in the study
[x]	[ ] ChIP-seq
[ ]	[x] Flow cytometry
[x]	[ ] MRI-based neuroimaging

Antibodies

Antibodies used	Monoclonal Brilliant Violet 421 (BV421) labeled anti-CXCR4 antibody (clone 12G5 (1:20); BioLegend #306218). BV421 labeled IgG2a Isotype Control (clone MOPC-173 (1:20); Biolegend #400260). antiDNMT1 and antiHP1 nanobody plasmids were obtained from ChromoTek (cat# dcg) and Institut Curie (Moutel S, et al. 2016. eLife. 5:e16228), respectively.
Validation	Both antibodies were tested and validated by immunofluorescent staining with flow cytometric analysis in human peripheral blood lymphocytes by manufacturer (validation statement and flow cytometry histogram can be found on the manufacturer's website). The same antibodies (same clone) but conjugated with a different fluorophore have also been tested in HEK293T cells by flow cytometry (Gao Y, et al. 2016. Nature Methods. 13:1043-1049). We further validated the antibodies by targeting the CXCR4 gene in HEK293T cells with dCas9-KRAB (using a previously validated sgRNA against CXCR4) to confirm the reduction in CXCR4 immunofluorescence with flow cytometry (data in manuscript).

Eukaryotic cell lines

Policy information about cell lines

Cell line source(s)	HEK293T cell line sourced from Takara Bio (cat # 632180).
Authentication	None of the cell lines were authenticated.
Mycoplasma contamination	Cell lines were not tested for mycoplasma contamination.

Commonly misidentified lines (See ICLAC register)	No commonly misidentified lines were used.

Flow Cytometry

Plots

Confirm that:

- [x] The axis labels state the marker and fluorochrome used (e.g. CD4-FITC).
- [x] The axis scales are clearly visible. Include numbers along axes only for bottom left plot of group (a 'group' is an analysis of identical markers).
- [x] All plots are contour plots with outliers or pseudocolor plots.
- [x] A numerical value for number of cells or percentage (with statistics) is provided.

Methodology

Sample preparation	HEK293T cells (derived from human embryonic kidney cells) were cultured at 37°C under a humidified atmosphere with 5% CO_2 and maintained in Dulbecco's modified Eagle medium supplemented with 25mM D-glucose, 1 mM sodium pyruvate, 1x GlutaMAXTM, and 10% Tet Approved FBS. For flow cytometry analysis, cells were harvested using 0.25% Trypsin. A fraction of the cells (varying between one half to one twentieth, depending on cell density) were re-plated for the next time point. The remaining cells were resuspended in flow buffer (1x Hank's Balanced Salt Solution and 2.5 mg/mL BSA) and filtered through a 40 µm strainer, to remove cell clumps, for flow cytometry analysis. For experiments analyzing the expression of the endogenous CXCR4 gene, cells were first trypsinized with 0.25% Trypsin and then washed with 1% BSA in 1x DPBS. Cells were then incubated on ice for 1 hour with monoclonal Brilliant Violet 421 (BV421) labeled anti-CXCR4 antibody (1:20). BV421 labeled IgG2a (1:20) served as a isotype control. Afterwards, cells were washed three times with 1% BSA/DPBS and then analyzed by flow cytometry.
Instrument	CytoFLEX S Flow Cytometer (Beckman Coulter #C09766)
Software	Flow cytometry data were analyzed with a custom MATLAB program called EasyFlow (https://antebilab.github.io/easyflow/).
Cell population abundance	For experiments investigating memory dynamics of effector fusions at the reporter gene, a minimum of 300,000 silenced (TagRFP negative) cells for each sample were sorted from a starting population of about 3.0 x 10^6 cells. For memory dynamics of endogenous CXCR4 gene targeting, a minimum of 200,000 cells double positive for dCas9 (mCitrine) and sgRNA (mCherry) were sorted from a starting population of about 3.0 x 10^6 cells. Before and after sorting, cells were analyzed by flow cytometry and fluorescent microscopy to confirm purity of sorted samples.
Gating strategy	HEK293T cells were first gated based on their forward and side scatter to separate the cell population from cellular debris and dead cells. Cell doublets were further excluded by gating for single cells based on their forward scatter area and height. For silencing dynamics, cells expressing the effector fusions and/or sgRNA were gated for their respective marker/ fluorochrome relative to the cells with no effectors/sgRNA. A manual gate was imposed on the TagRFP fluorescence to determine the percentage of silent cells for each sample. The gate was selected to contain 1-5% of the positive TagRFP signal in untreated cells. For experiments analyzing the expression of the endogenous CXCR4 gene, gating was determined based on the BV421 fluorophore relative to the IgG isotype control. A total minimum of 20,000 events were recorded for each sample.

- [x] Tick this box to confirm that a figure exemplifying the gating strategy is provided in the Supplementary Information.
