# Supplementary figures and images for: Nanobody-mediated control of gene expression and epigenetic memory (part 2 of 2)
Source: Nat Commun. 2021 Jan 22;12:537. doi: 10.1038/s41467-020-20757-1 (PMC7822885; doi:10.1038/s41467-020-20757-1)

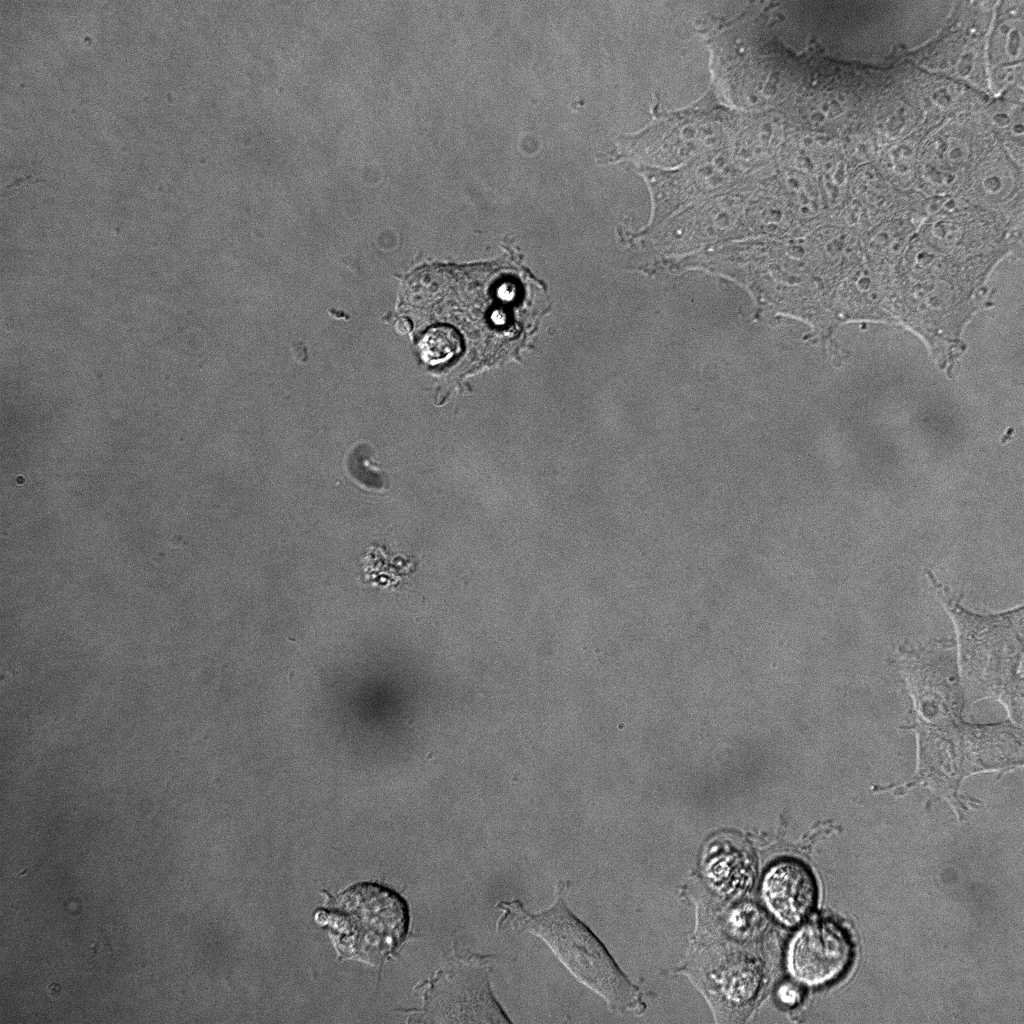

Supplement: Supplementary file 3 — Source Data [file 41467_2020_20757_MOESM3_ESM.zip › source_data/figure 1b-c images/HDAC5_woDox_images/Position010_t154_ch00.tif]

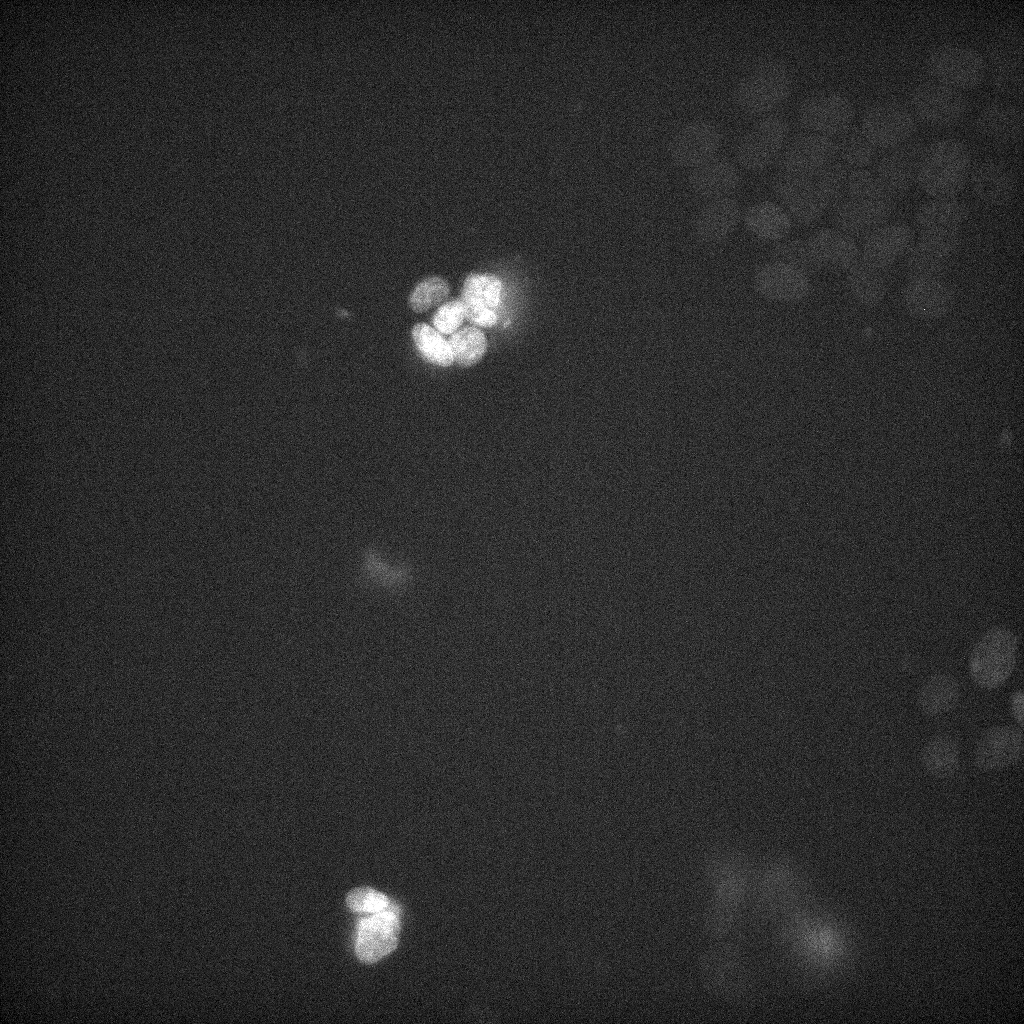

Supplement: Supplementary file 3 — Source Data [file 41467_2020_20757_MOESM3_ESM.zip › source_data/figure 1b-c images/HDAC5_woDox_images/Position010_t154_ch01.tif]

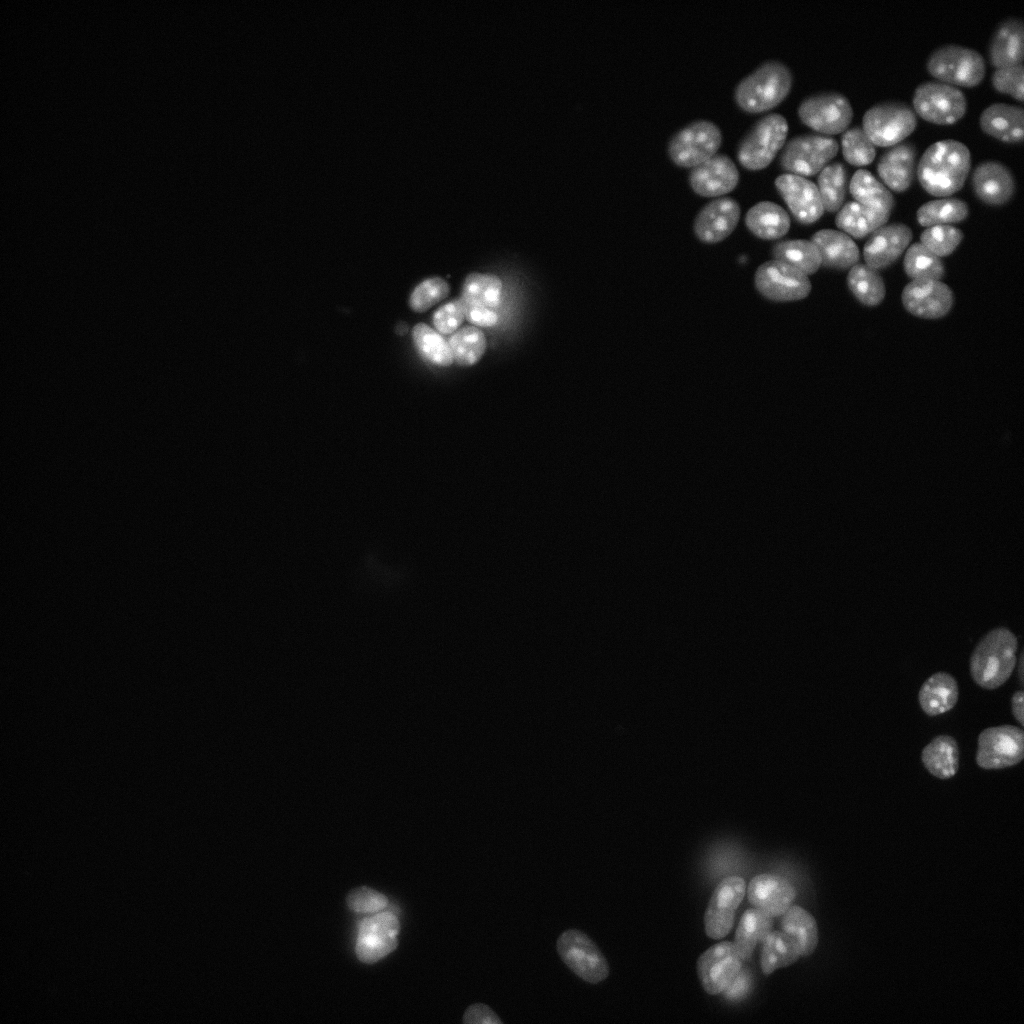

Supplement: Supplementary file 3 — Source Data [file 41467_2020_20757_MOESM3_ESM.zip › source_data/figure 1b-c images/HDAC5_woDox_images/Position010_t154_ch02.tif]

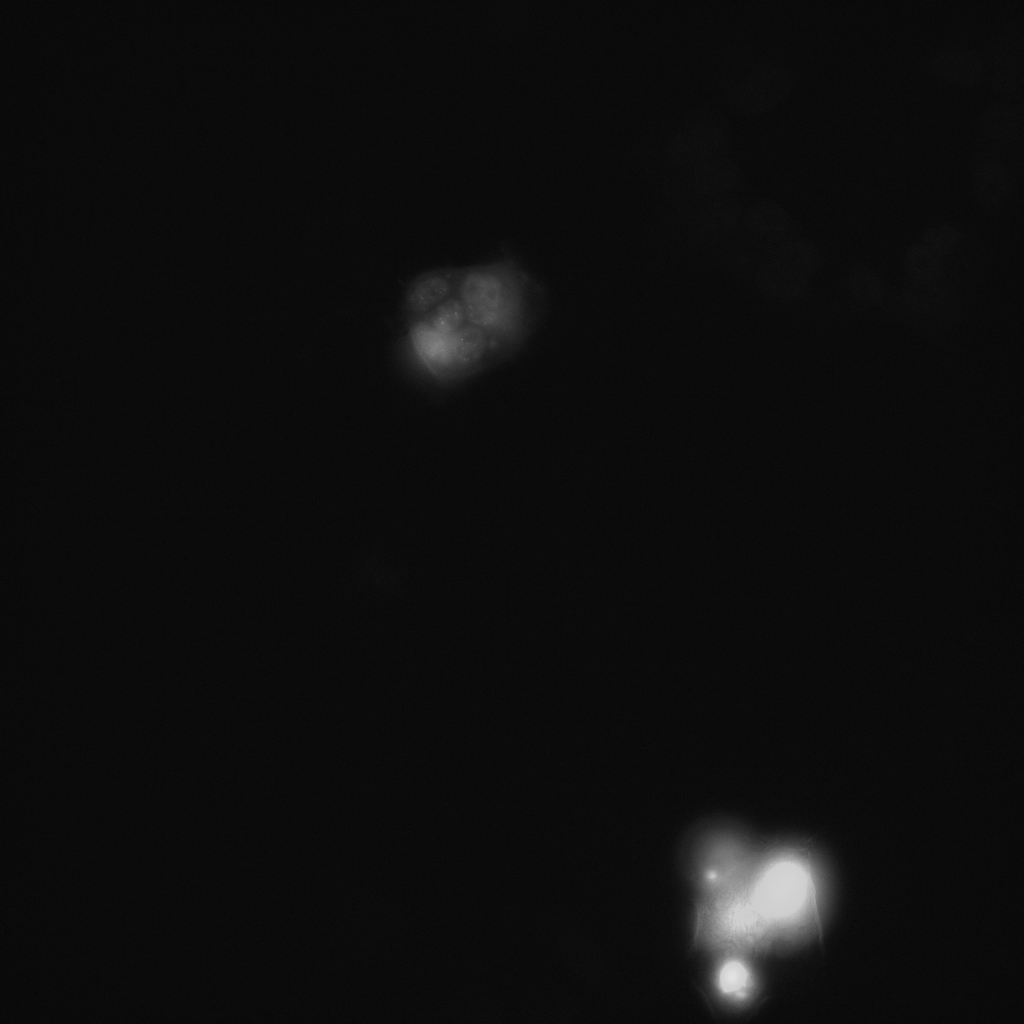

Supplement: Supplementary file 3 — Source Data [file 41467_2020_20757_MOESM3_ESM.zip › source_data/figure 1b-c images/HDAC5_woDox_images/Position010_t154_ch03.tif]

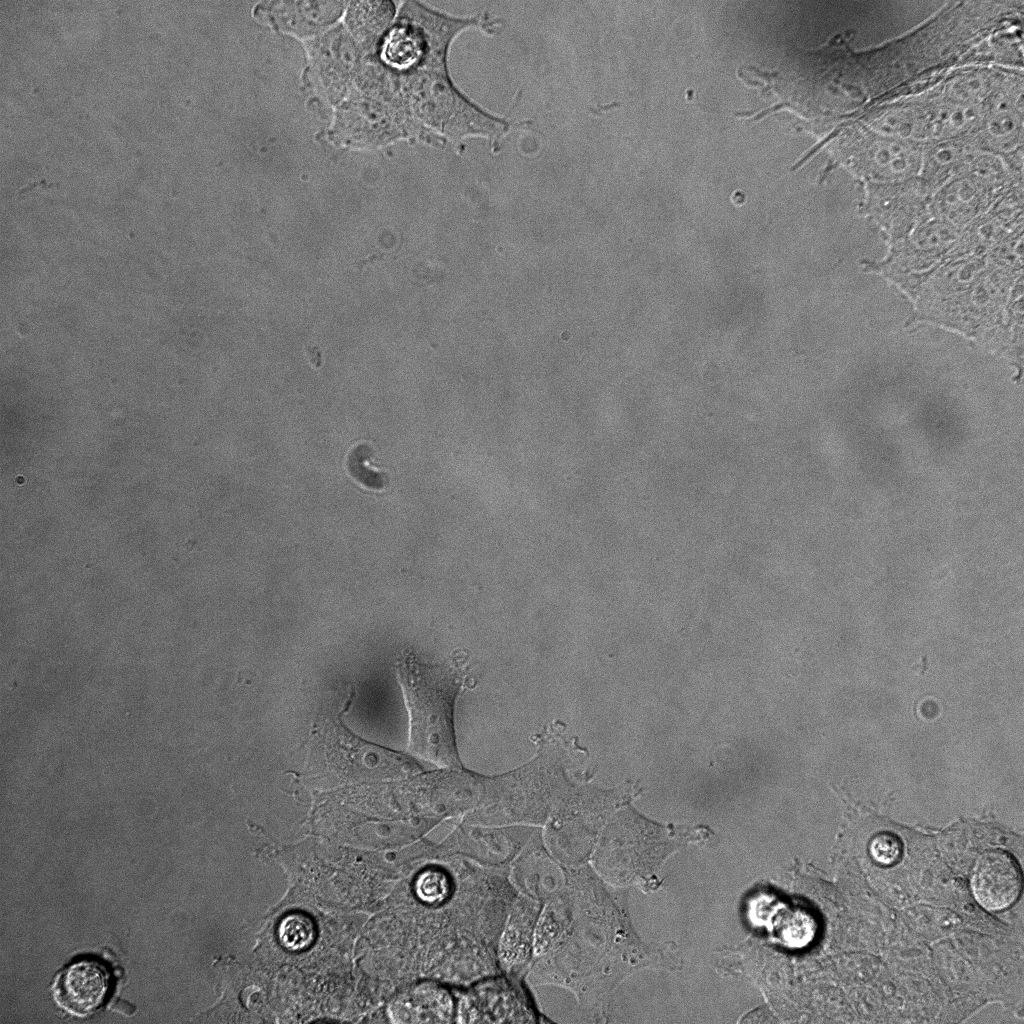

Supplement: Supplementary file 3 — Source Data [file 41467_2020_20757_MOESM3_ESM.zip › source_data/figure 1b-c images/HDAC5_woDox_images/Position010_t200_ch00.tif]

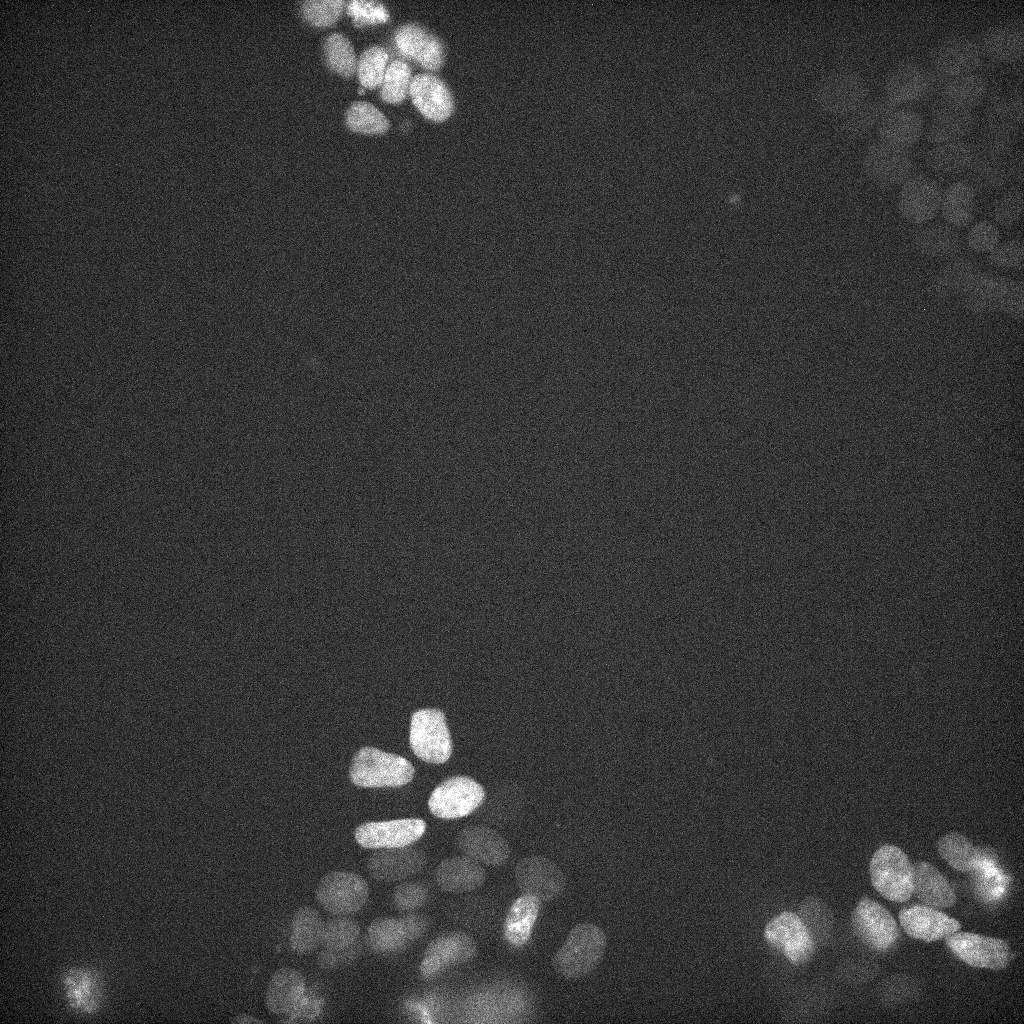

Supplement: Supplementary file 3 — Source Data [file 41467_2020_20757_MOESM3_ESM.zip › source_data/figure 1b-c images/HDAC5_woDox_images/Position010_t200_ch01.tif]

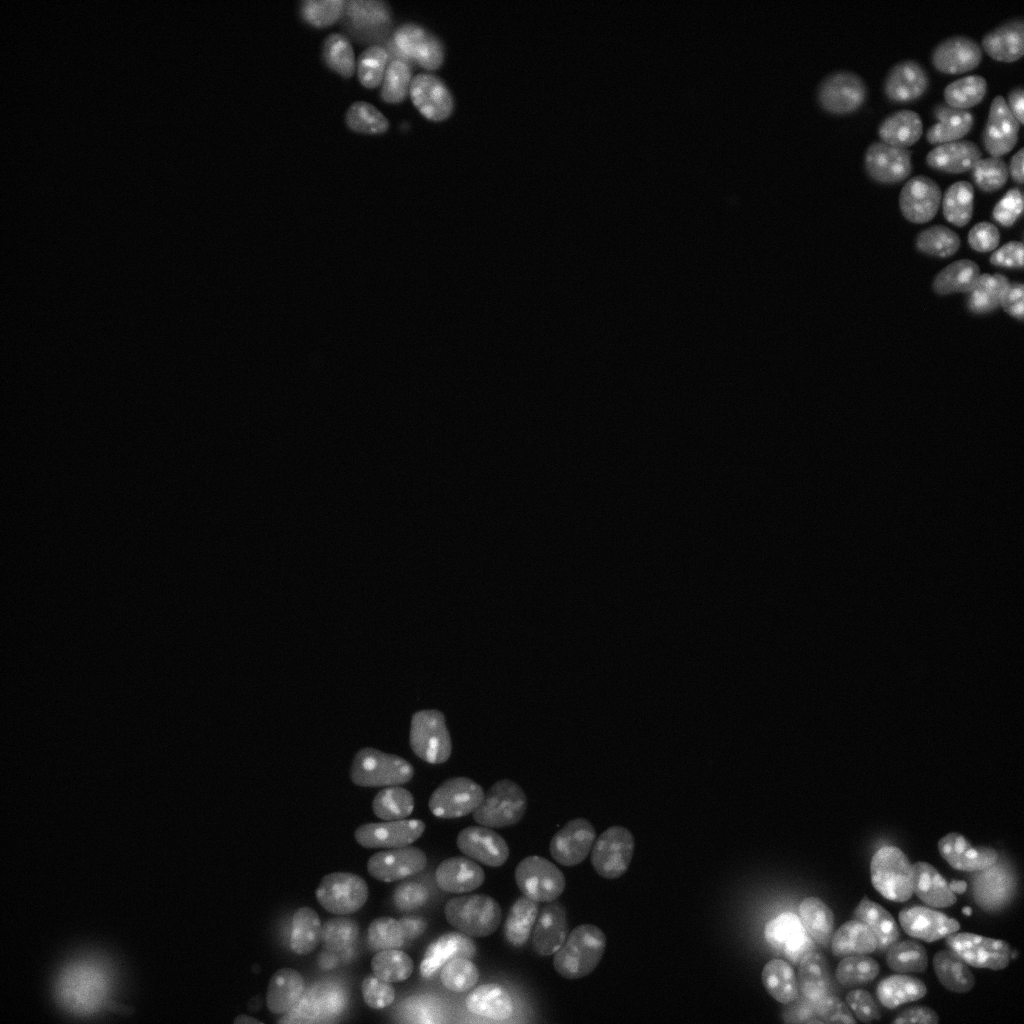

Supplement: Supplementary file 3 — Source Data [file 41467_2020_20757_MOESM3_ESM.zip › source_data/figure 1b-c images/HDAC5_woDox_images/Position010_t200_ch02.tif]

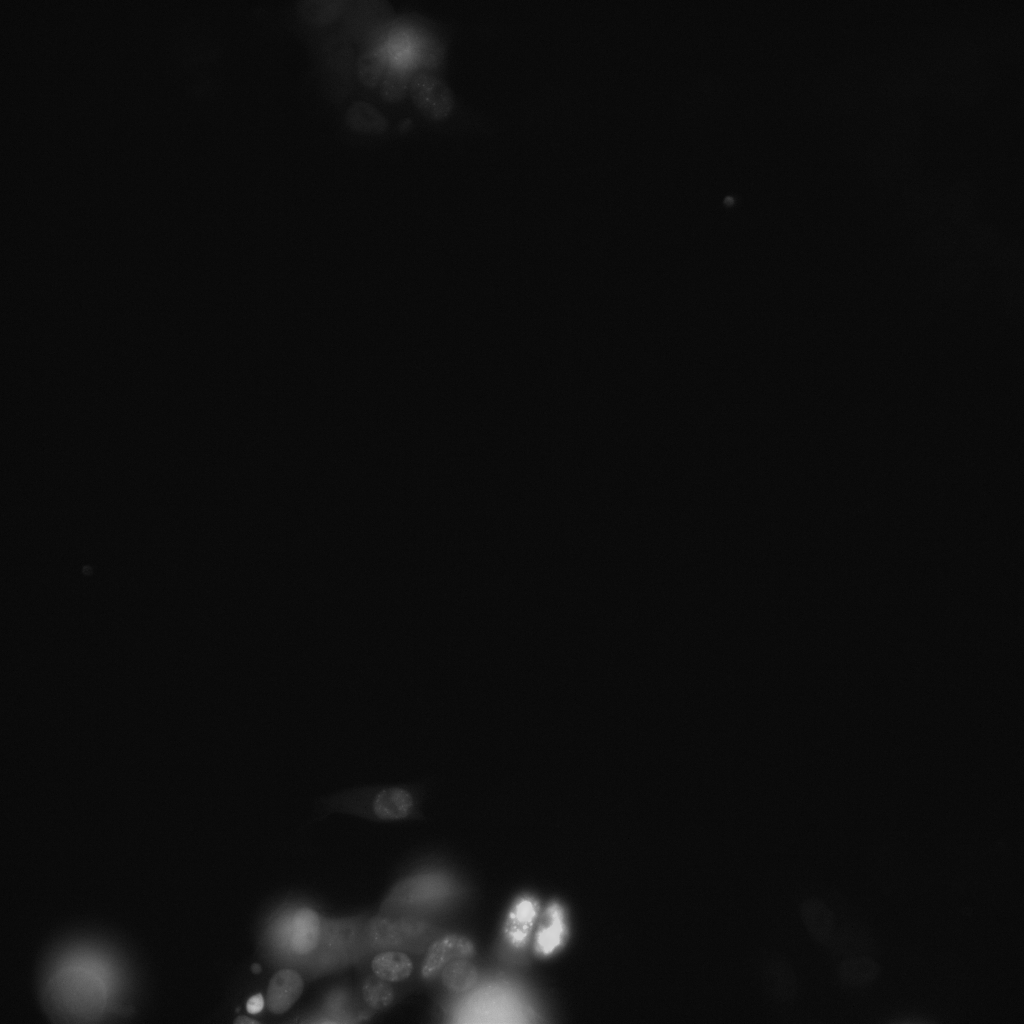

Supplement: Supplementary file 3 — Source Data [file 41467_2020_20757_MOESM3_ESM.zip › source_data/figure 1b-c images/HDAC5_woDox_images/Position010_t200_ch03.tif]

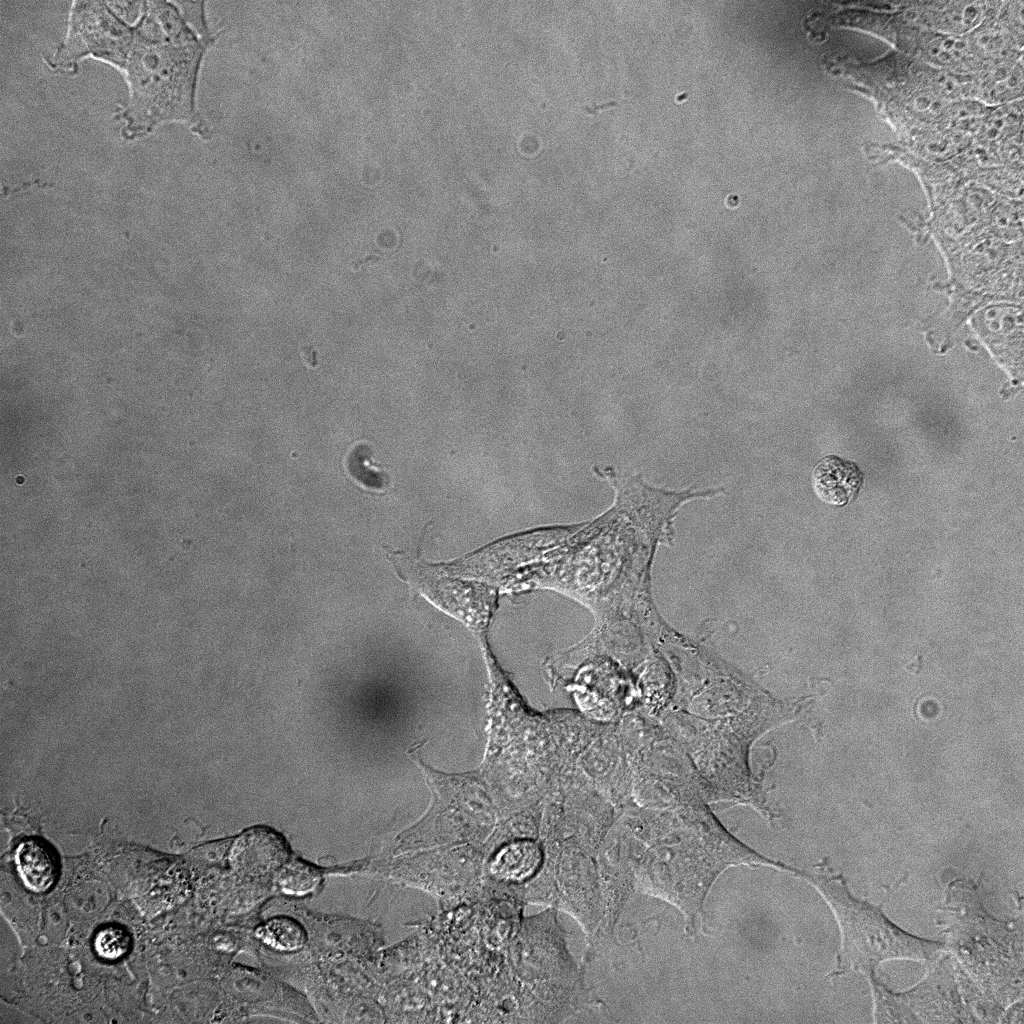

Supplement: Supplementary file 3 — Source Data [file 41467_2020_20757_MOESM3_ESM.zip › source_data/figure 1b-c images/HDAC5_woDox_images/Position010_t247_ch00.tif]

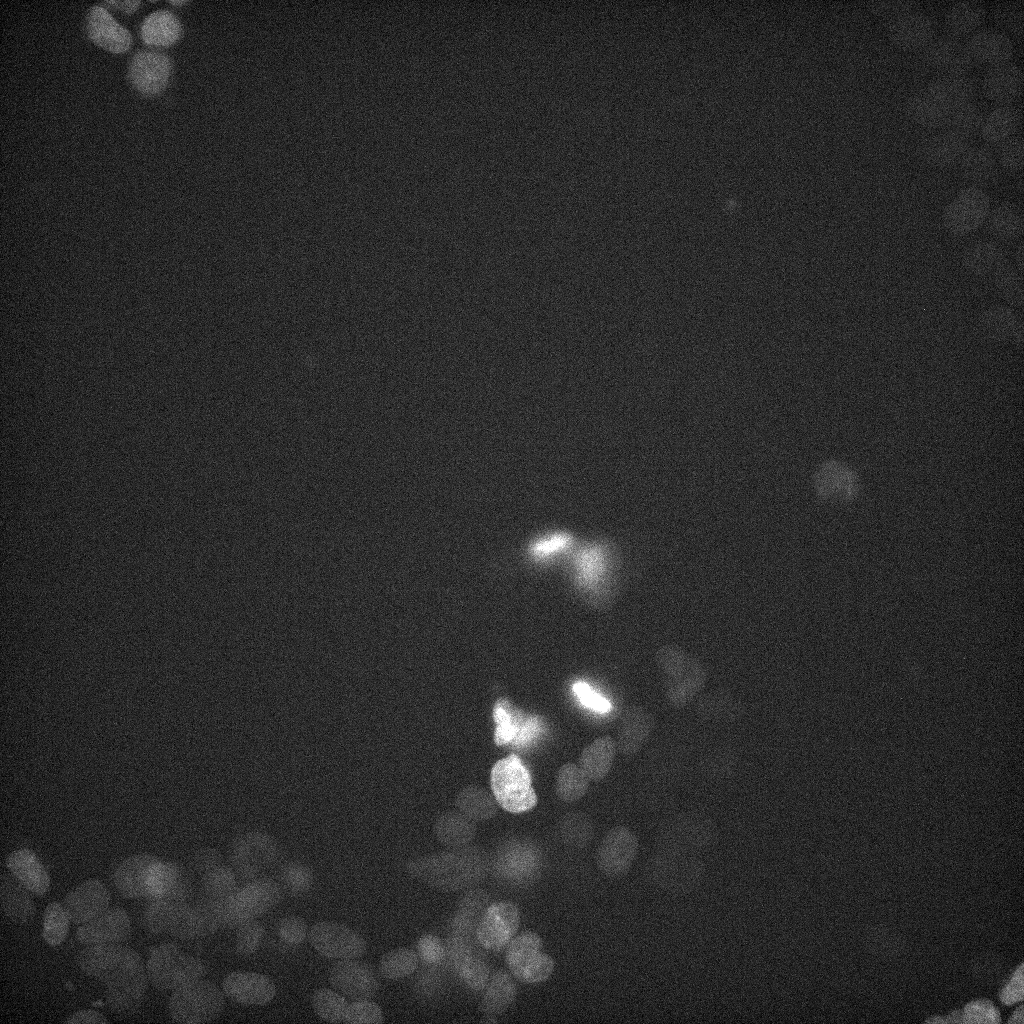

Supplement: Supplementary file 3 — Source Data [file 41467_2020_20757_MOESM3_ESM.zip › source_data/figure 1b-c images/HDAC5_woDox_images/Position010_t247_ch01.tif]

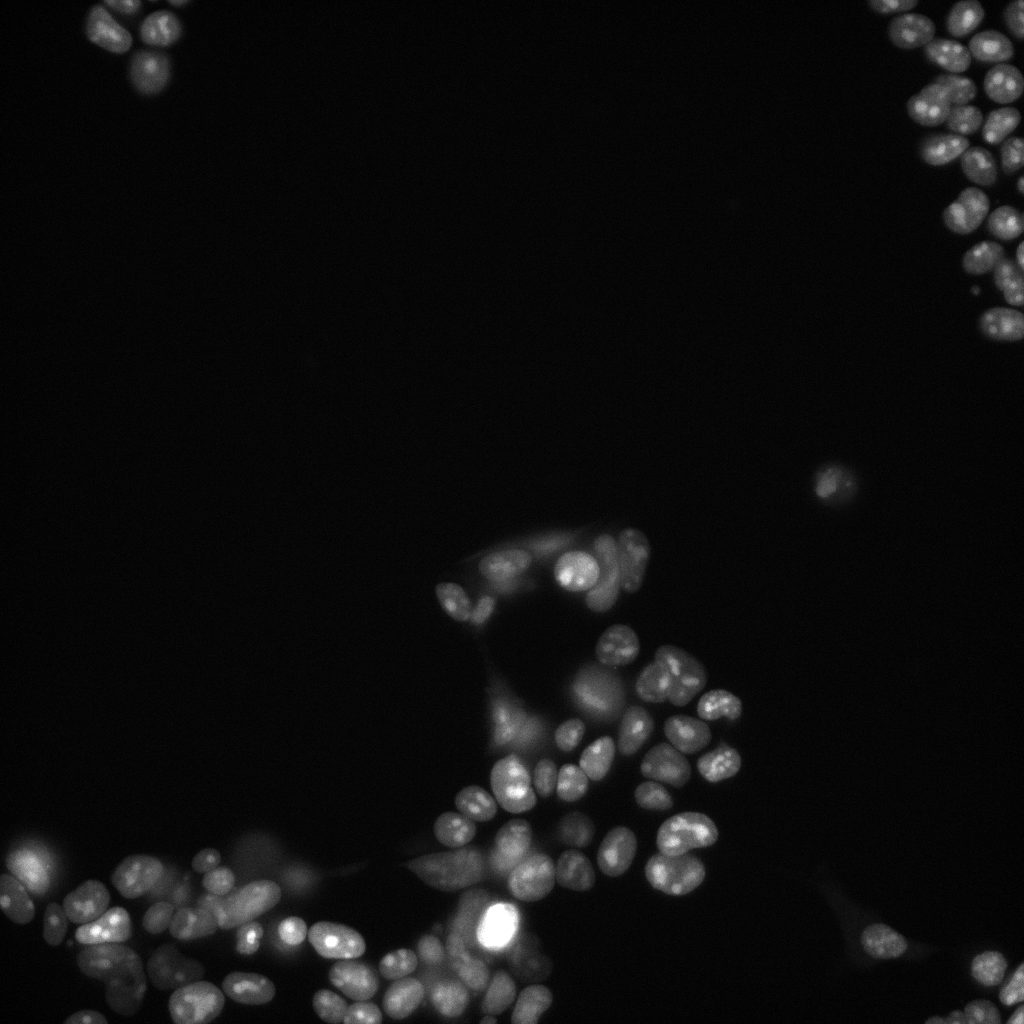

Supplement: Supplementary file 3 — Source Data [file 41467_2020_20757_MOESM3_ESM.zip › source_data/figure 1b-c images/HDAC5_woDox_images/Position010_t247_ch02.tif]

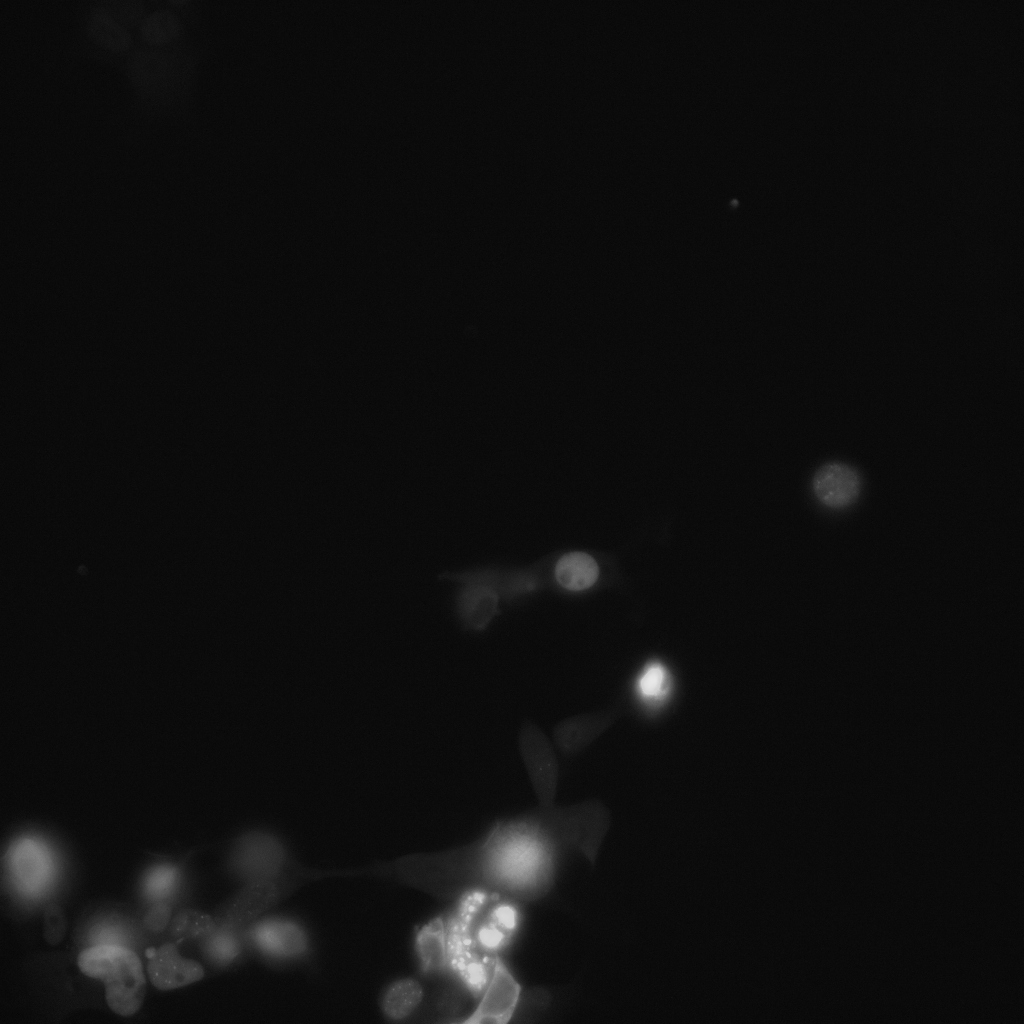

Supplement: Supplementary file 3 — Source Data [file 41467_2020_20757_MOESM3_ESM.zip › source_data/figure 1b-c images/HDAC5_woDox_images/Position010_t247_ch03.tif]

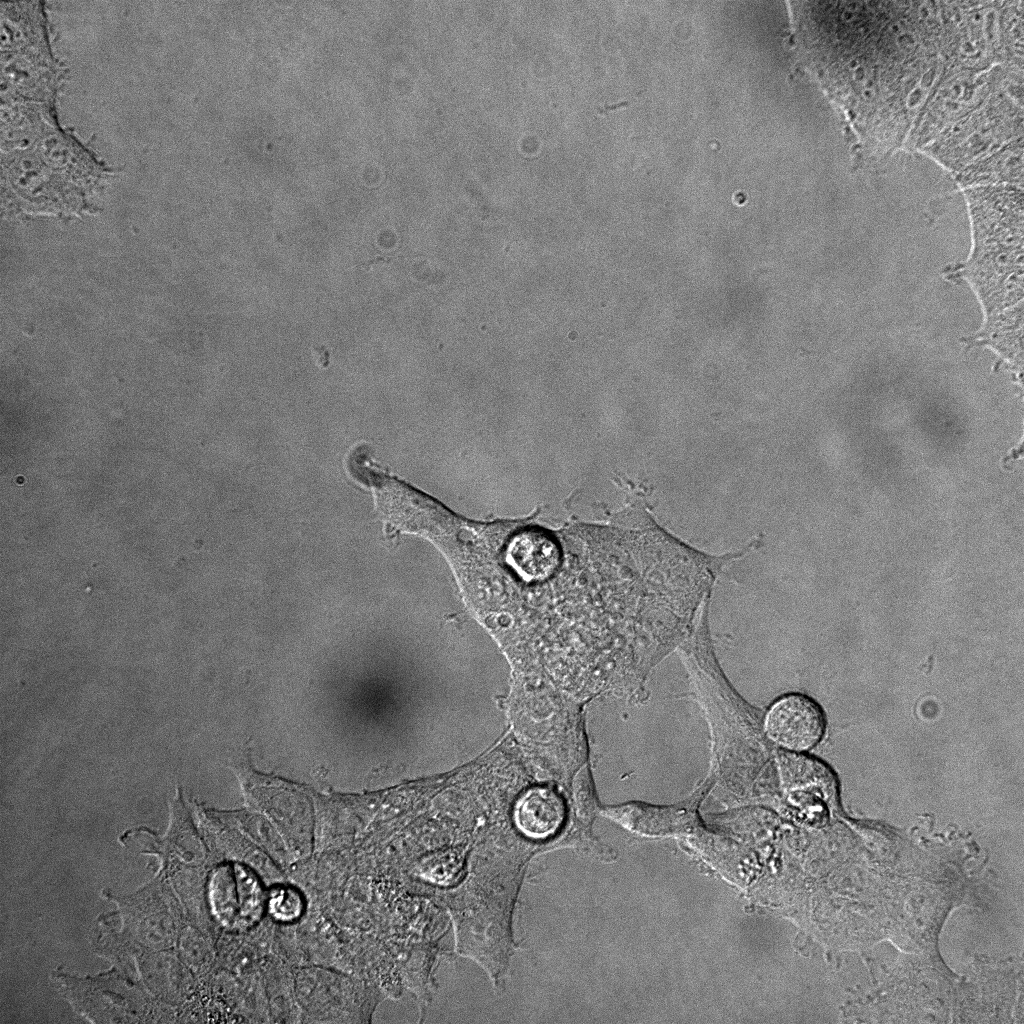

Supplement: Supplementary file 3 — Source Data [file 41467_2020_20757_MOESM3_ESM.zip › source_data/figure 1b-c images/HDAC5_woDox_images/Position010_t284_ch00.tif]

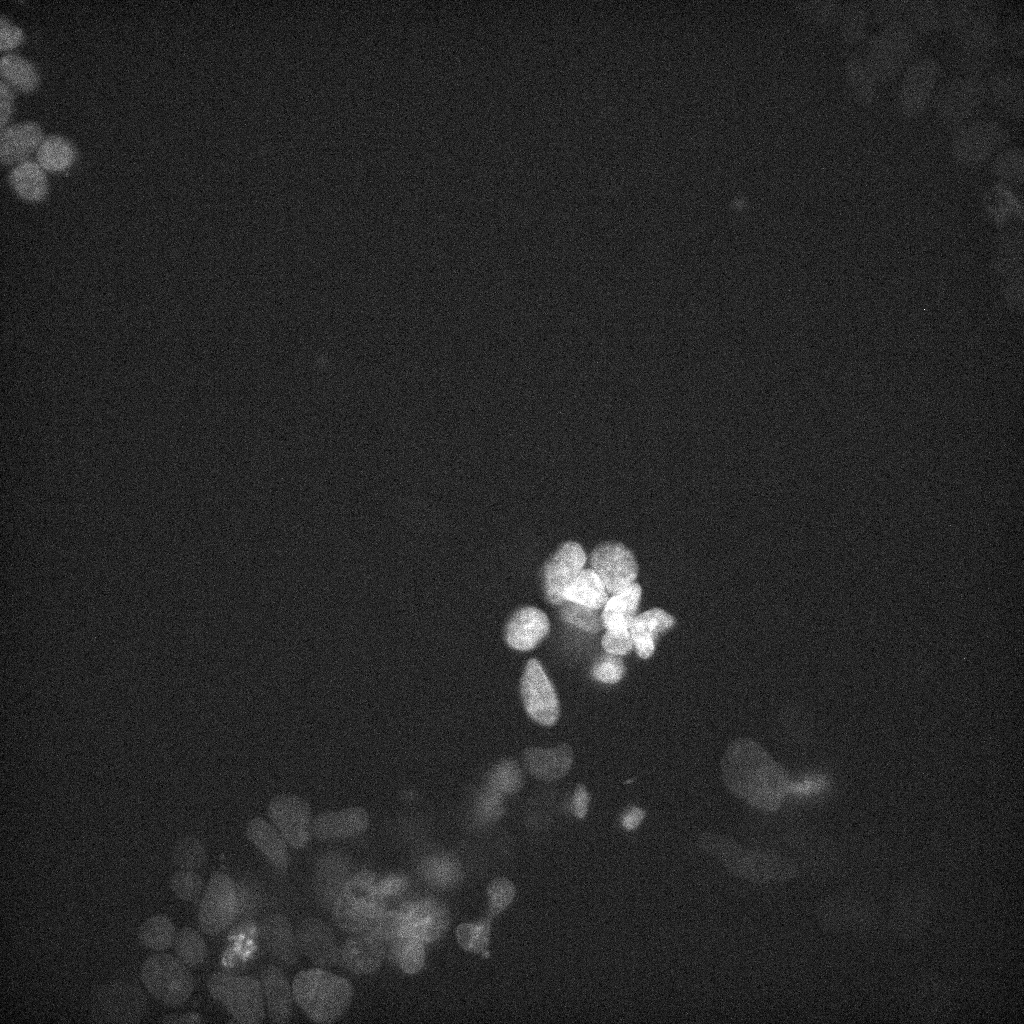

Supplement: Supplementary file 3 — Source Data [file 41467_2020_20757_MOESM3_ESM.zip › source_data/figure 1b-c images/HDAC5_woDox_images/Position010_t284_ch01.tif]

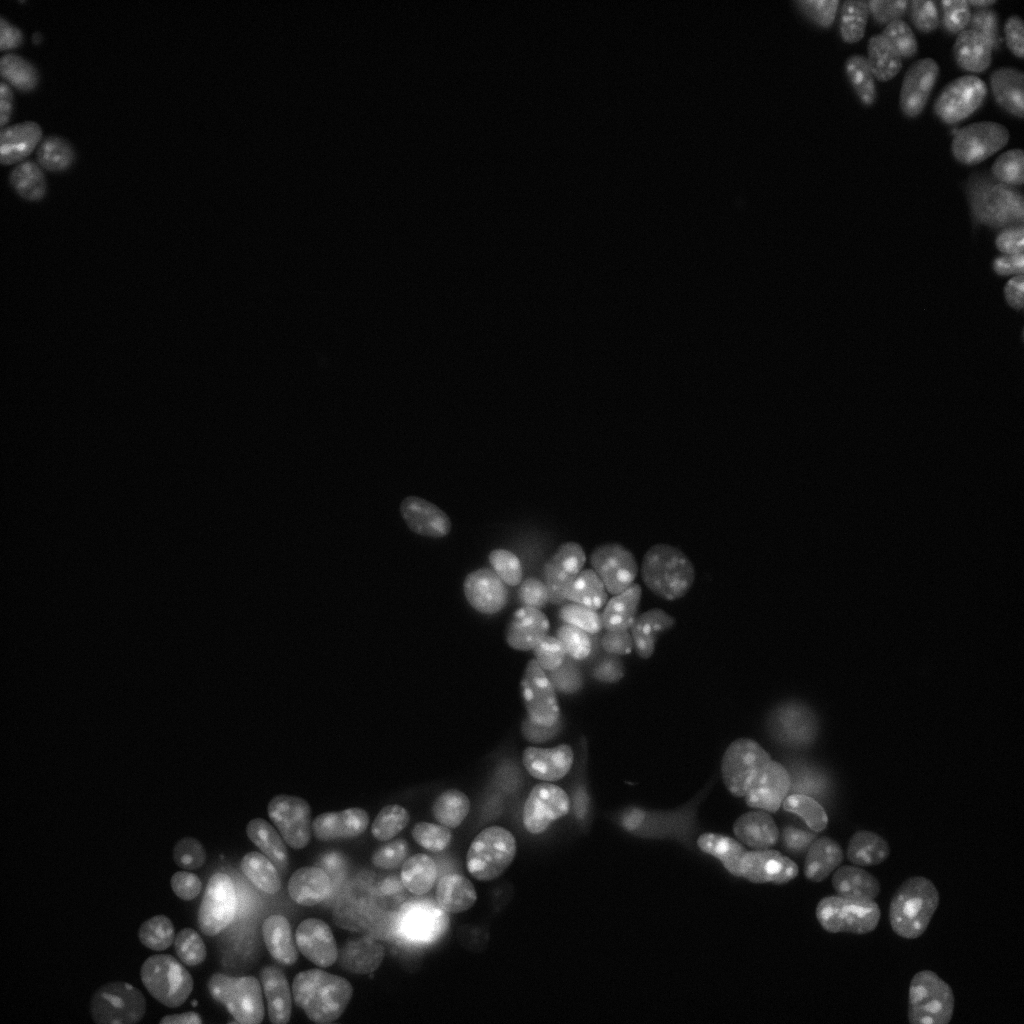

Supplement: Supplementary file 3 — Source Data [file 41467_2020_20757_MOESM3_ESM.zip › source_data/figure 1b-c images/HDAC5_woDox_images/Position010_t284_ch02.tif]

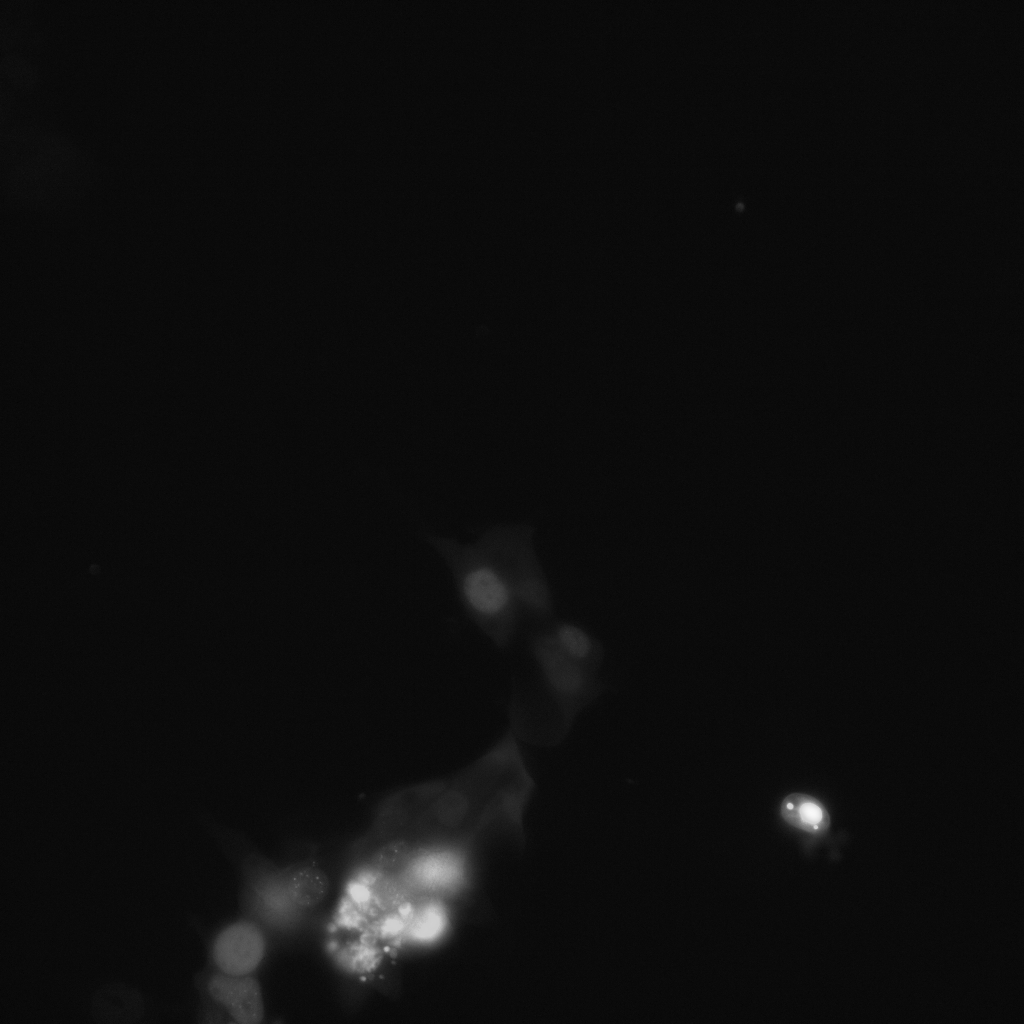

Supplement: Supplementary file 3 — Source Data [file 41467_2020_20757_MOESM3_ESM.zip › source_data/figure 1b-c images/HDAC5_woDox_images/Position010_t284_ch03.tif]

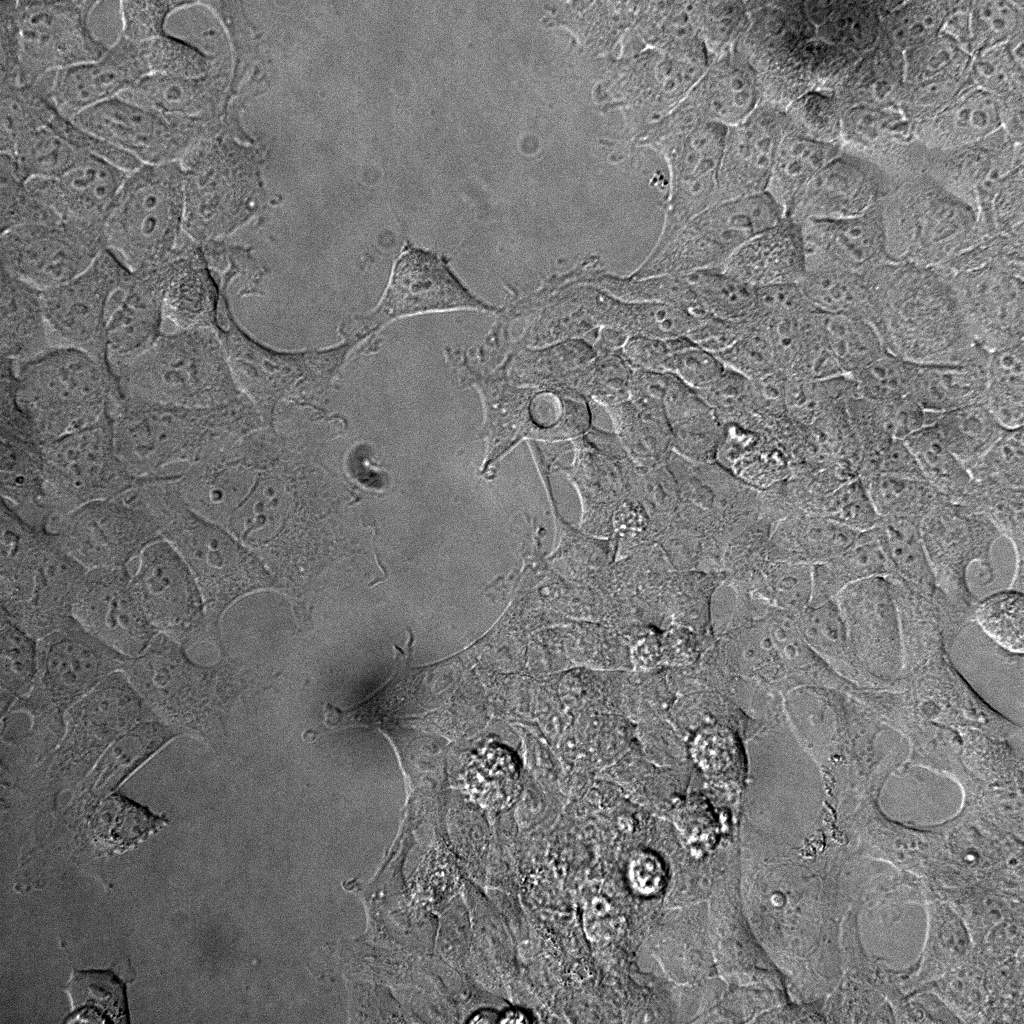

Supplement: Supplementary file 3 — Source Data [file 41467_2020_20757_MOESM3_ESM.zip › source_data/figure 1b-c images/HDAC5_woDox_images/Position010_t340_ch00.tif]

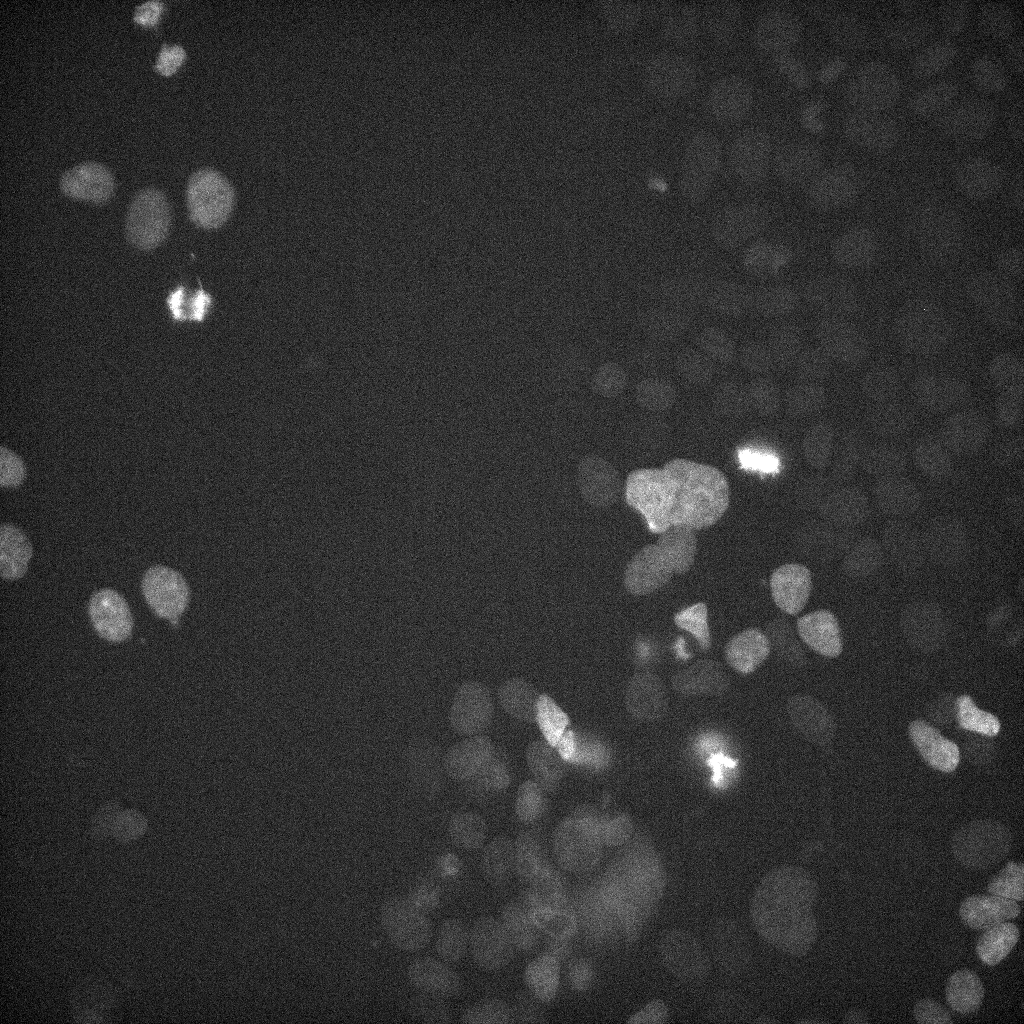

Supplement: Supplementary file 3 — Source Data [file 41467_2020_20757_MOESM3_ESM.zip › source_data/figure 1b-c images/HDAC5_woDox_images/Position010_t340_ch01.tif]

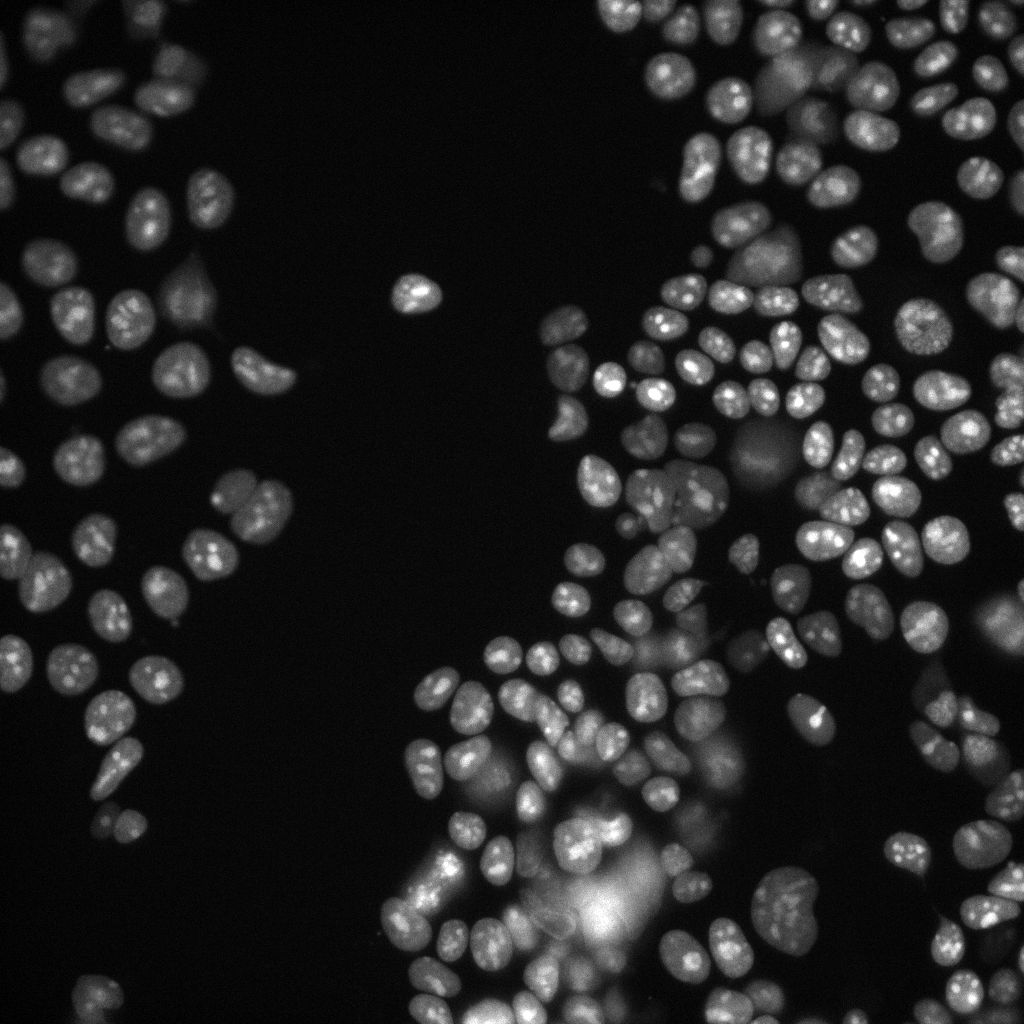

Supplement: Supplementary file 3 — Source Data [file 41467_2020_20757_MOESM3_ESM.zip › source_data/figure 1b-c images/HDAC5_woDox_images/Position010_t340_ch02.tif]

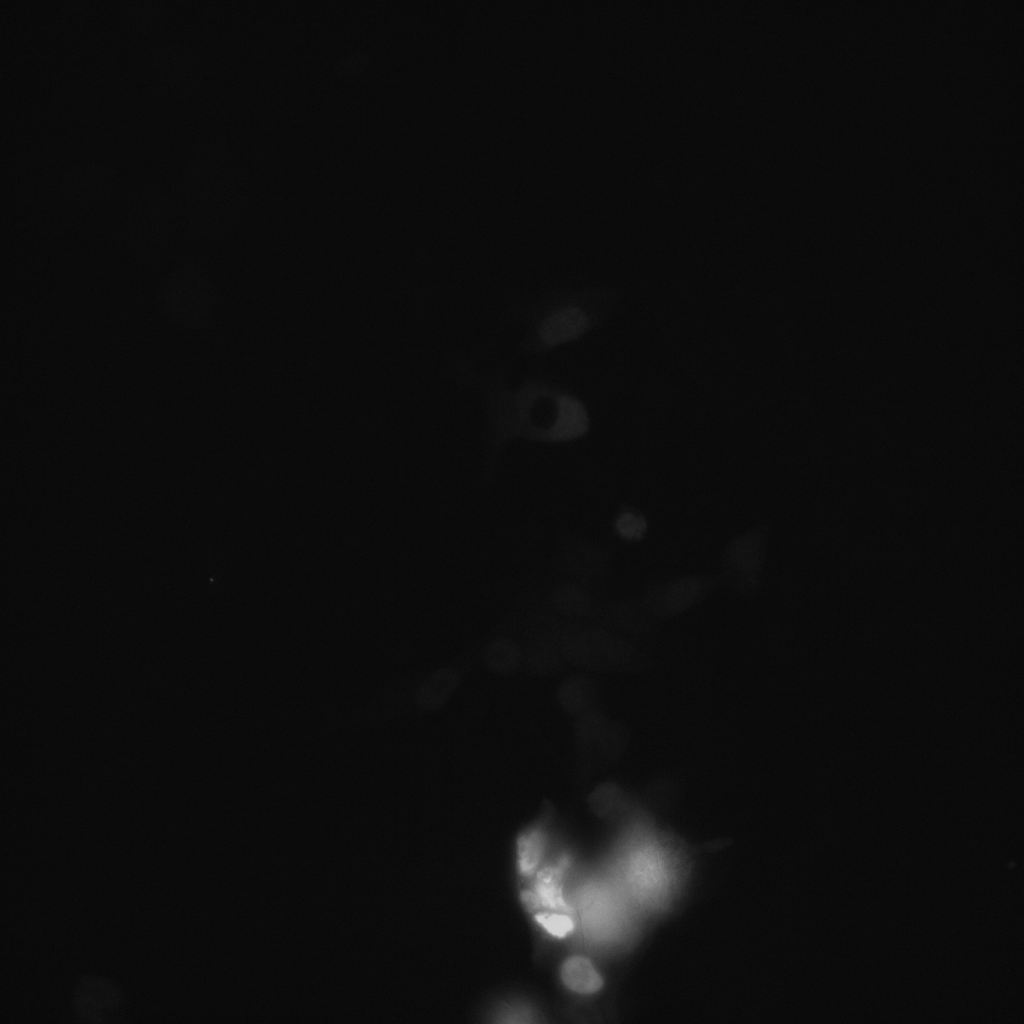

Supplement: Supplementary file 3 — Source Data [file 41467_2020_20757_MOESM3_ESM.zip › source_data/figure 1b-c images/HDAC5_woDox_images/Position010_t340_ch03.tif]

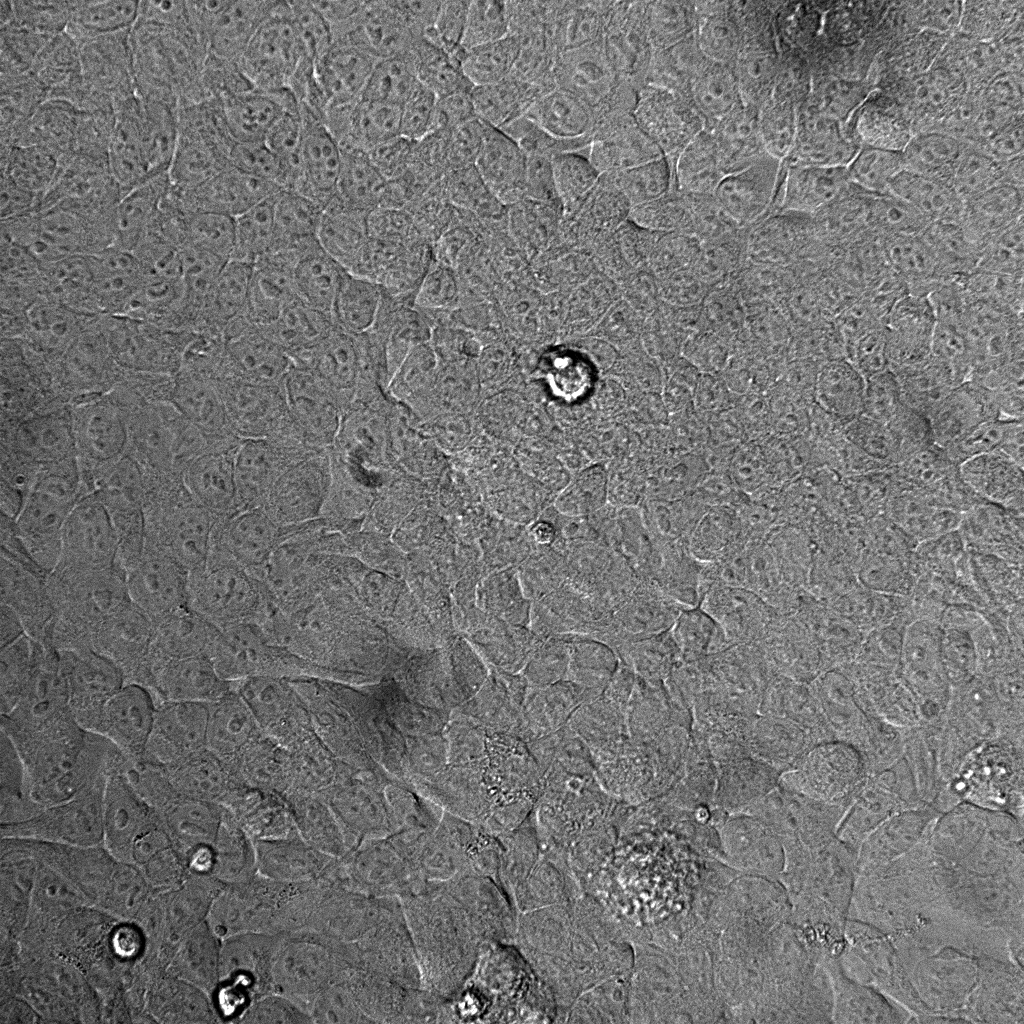

Supplement: Supplementary file 3 — Source Data [file 41467_2020_20757_MOESM3_ESM.zip › source_data/figure 1b-c images/HDAC5_woDox_images/Position010_t396_ch00.tif]

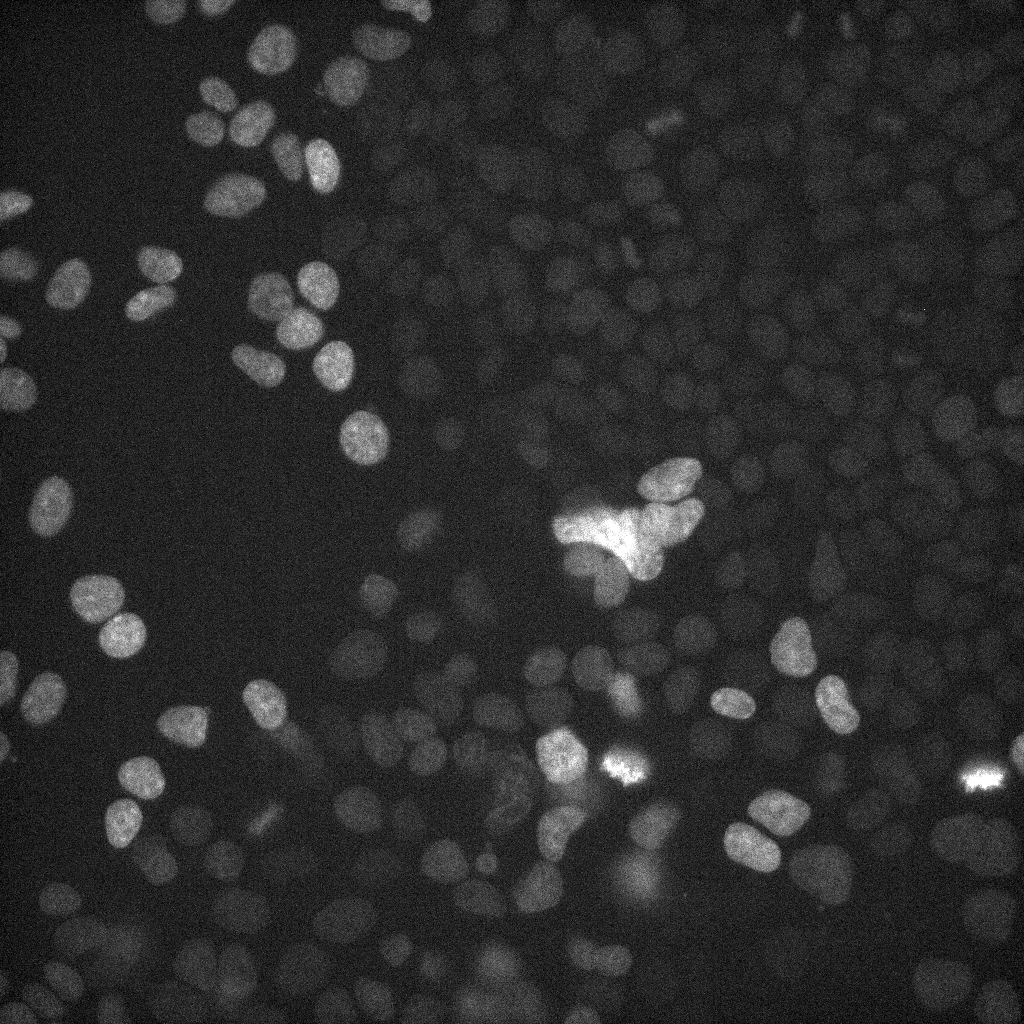

Supplement: Supplementary file 3 — Source Data [file 41467_2020_20757_MOESM3_ESM.zip › source_data/figure 1b-c images/HDAC5_woDox_images/Position010_t396_ch01.tif]

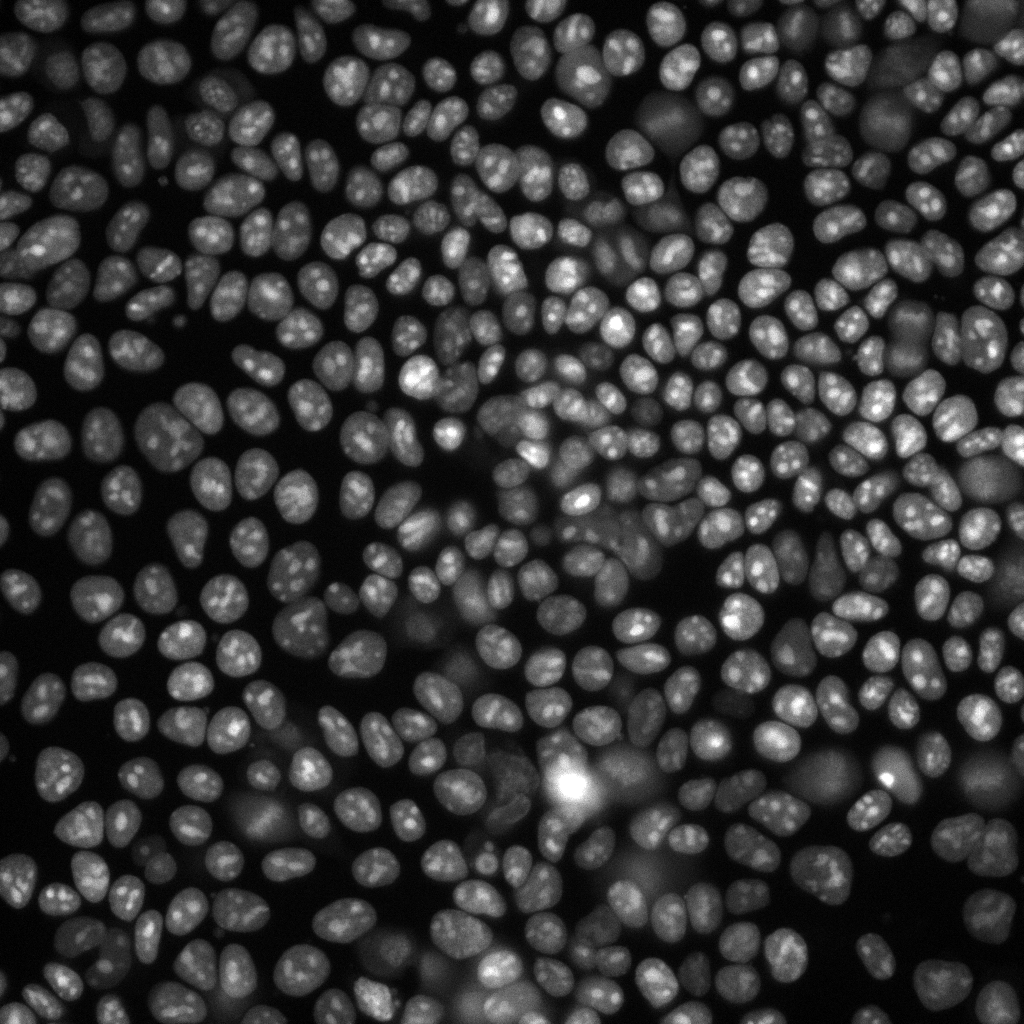

Supplement: Supplementary file 3 — Source Data [file 41467_2020_20757_MOESM3_ESM.zip › source_data/figure 1b-c images/HDAC5_woDox_images/Position010_t396_ch02.tif]

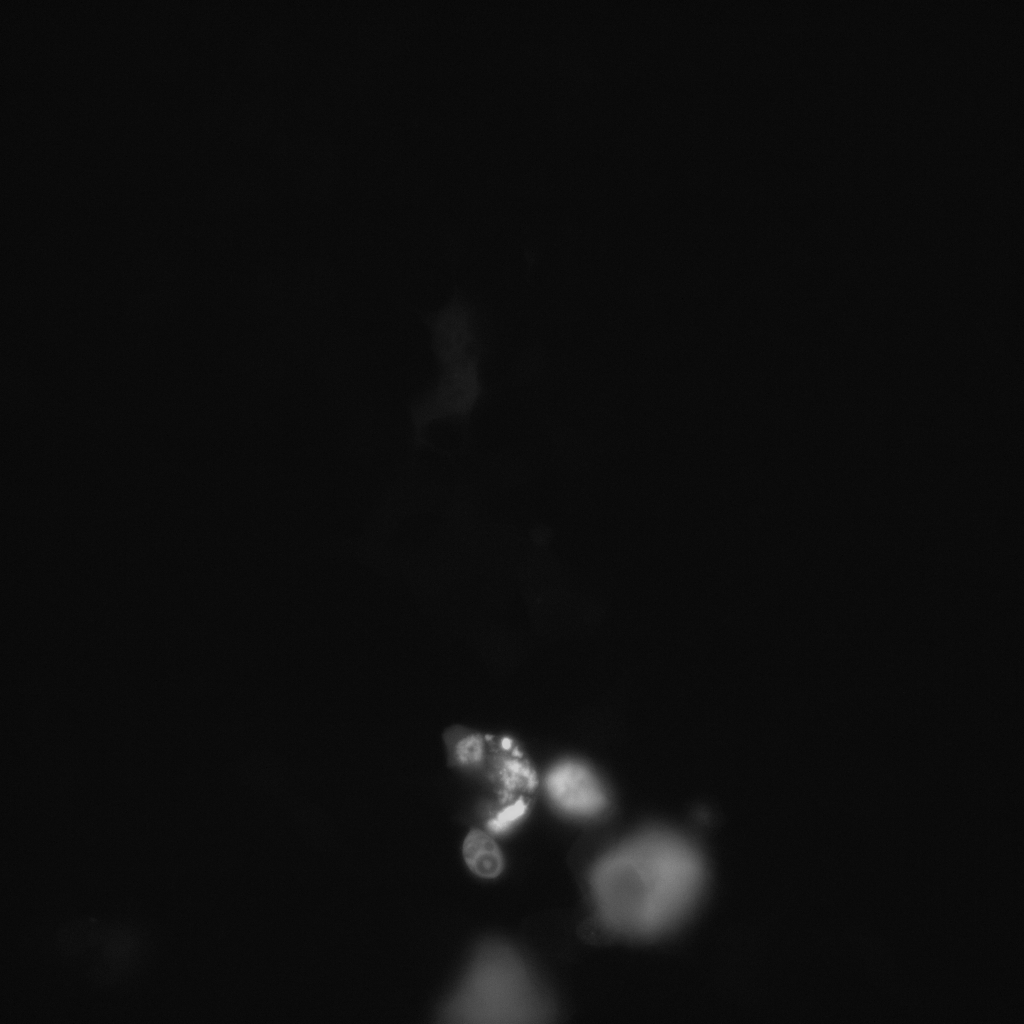

Supplement: Supplementary file 3 — Source Data [file 41467_2020_20757_MOESM3_ESM.zip › source_data/figure 1b-c images/HDAC5_woDox_images/Position010_t396_ch03.tif]

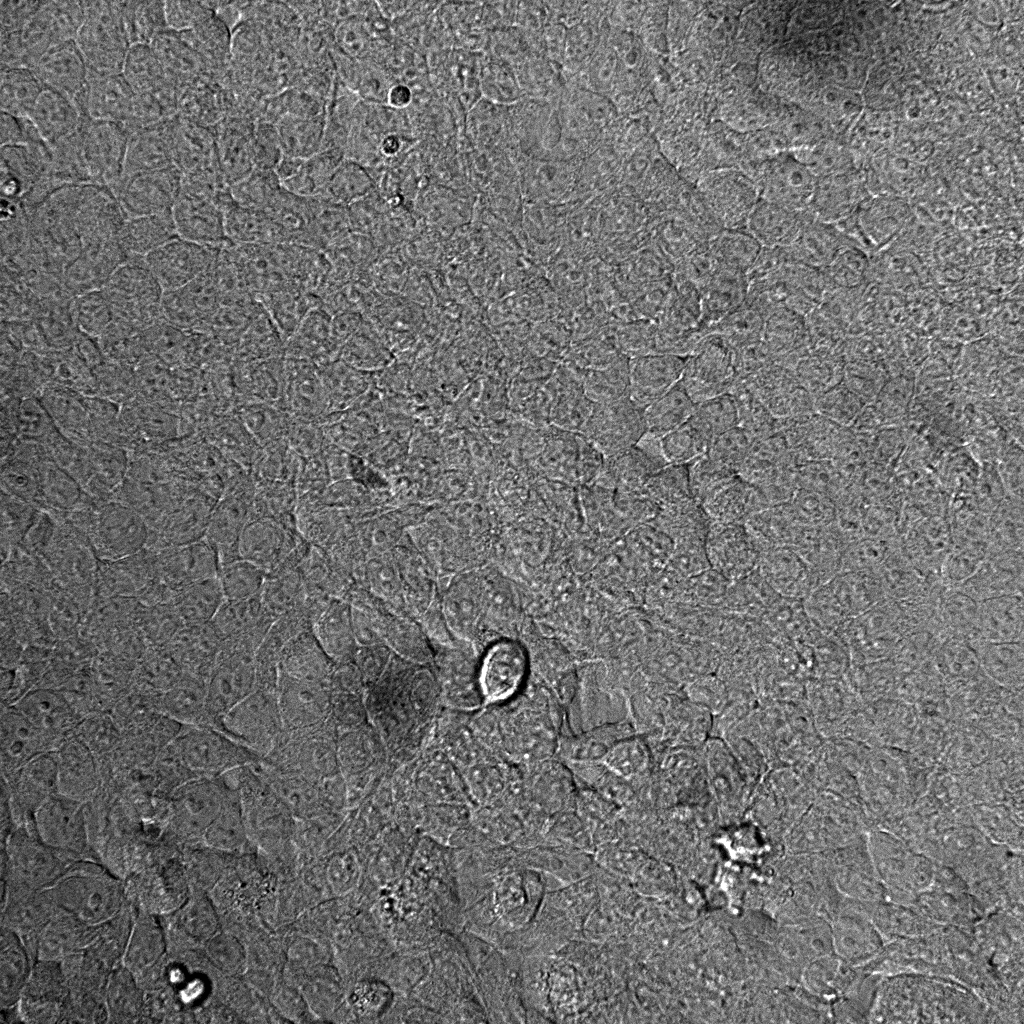

Supplement: Supplementary file 3 — Source Data [file 41467_2020_20757_MOESM3_ESM.zip › source_data/figure 1b-c images/HDAC5_woDox_images/Position010_t427_ch00.tif]

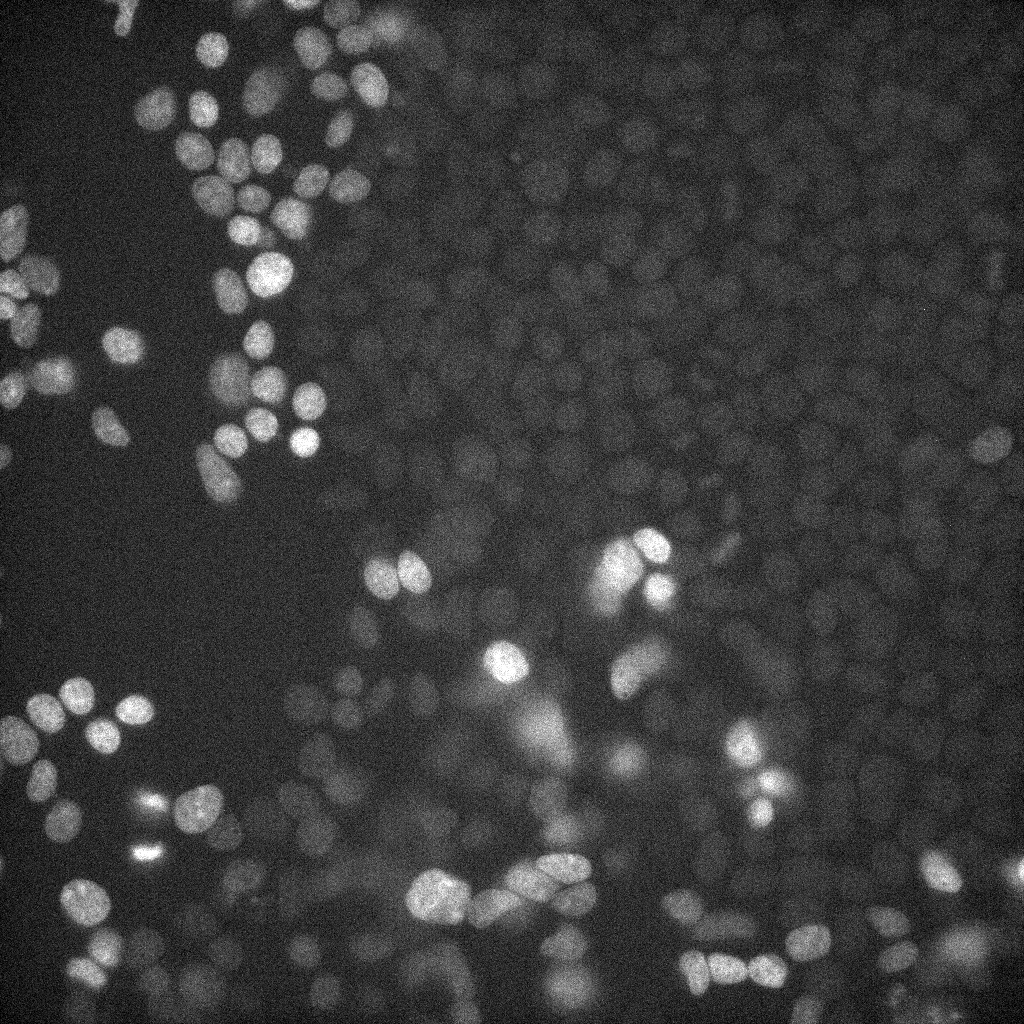

Supplement: Supplementary file 3 — Source Data [file 41467_2020_20757_MOESM3_ESM.zip › source_data/figure 1b-c images/HDAC5_woDox_images/Position010_t427_ch01.tif]

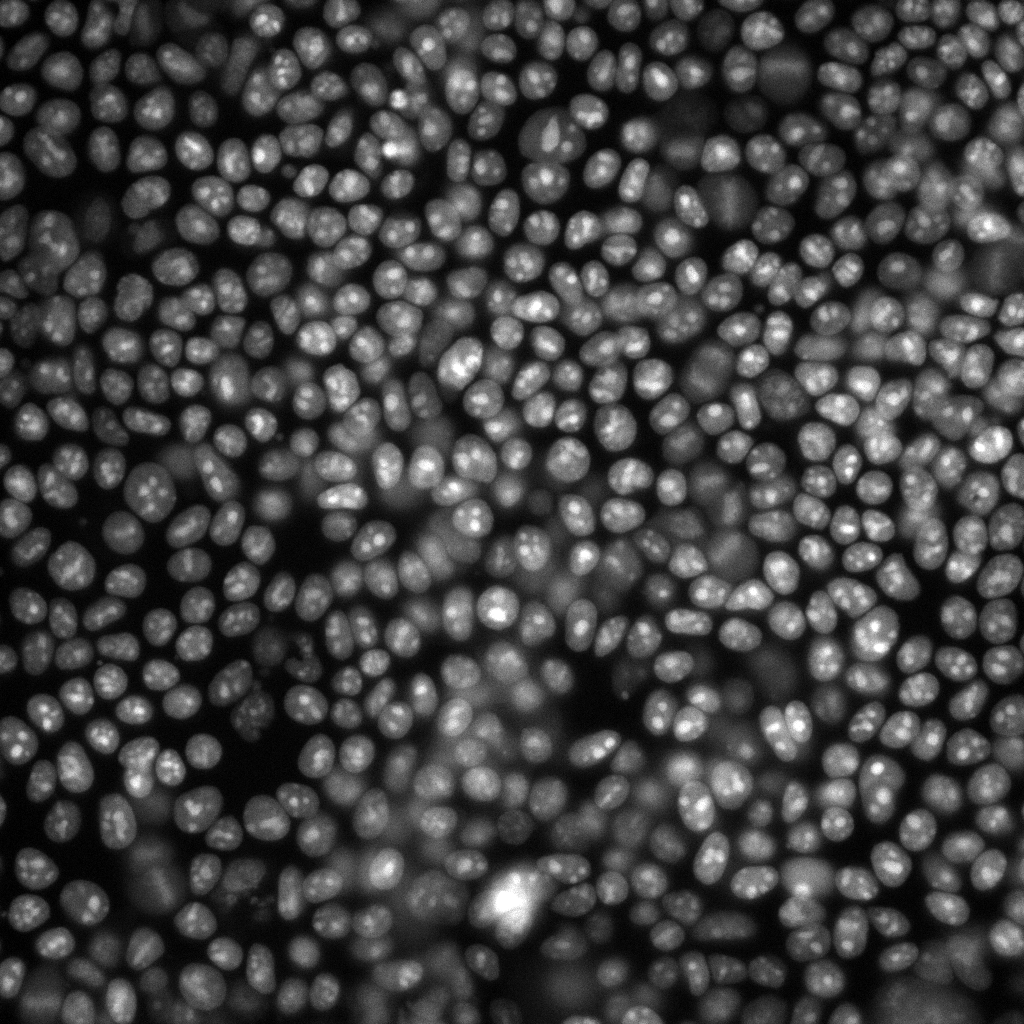

Supplement: Supplementary file 3 — Source Data [file 41467_2020_20757_MOESM3_ESM.zip › source_data/figure 1b-c images/HDAC5_woDox_images/Position010_t427_ch02.tif]

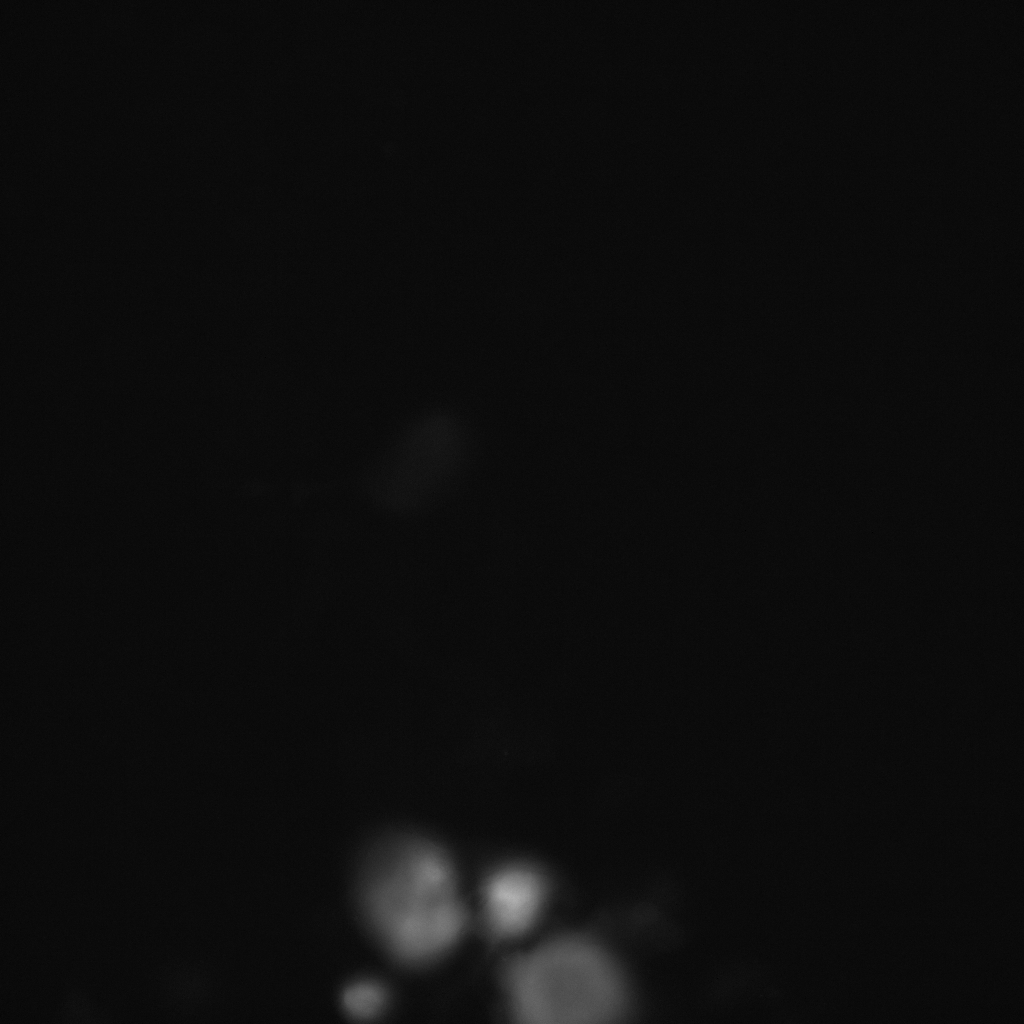

Supplement: Supplementary file 3 — Source Data [file 41467_2020_20757_MOESM3_ESM.zip › source_data/figure 1b-c images/HDAC5_woDox_images/Position010_t427_ch03.tif]

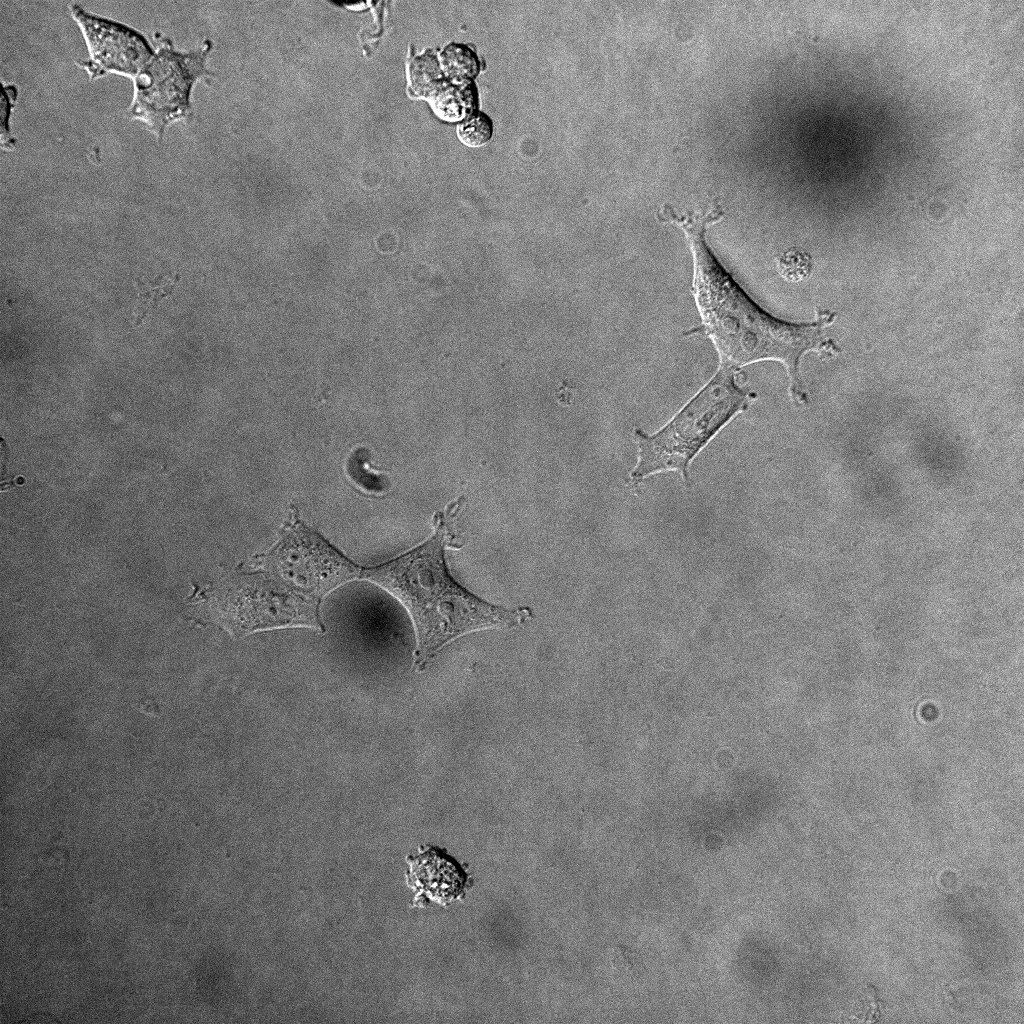

Supplement: Supplementary file 3 — Source Data [file 41467_2020_20757_MOESM3_ESM.zip › source_data/figure 1b-c images/HP1a_woDox_images/Position029_t004_ch00.tif]

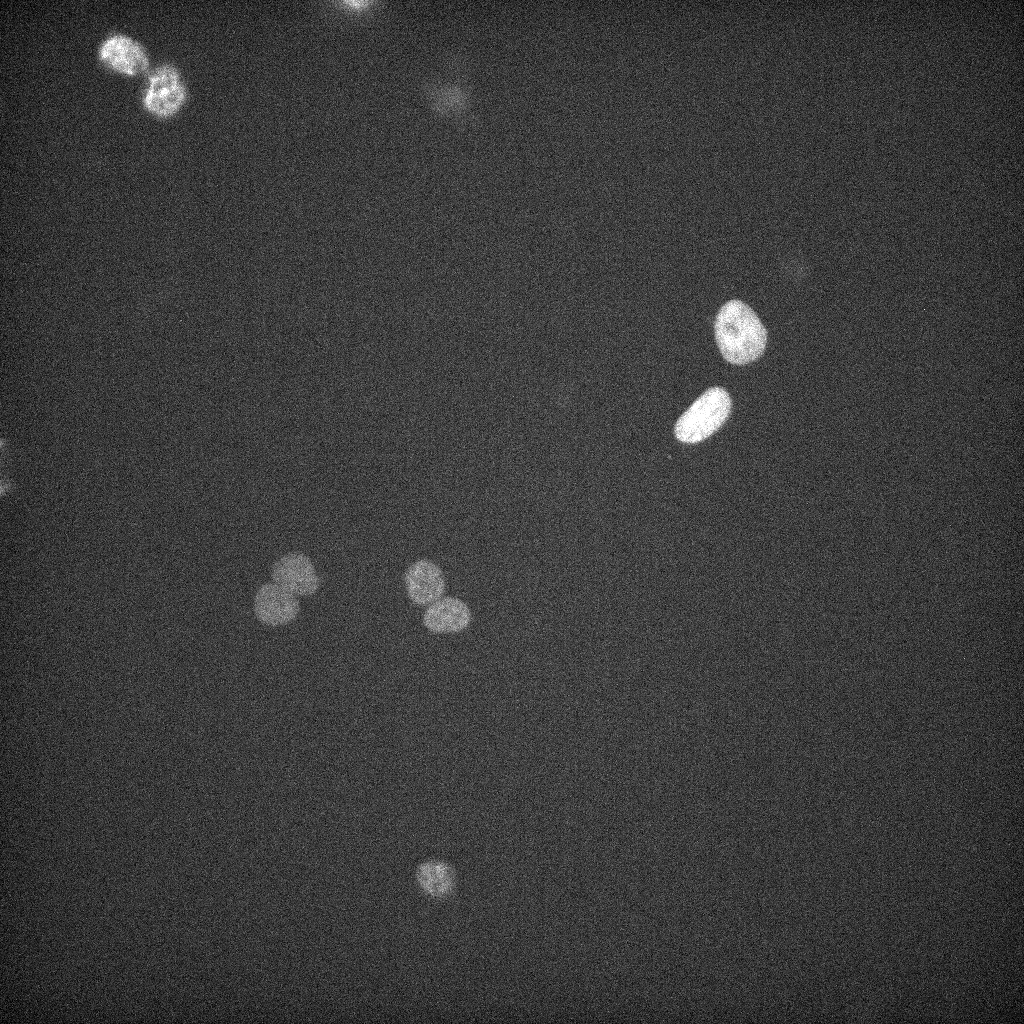

Supplement: Supplementary file 3 — Source Data [file 41467_2020_20757_MOESM3_ESM.zip › source_data/figure 1b-c images/HP1a_woDox_images/Position029_t004_ch01.tif]

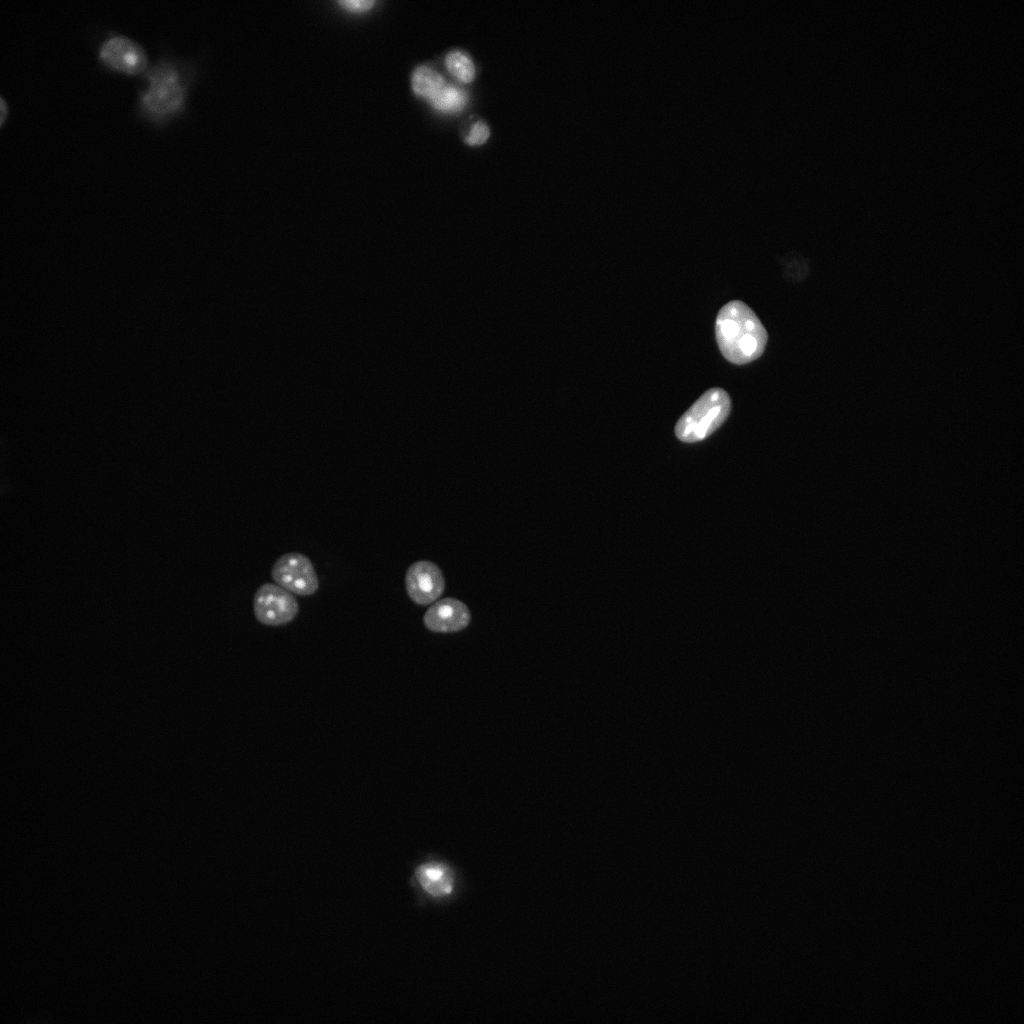

Supplement: Supplementary file 3 — Source Data [file 41467_2020_20757_MOESM3_ESM.zip › source_data/figure 1b-c images/HP1a_woDox_images/Position029_t004_ch02.tif]

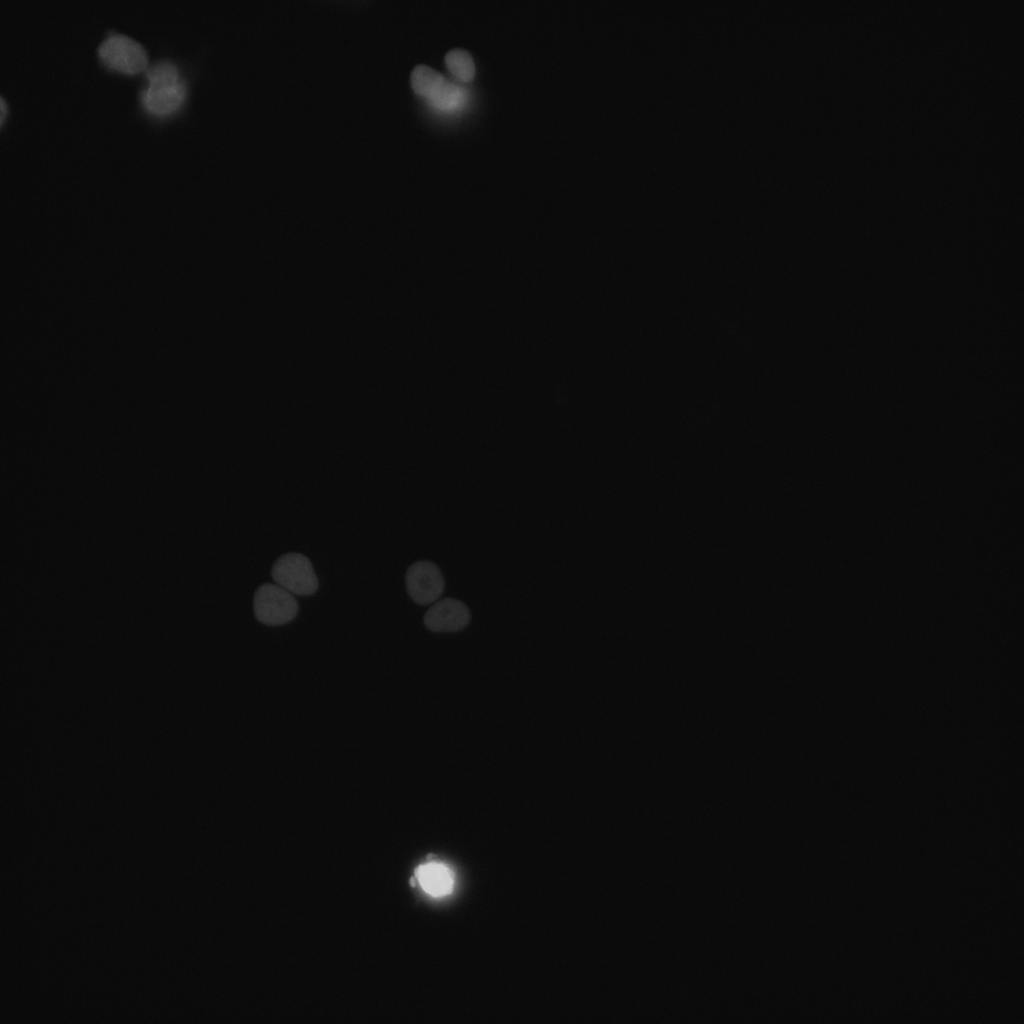

Supplement: Supplementary file 3 — Source Data [file 41467_2020_20757_MOESM3_ESM.zip › source_data/figure 1b-c images/HP1a_woDox_images/Position029_t004_ch03.tif]

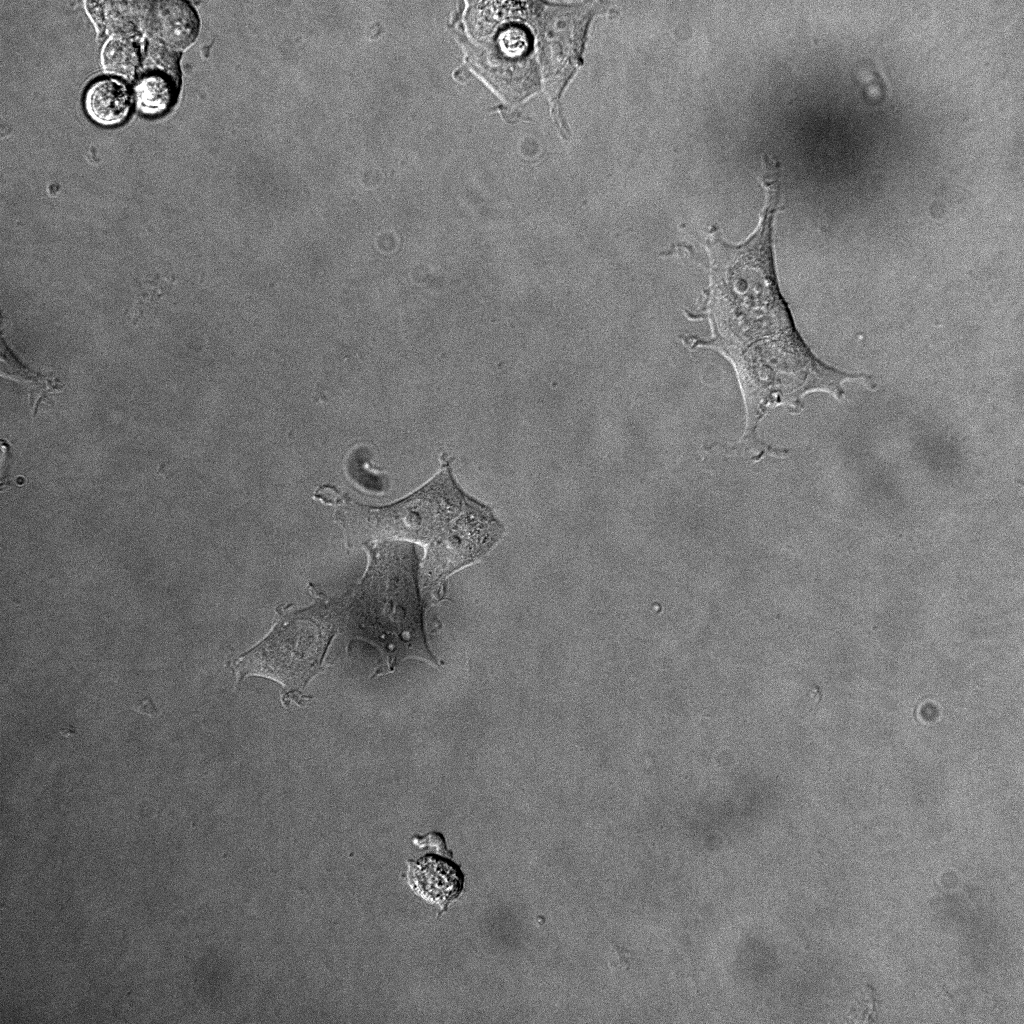

Supplement: Supplementary file 3 — Source Data [file 41467_2020_20757_MOESM3_ESM.zip › source_data/figure 1b-c images/HP1a_woDox_images/Position029_t040_ch00.tif]

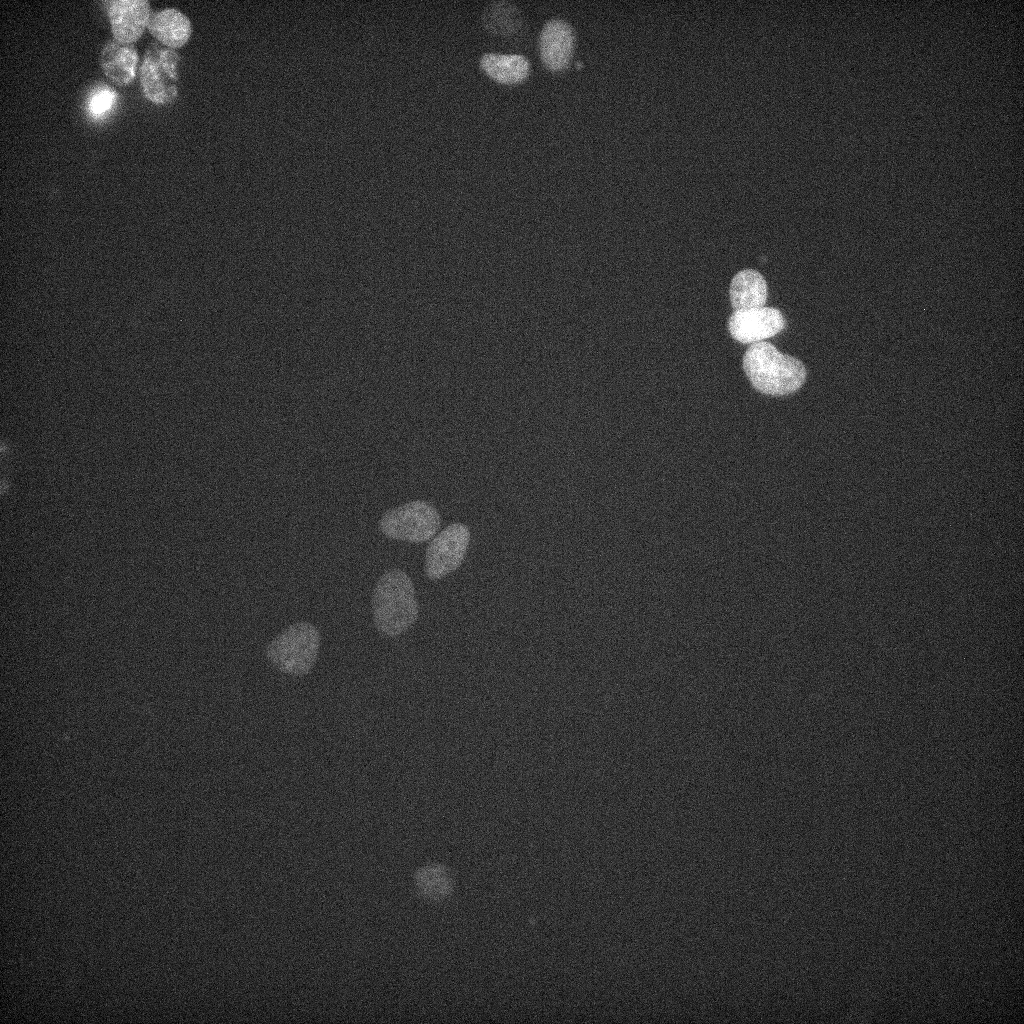

Supplement: Supplementary file 3 — Source Data [file 41467_2020_20757_MOESM3_ESM.zip › source_data/figure 1b-c images/HP1a_woDox_images/Position029_t040_ch01.tif]

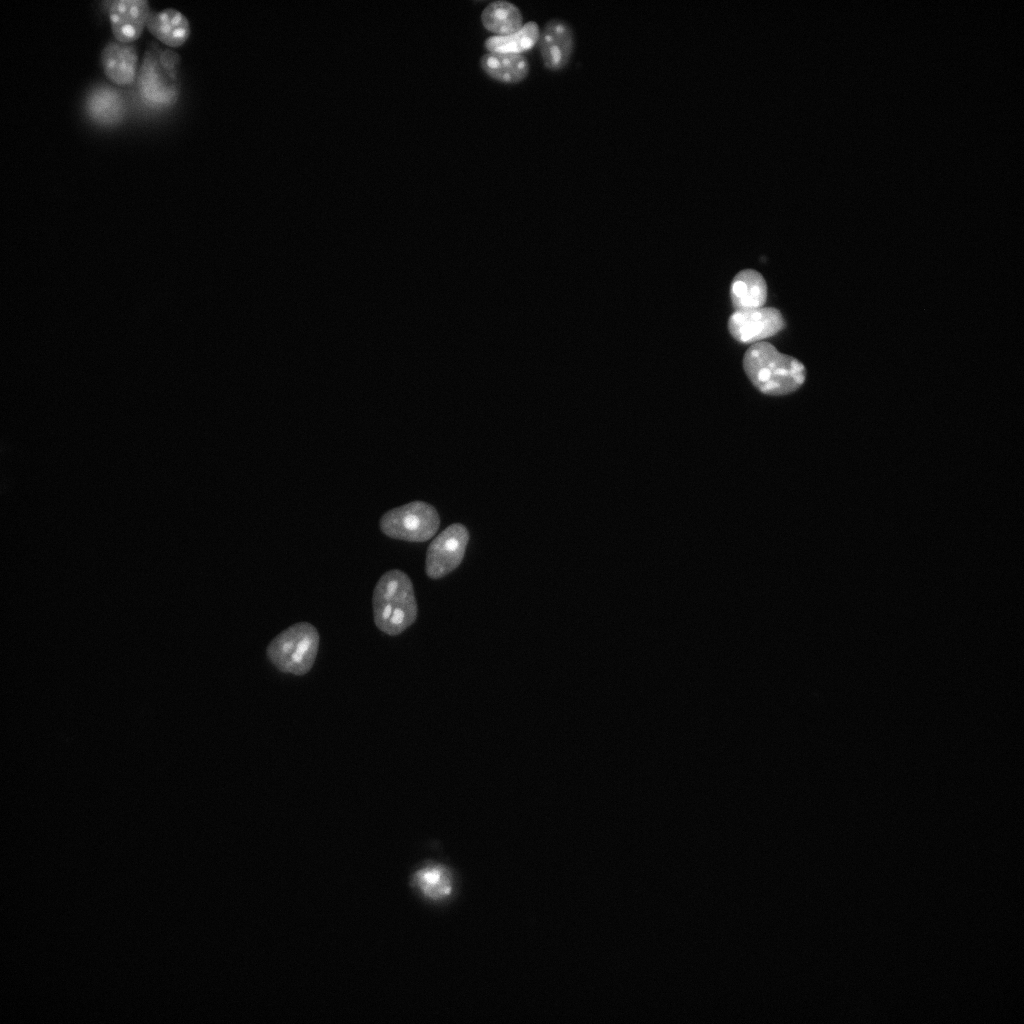

Supplement: Supplementary file 3 — Source Data [file 41467_2020_20757_MOESM3_ESM.zip › source_data/figure 1b-c images/HP1a_woDox_images/Position029_t040_ch02.tif]

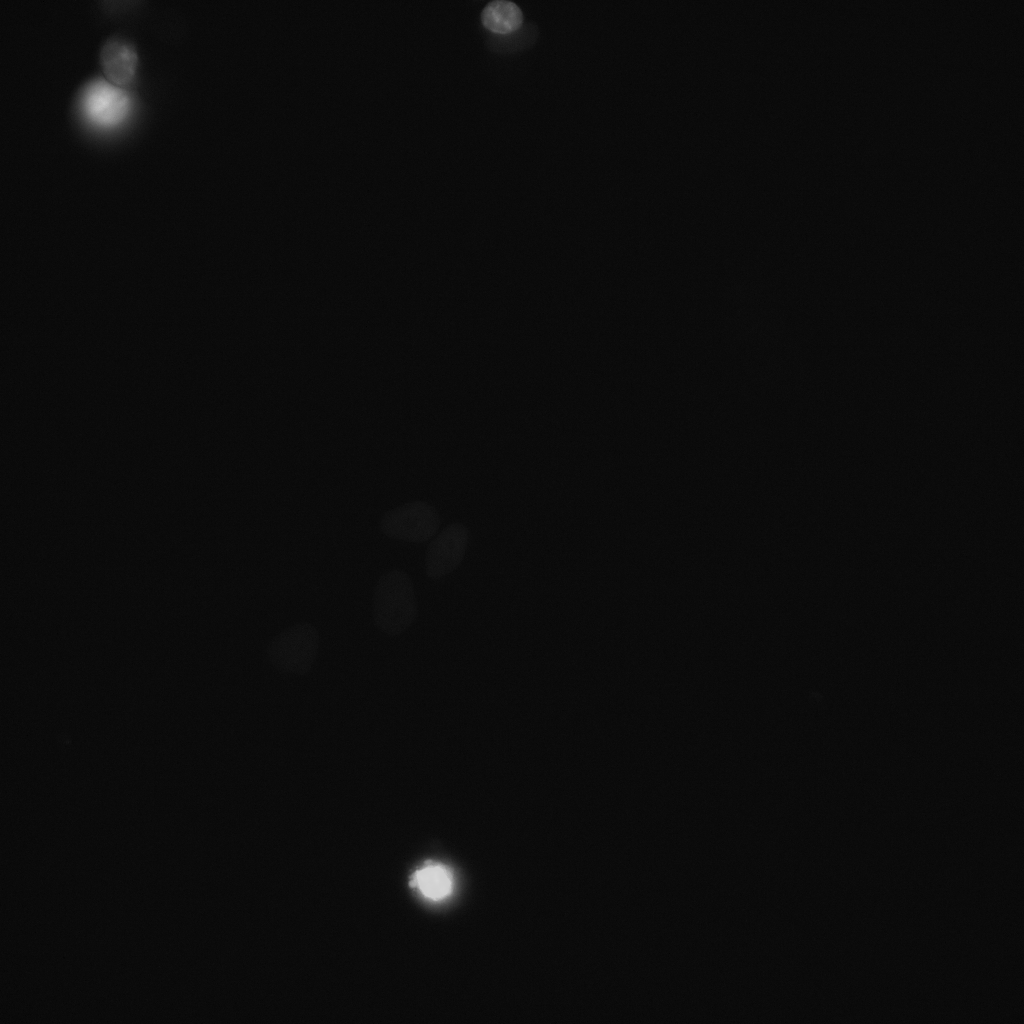

Supplement: Supplementary file 3 — Source Data [file 41467_2020_20757_MOESM3_ESM.zip › source_data/figure 1b-c images/HP1a_woDox_images/Position029_t040_ch03.tif]

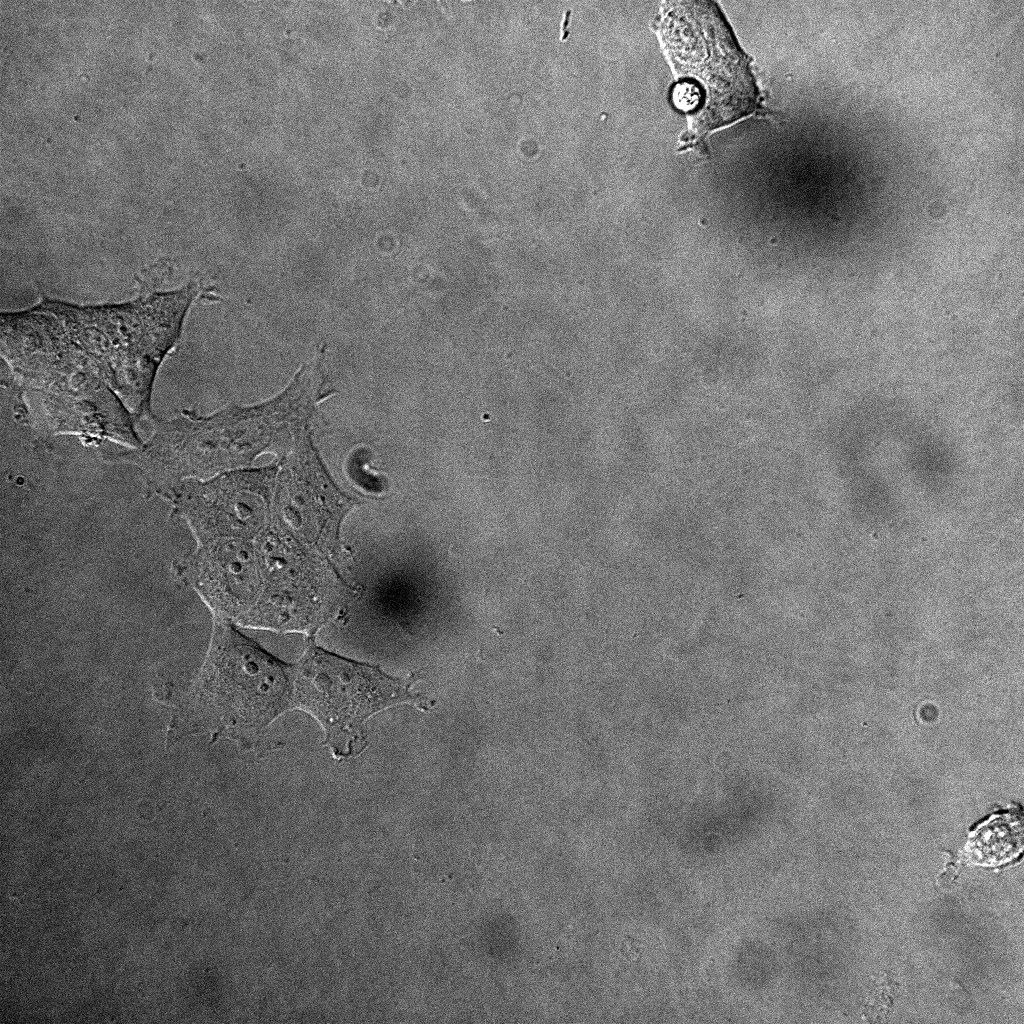

Supplement: Supplementary file 3 — Source Data [file 41467_2020_20757_MOESM3_ESM.zip › source_data/figure 1b-c images/HP1a_woDox_images/Position029_t102_ch00.tif]

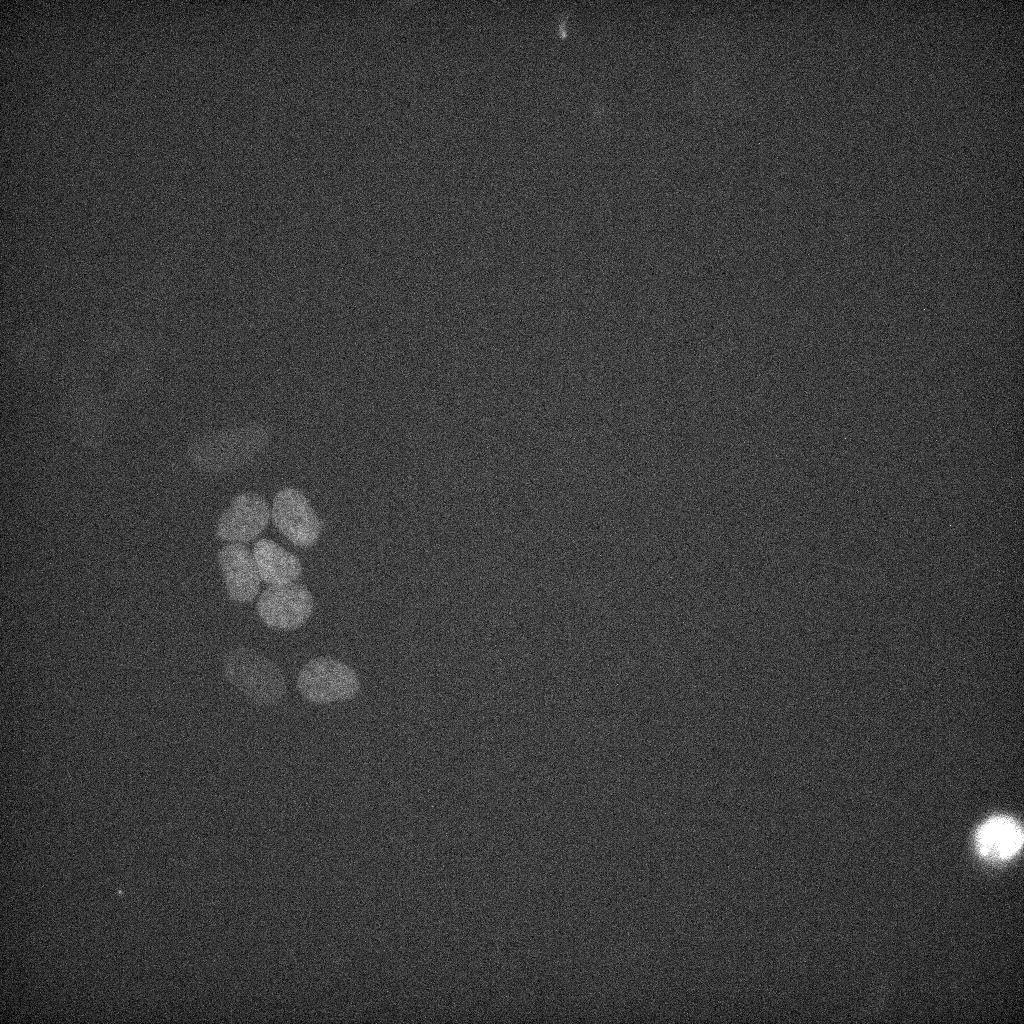

Supplement: Supplementary file 3 — Source Data [file 41467_2020_20757_MOESM3_ESM.zip › source_data/figure 1b-c images/HP1a_woDox_images/Position029_t102_ch01.tif]

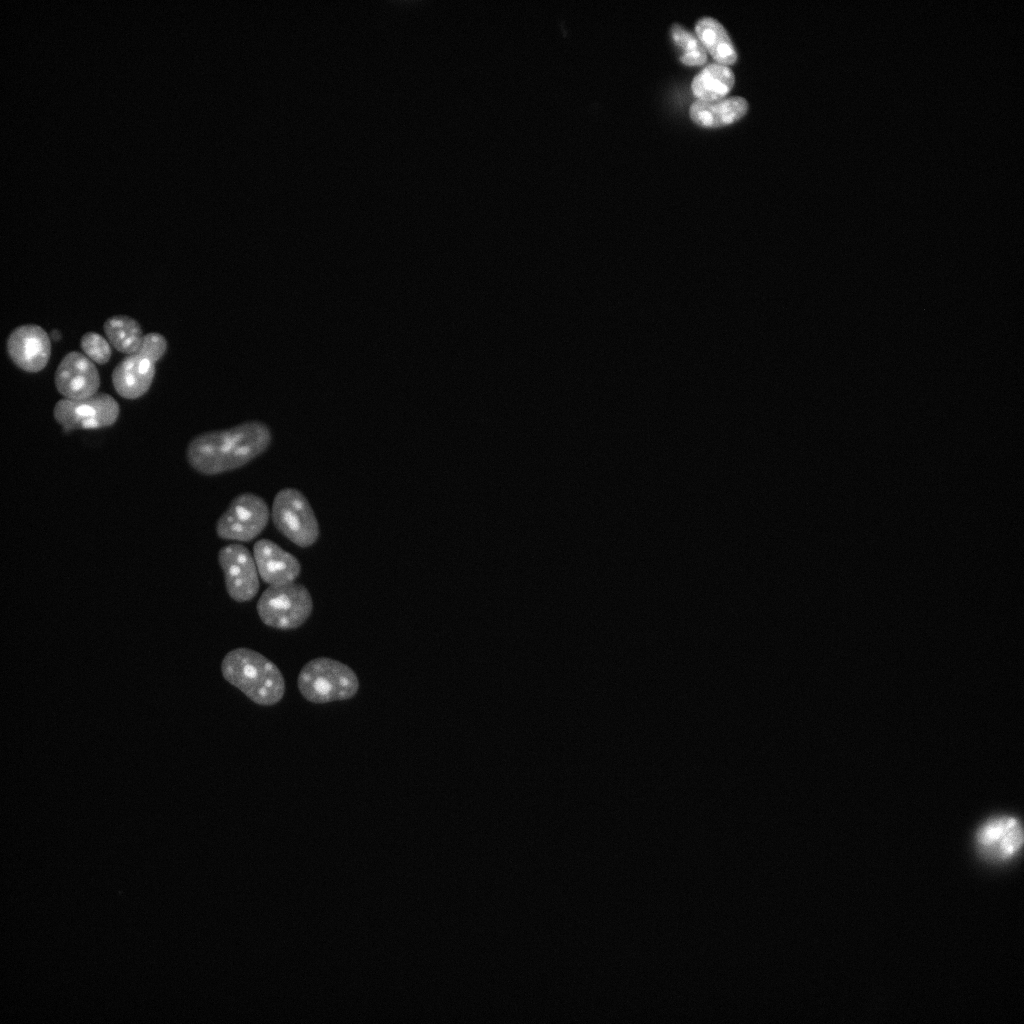

Supplement: Supplementary file 3 — Source Data [file 41467_2020_20757_MOESM3_ESM.zip › source_data/figure 1b-c images/HP1a_woDox_images/Position029_t102_ch02.tif]

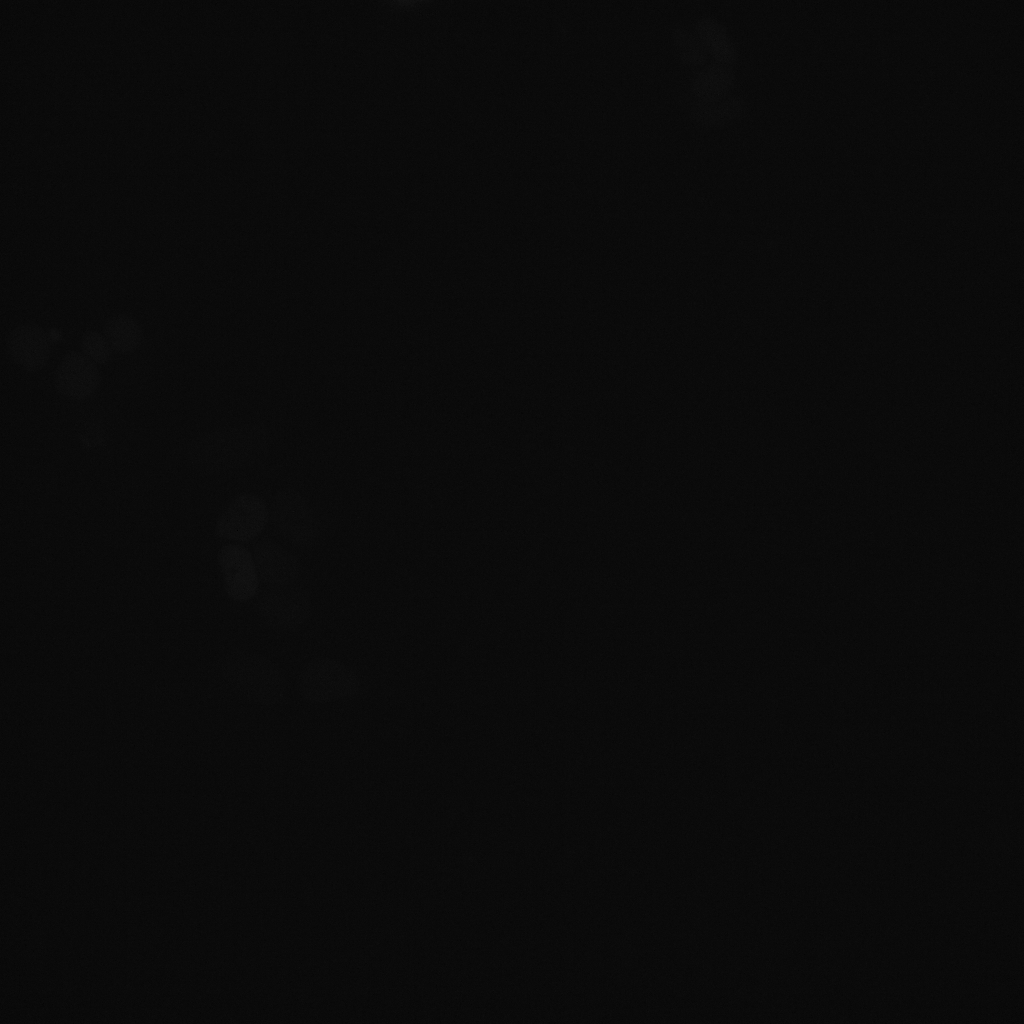

Supplement: Supplementary file 3 — Source Data [file 41467_2020_20757_MOESM3_ESM.zip › source_data/figure 1b-c images/HP1a_woDox_images/Position029_t102_ch03.tif]

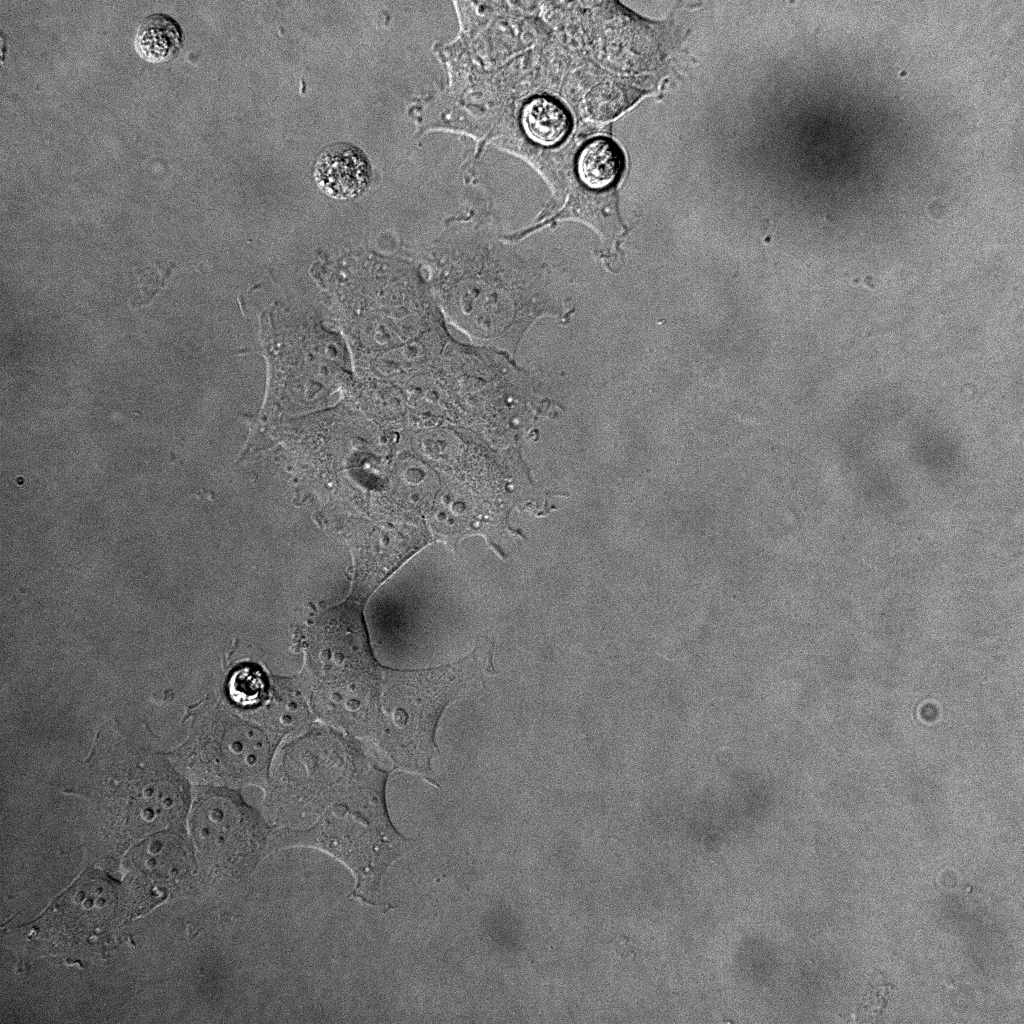

Supplement: Supplementary file 3 — Source Data [file 41467_2020_20757_MOESM3_ESM.zip › source_data/figure 1b-c images/HP1a_woDox_images/Position029_t151_ch00.tif]

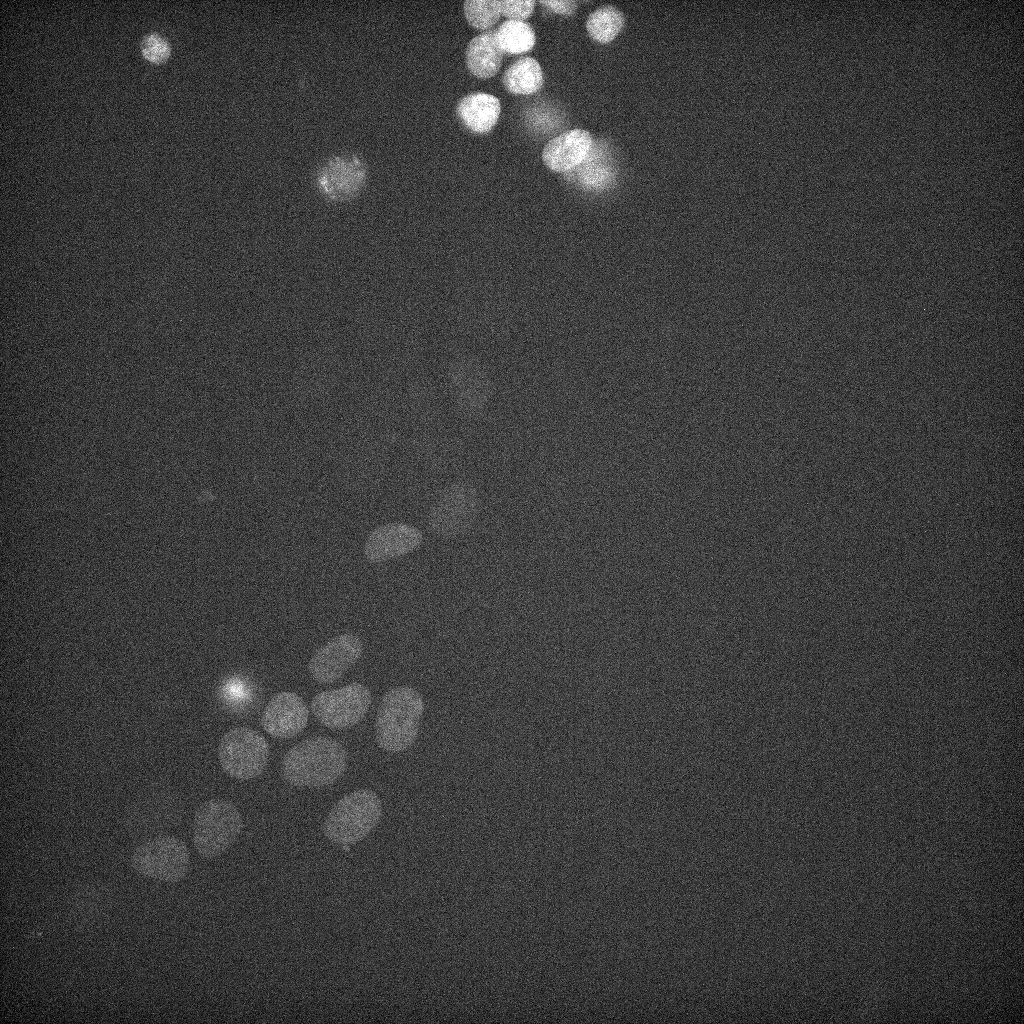

Supplement: Supplementary file 3 — Source Data [file 41467_2020_20757_MOESM3_ESM.zip › source_data/figure 1b-c images/HP1a_woDox_images/Position029_t151_ch01.tif]

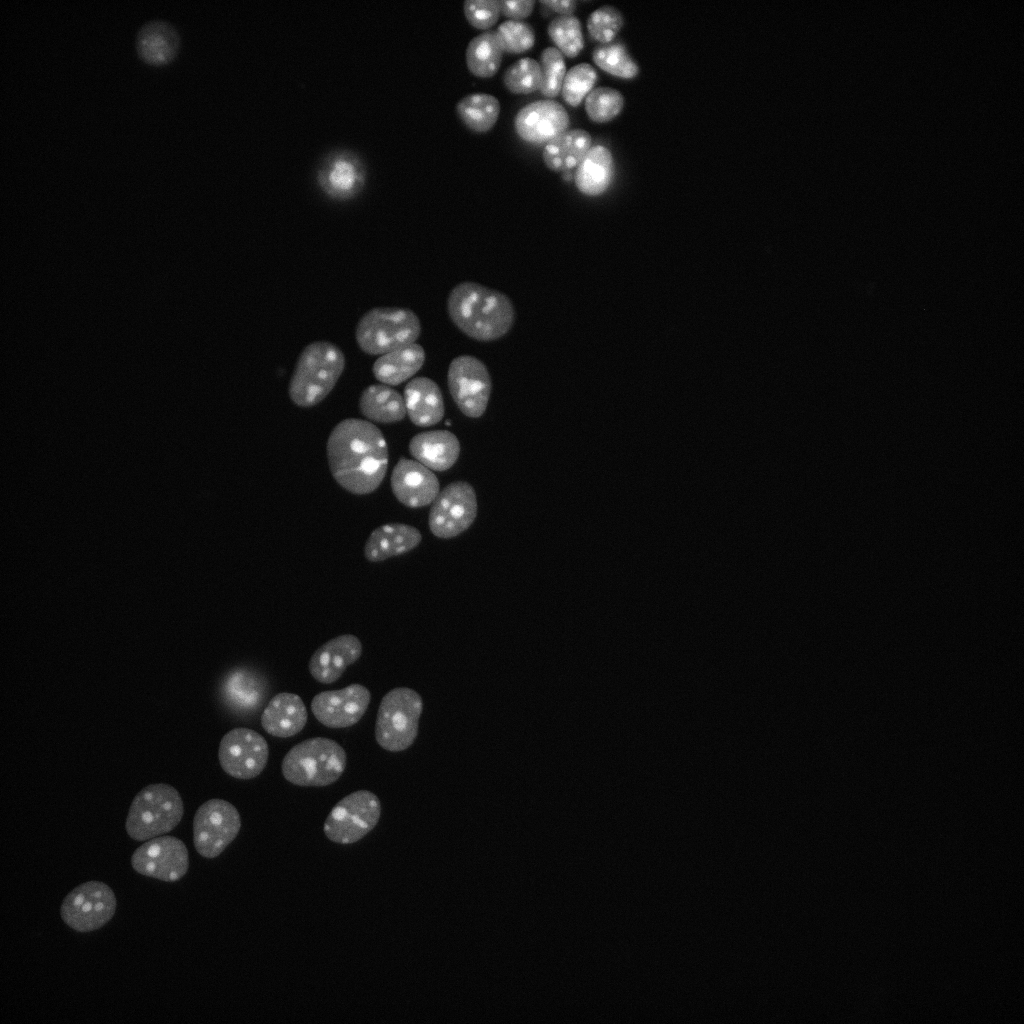

Supplement: Supplementary file 3 — Source Data [file 41467_2020_20757_MOESM3_ESM.zip › source_data/figure 1b-c images/HP1a_woDox_images/Position029_t151_ch02.tif]

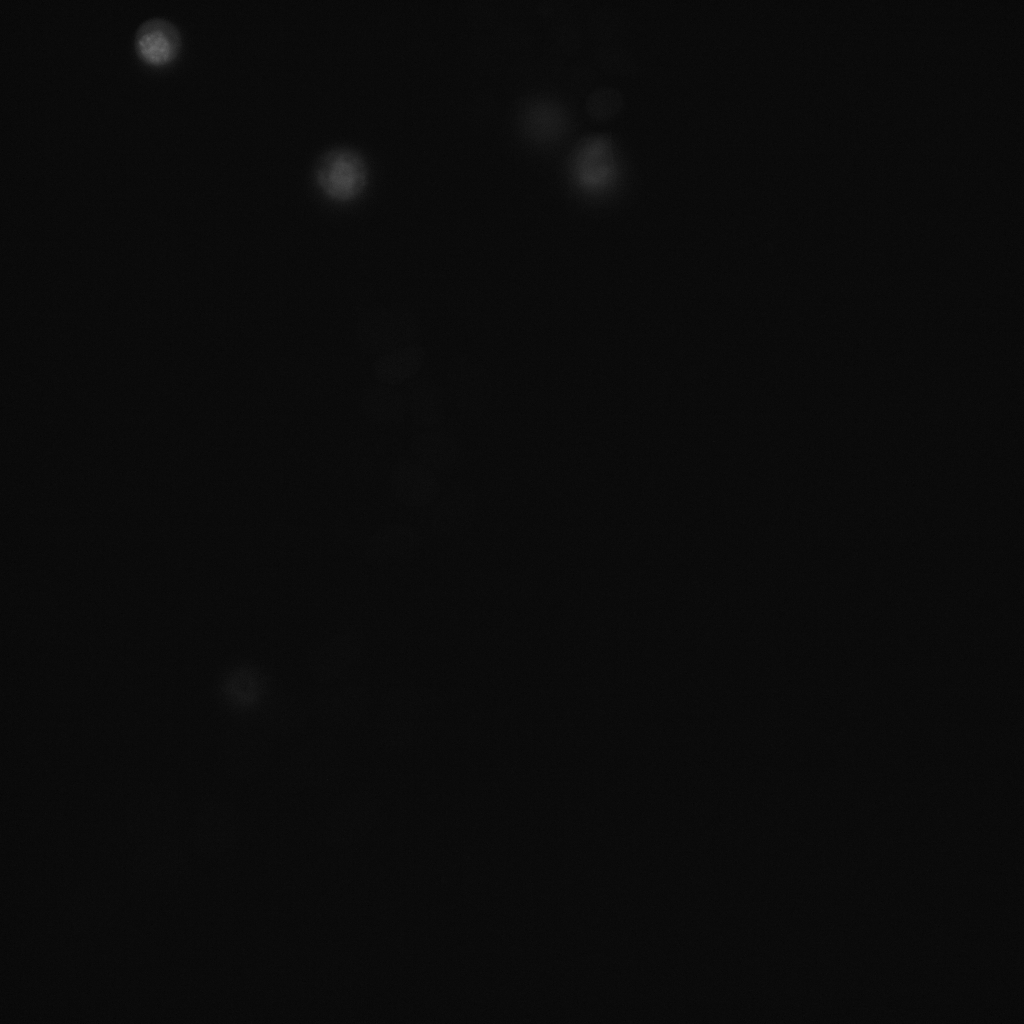

Supplement: Supplementary file 3 — Source Data [file 41467_2020_20757_MOESM3_ESM.zip › source_data/figure 1b-c images/HP1a_woDox_images/Position029_t151_ch03.tif]

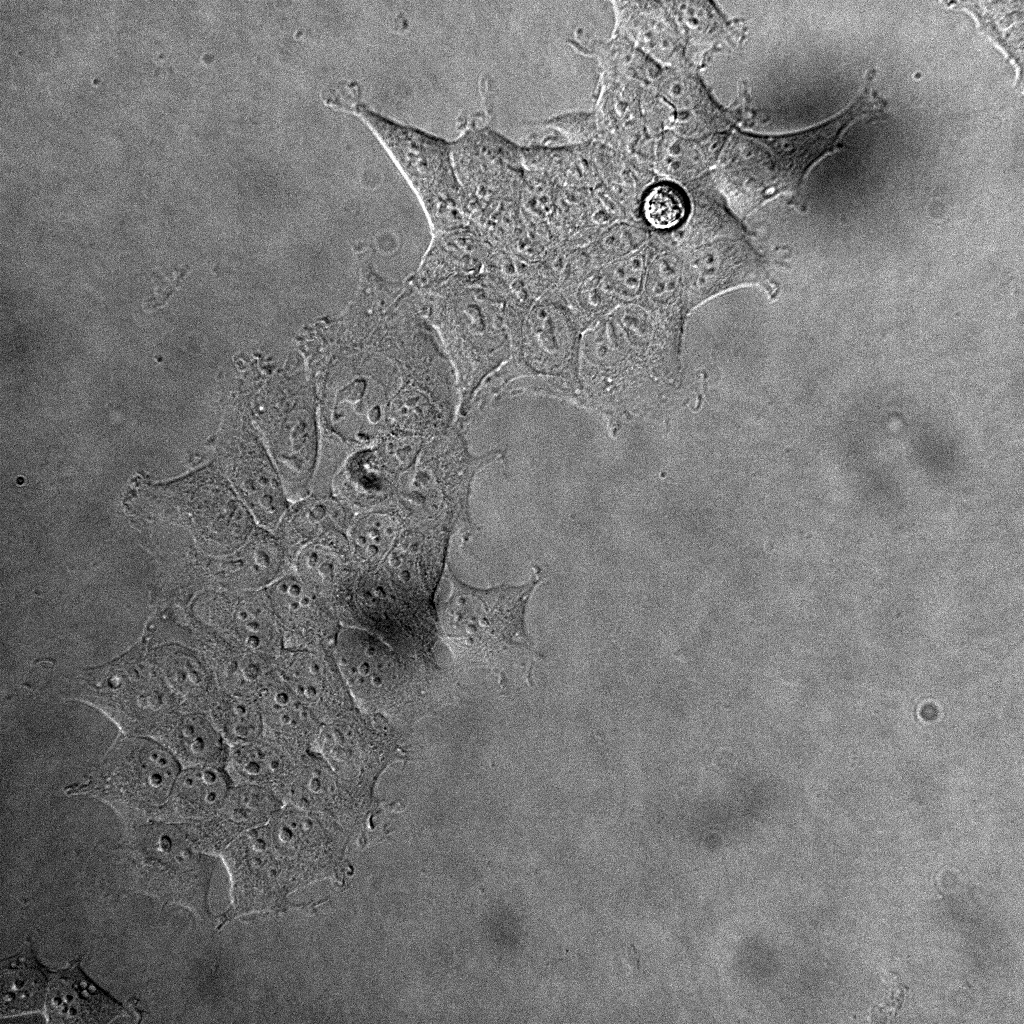

Supplement: Supplementary file 3 — Source Data [file 41467_2020_20757_MOESM3_ESM.zip › source_data/figure 1b-c images/HP1a_woDox_images/Position029_t201_ch00.tif]

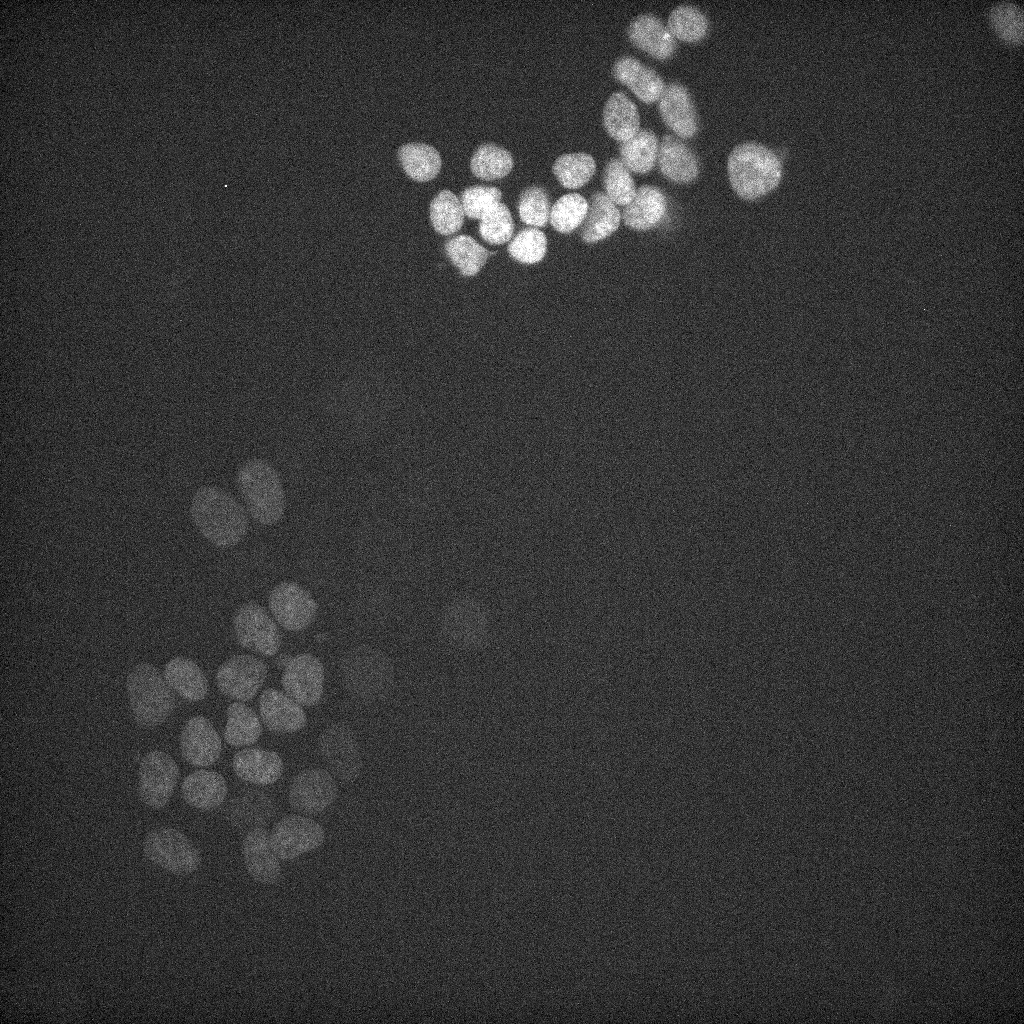

Supplement: Supplementary file 3 — Source Data [file 41467_2020_20757_MOESM3_ESM.zip › source_data/figure 1b-c images/HP1a_woDox_images/Position029_t201_ch01.tif]

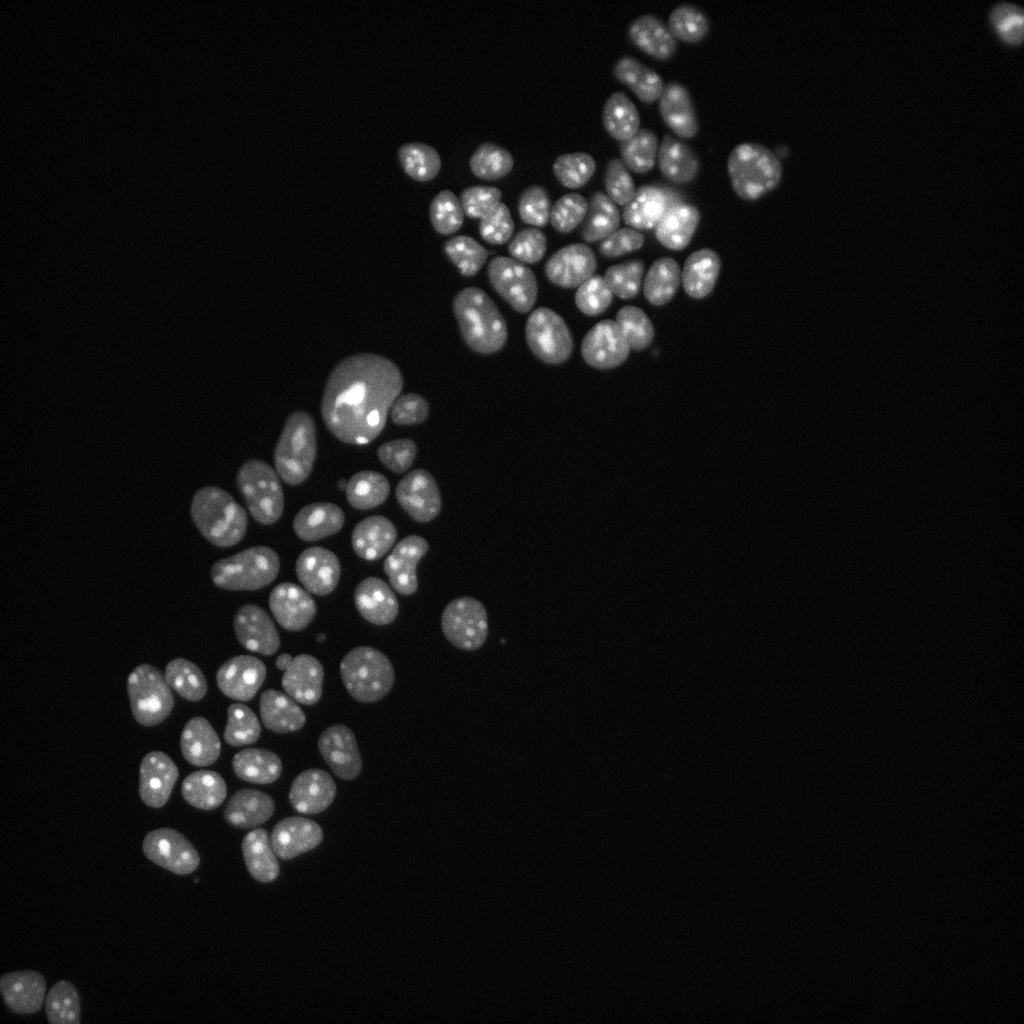

Supplement: Supplementary file 3 — Source Data [file 41467_2020_20757_MOESM3_ESM.zip › source_data/figure 1b-c images/HP1a_woDox_images/Position029_t201_ch02.tif]

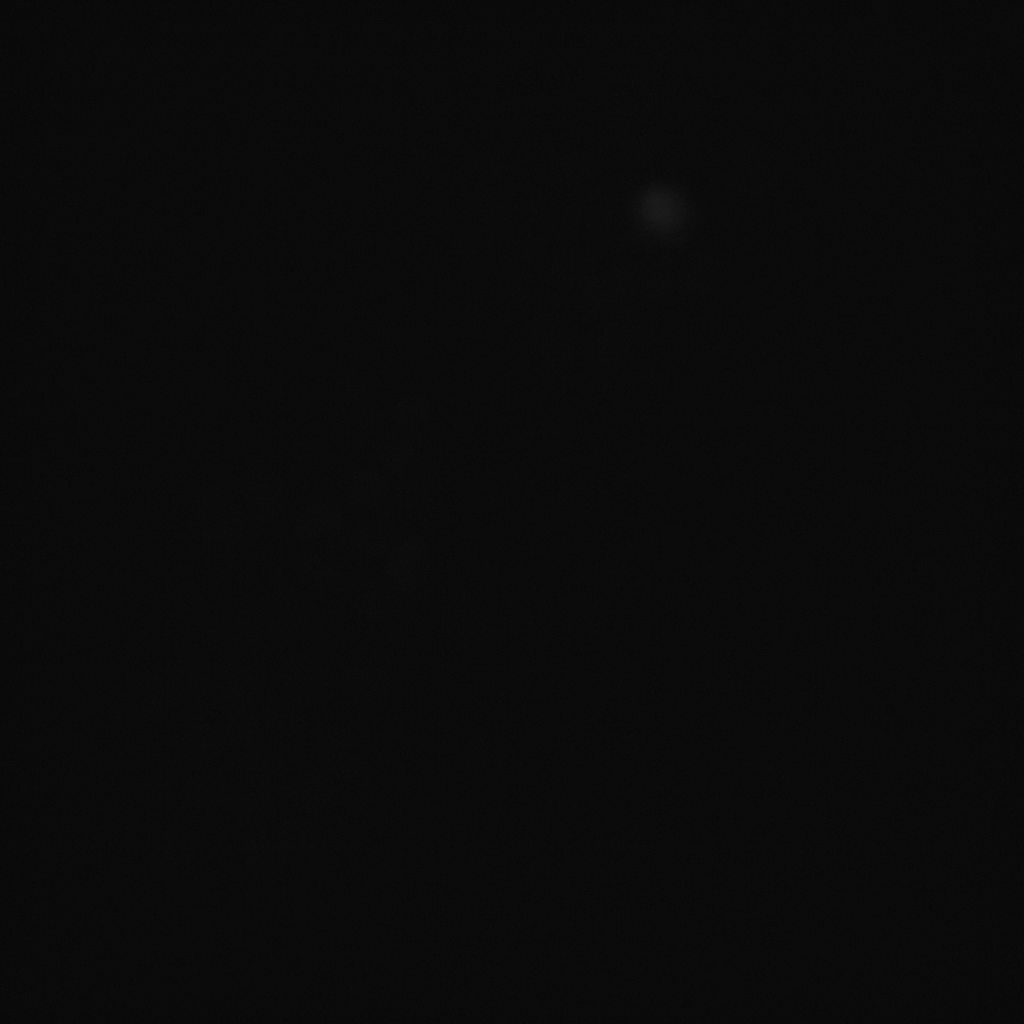

Supplement: Supplementary file 3 — Source Data [file 41467_2020_20757_MOESM3_ESM.zip › source_data/figure 1b-c images/HP1a_woDox_images/Position029_t201_ch03.tif]

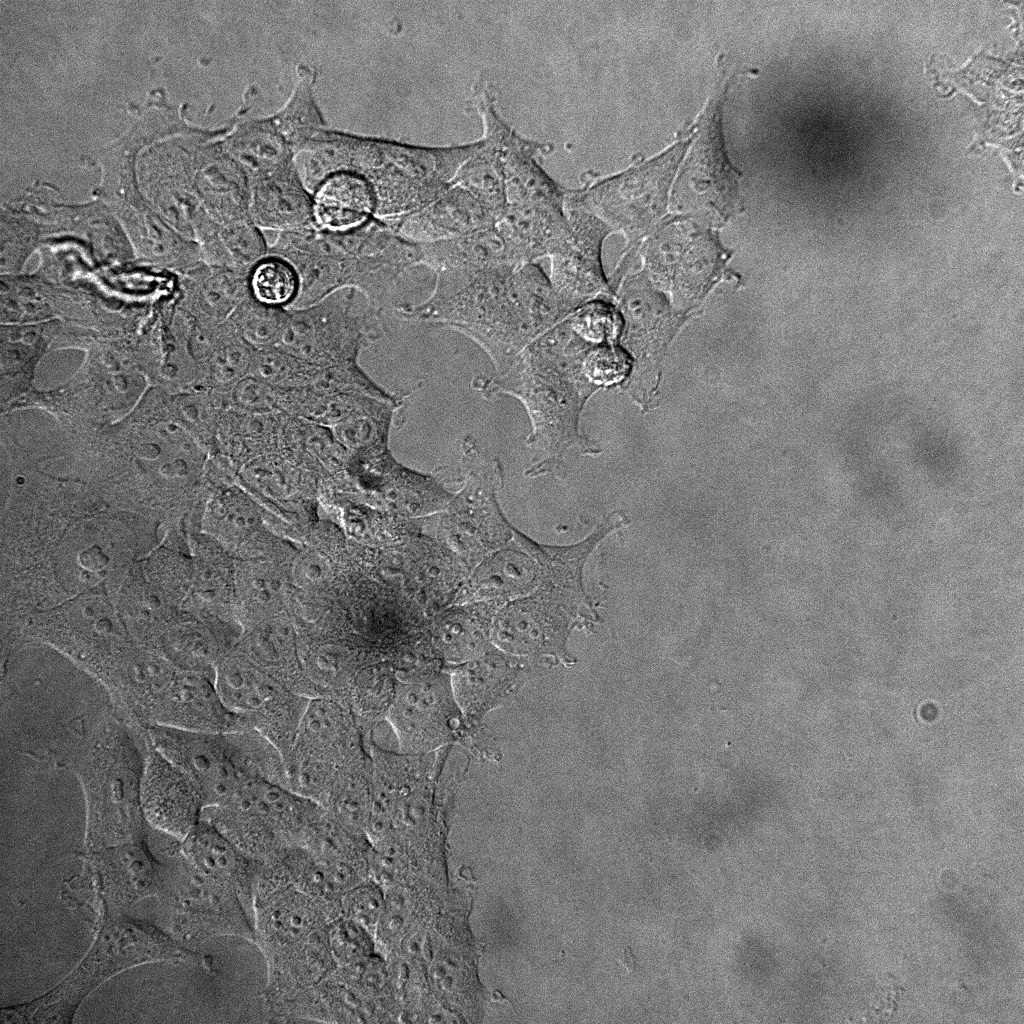

Supplement: Supplementary file 3 — Source Data [file 41467_2020_20757_MOESM3_ESM.zip › source_data/figure 1b-c images/HP1a_woDox_images/Position029_t251_ch00.tif]

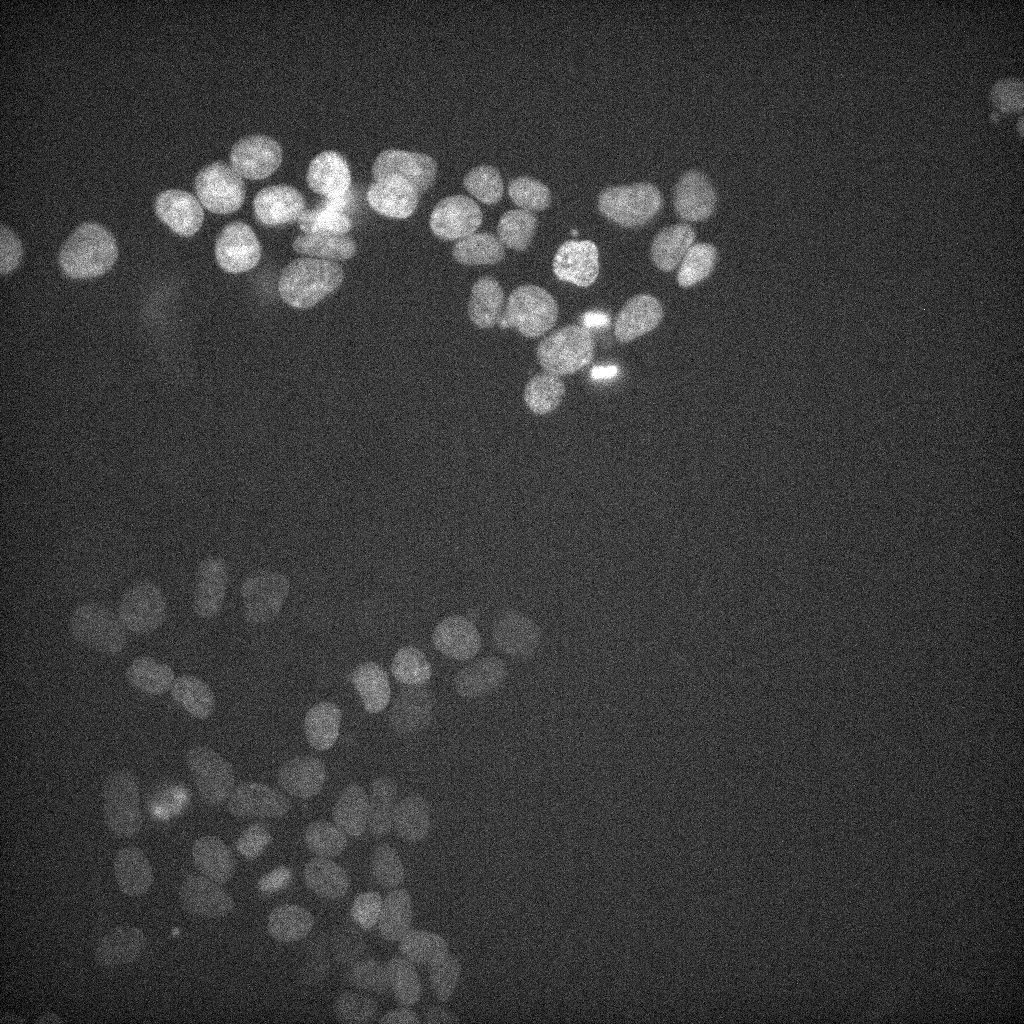

Supplement: Supplementary file 3 — Source Data [file 41467_2020_20757_MOESM3_ESM.zip › source_data/figure 1b-c images/HP1a_woDox_images/Position029_t251_ch01.tif]

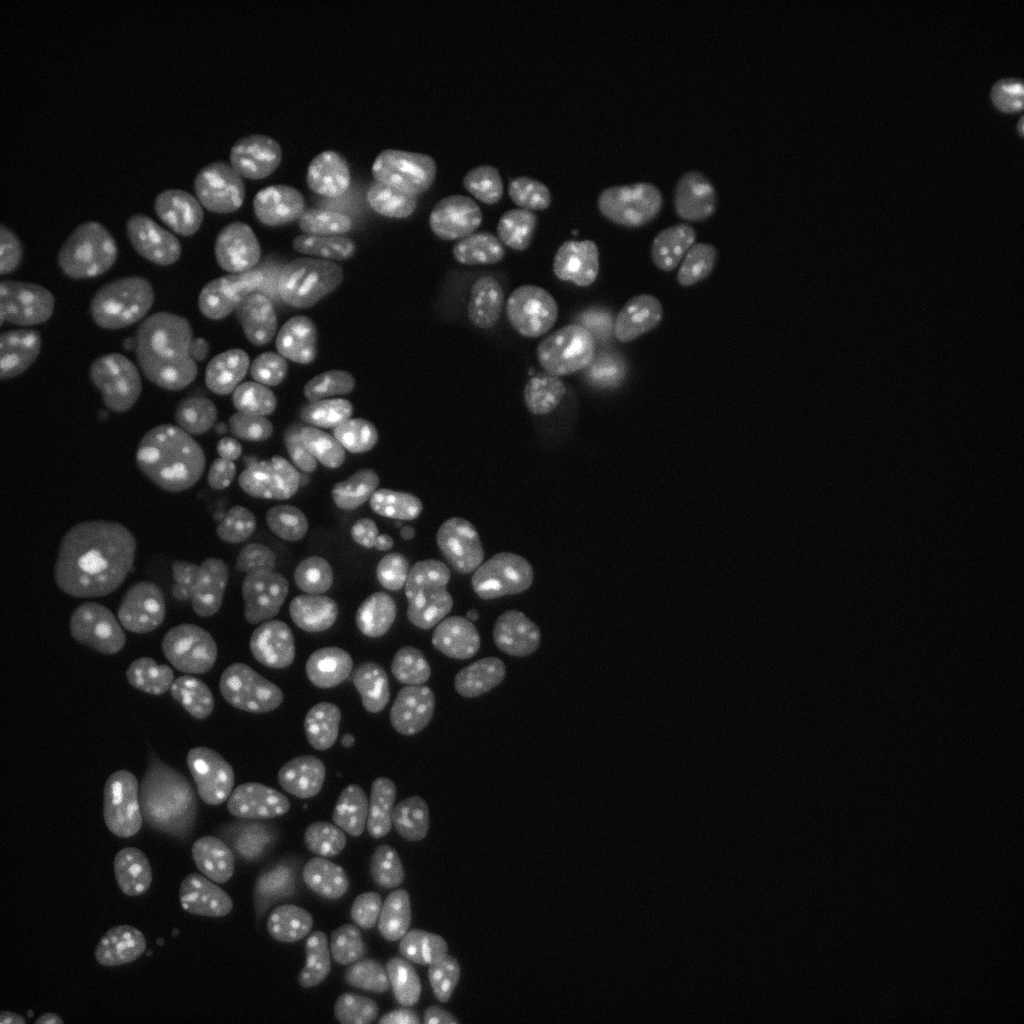

Supplement: Supplementary file 3 — Source Data [file 41467_2020_20757_MOESM3_ESM.zip › source_data/figure 1b-c images/HP1a_woDox_images/Position029_t251_ch02.tif]

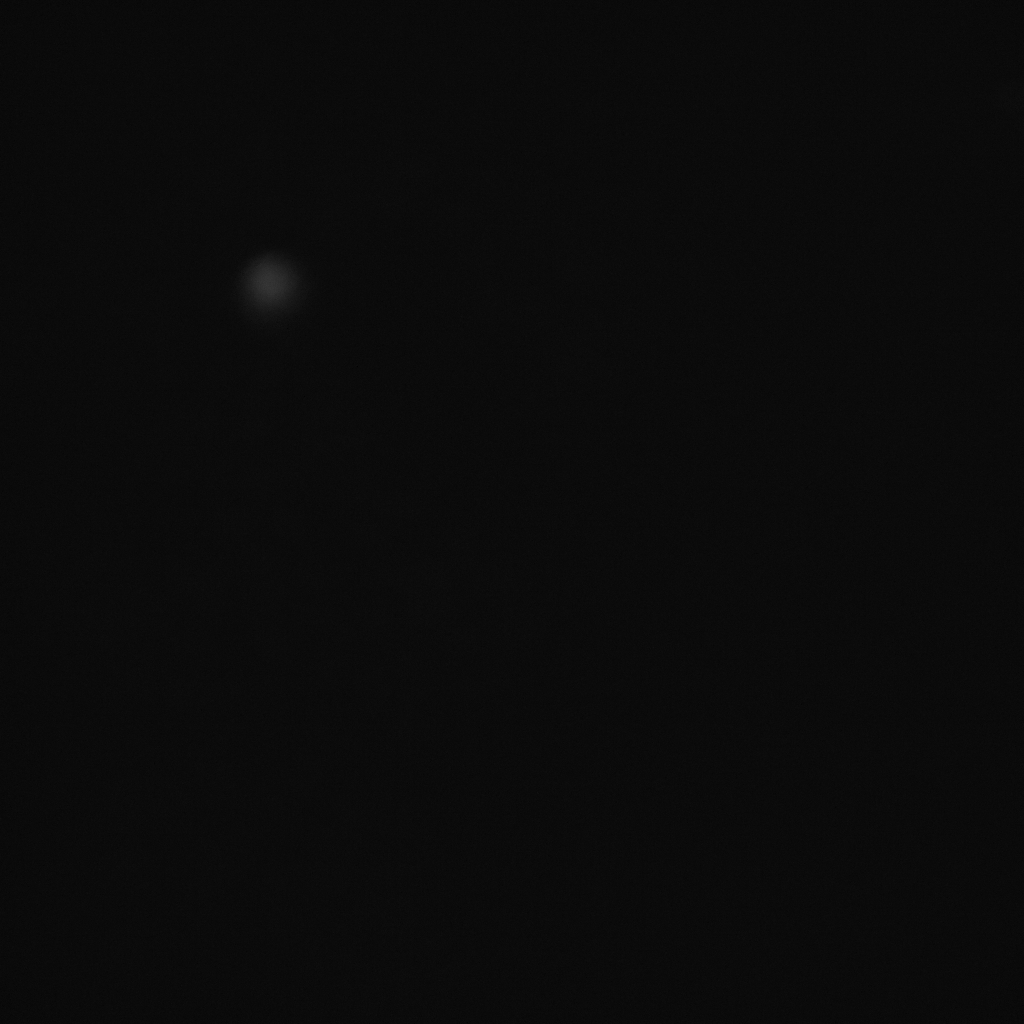

Supplement: Supplementary file 3 — Source Data [file 41467_2020_20757_MOESM3_ESM.zip › source_data/figure 1b-c images/HP1a_woDox_images/Position029_t251_ch03.tif]

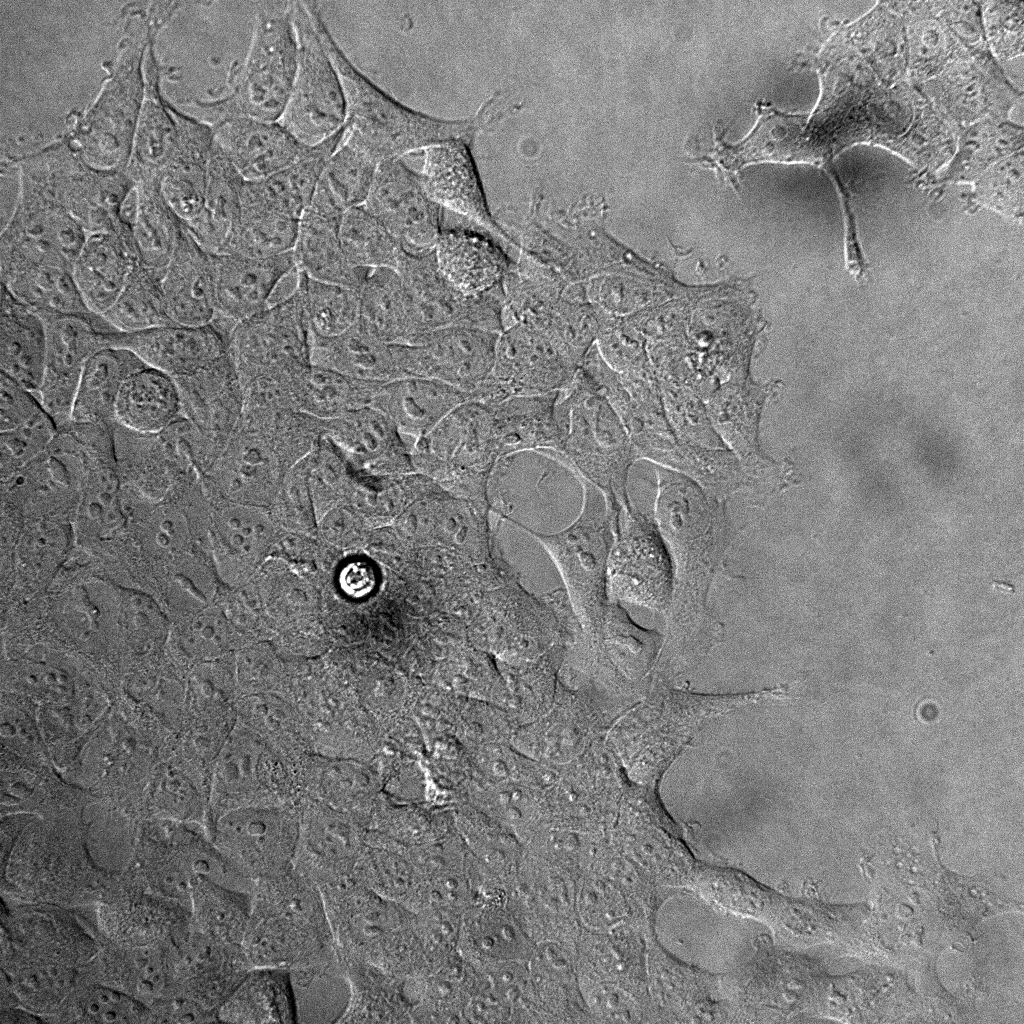

Supplement: Supplementary file 3 — Source Data [file 41467_2020_20757_MOESM3_ESM.zip › source_data/figure 1b-c images/HP1a_woDox_images/Position029_t301_ch00.tif]

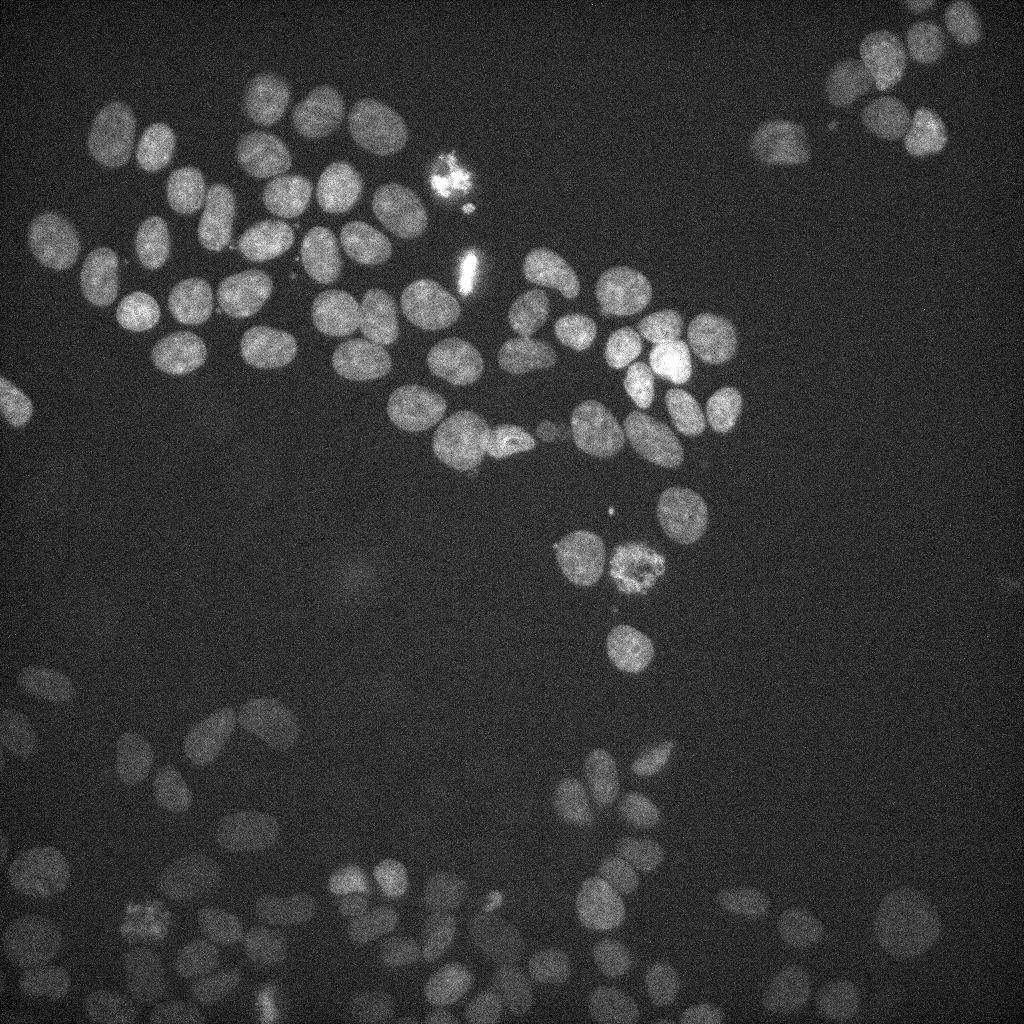

Supplement: Supplementary file 3 — Source Data [file 41467_2020_20757_MOESM3_ESM.zip › source_data/figure 1b-c images/HP1a_woDox_images/Position029_t301_ch01.tif]

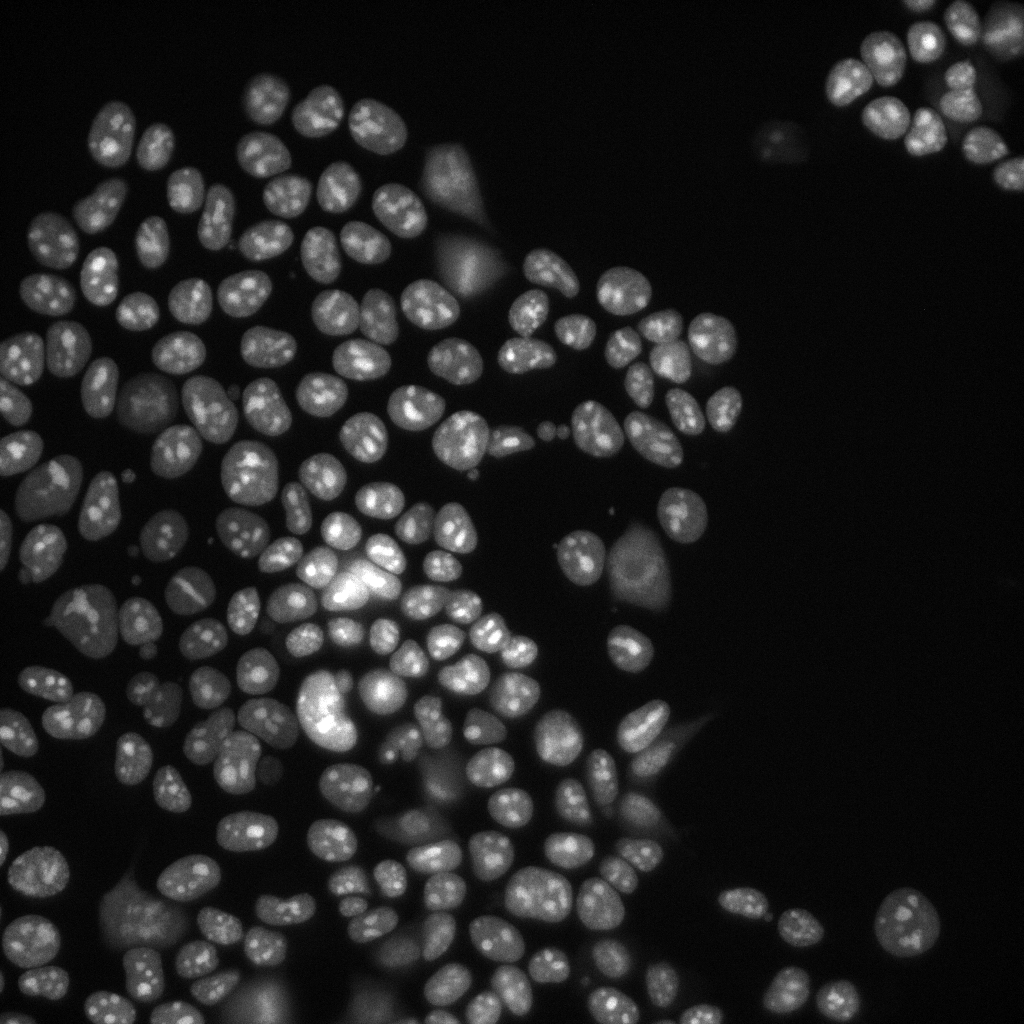

Supplement: Supplementary file 3 — Source Data [file 41467_2020_20757_MOESM3_ESM.zip › source_data/figure 1b-c images/HP1a_woDox_images/Position029_t301_ch02.tif]

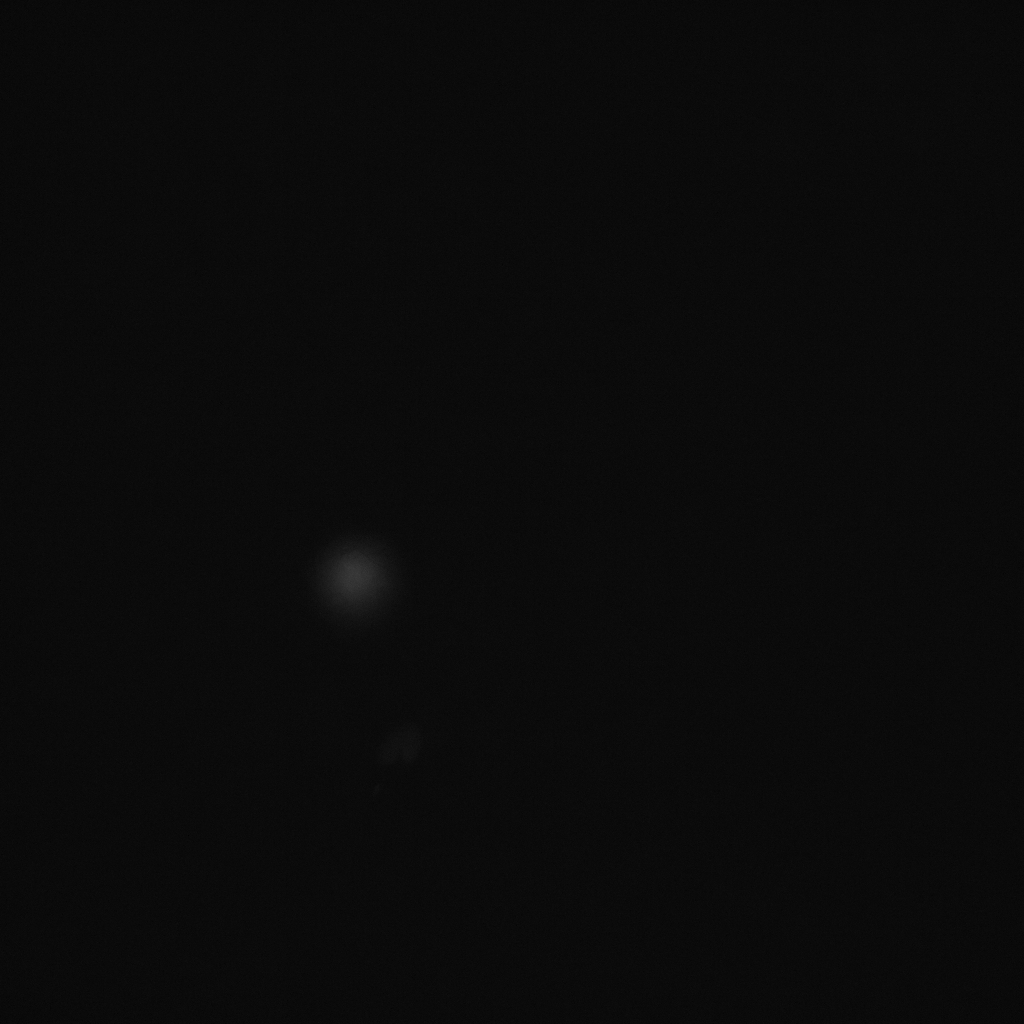

Supplement: Supplementary file 3 — Source Data [file 41467_2020_20757_MOESM3_ESM.zip › source_data/figure 1b-c images/HP1a_woDox_images/Position029_t301_ch03.tif]

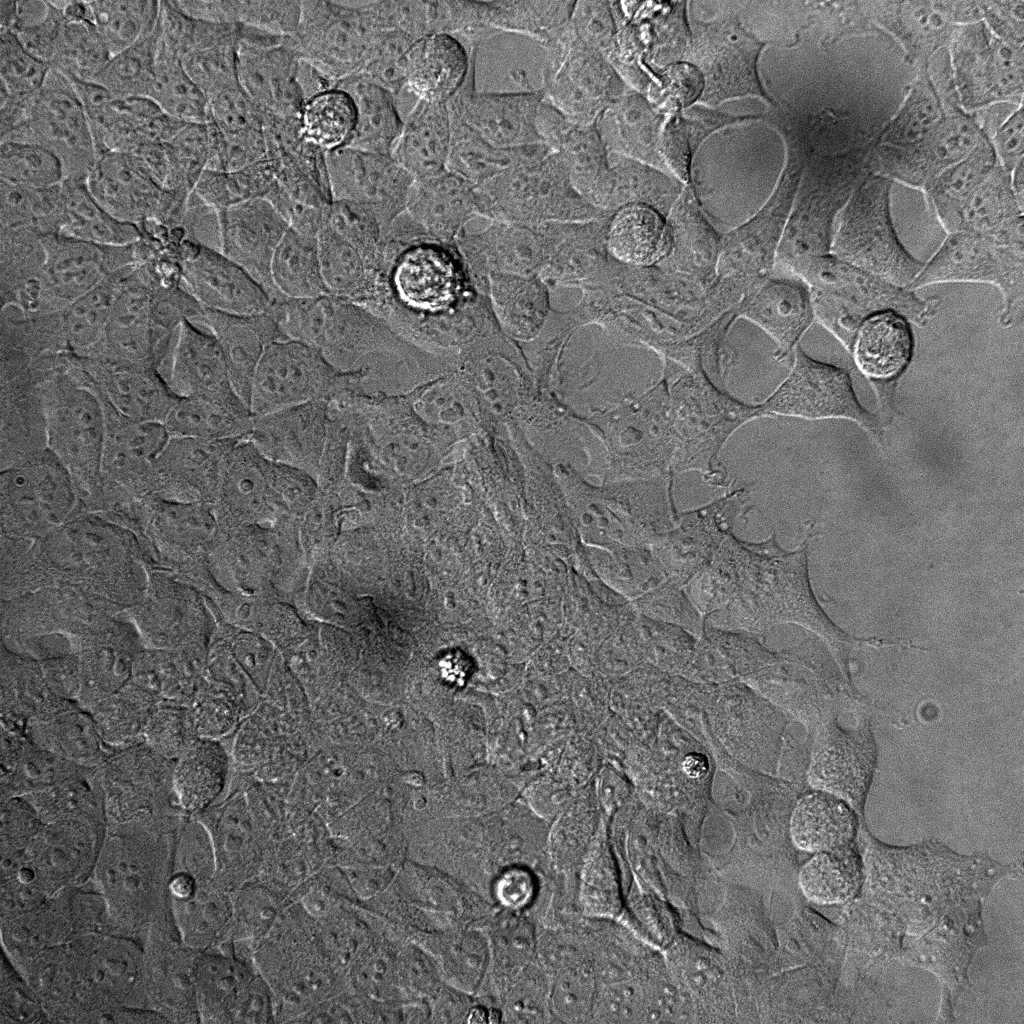

Supplement: Supplementary file 3 — Source Data [file 41467_2020_20757_MOESM3_ESM.zip › source_data/figure 1b-c images/HP1a_woDox_images/Position029_t352_ch00.tif]

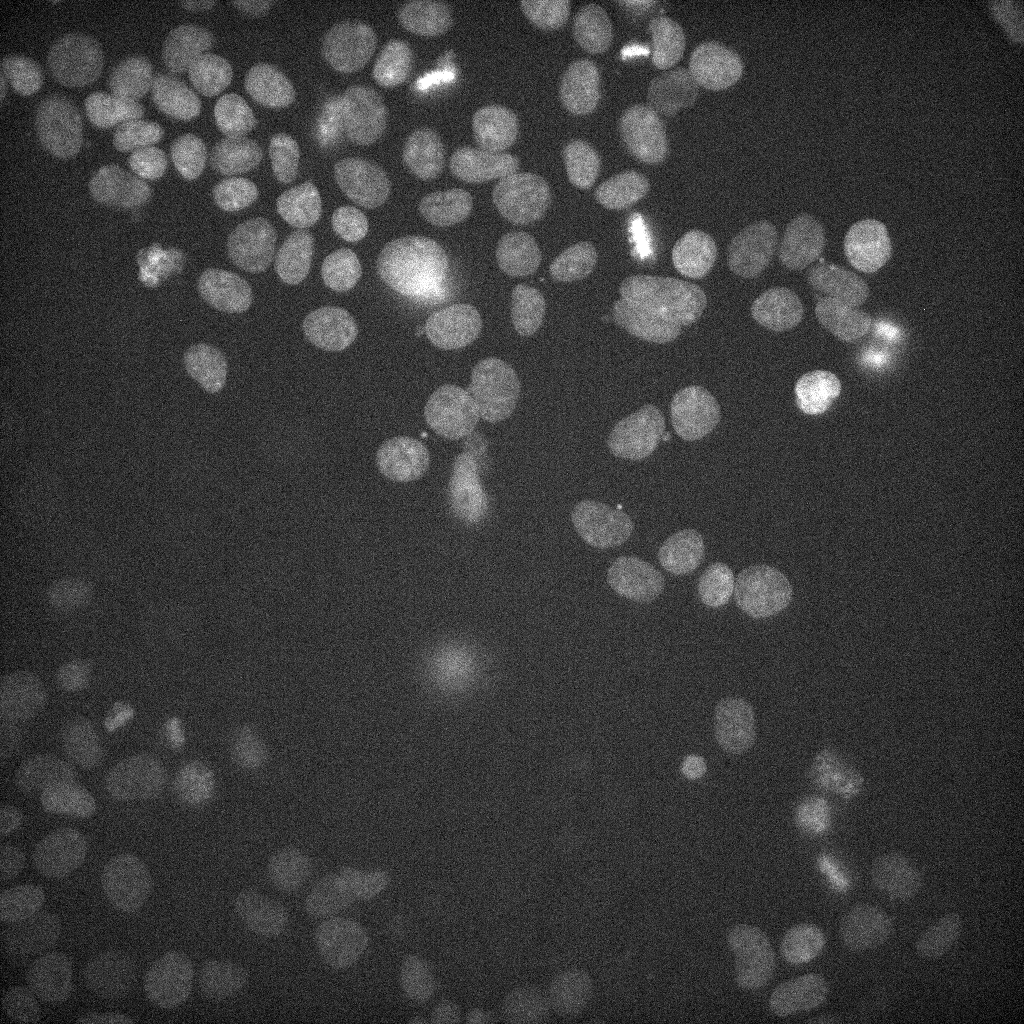

Supplement: Supplementary file 3 — Source Data [file 41467_2020_20757_MOESM3_ESM.zip › source_data/figure 1b-c images/HP1a_woDox_images/Position029_t352_ch01.tif]

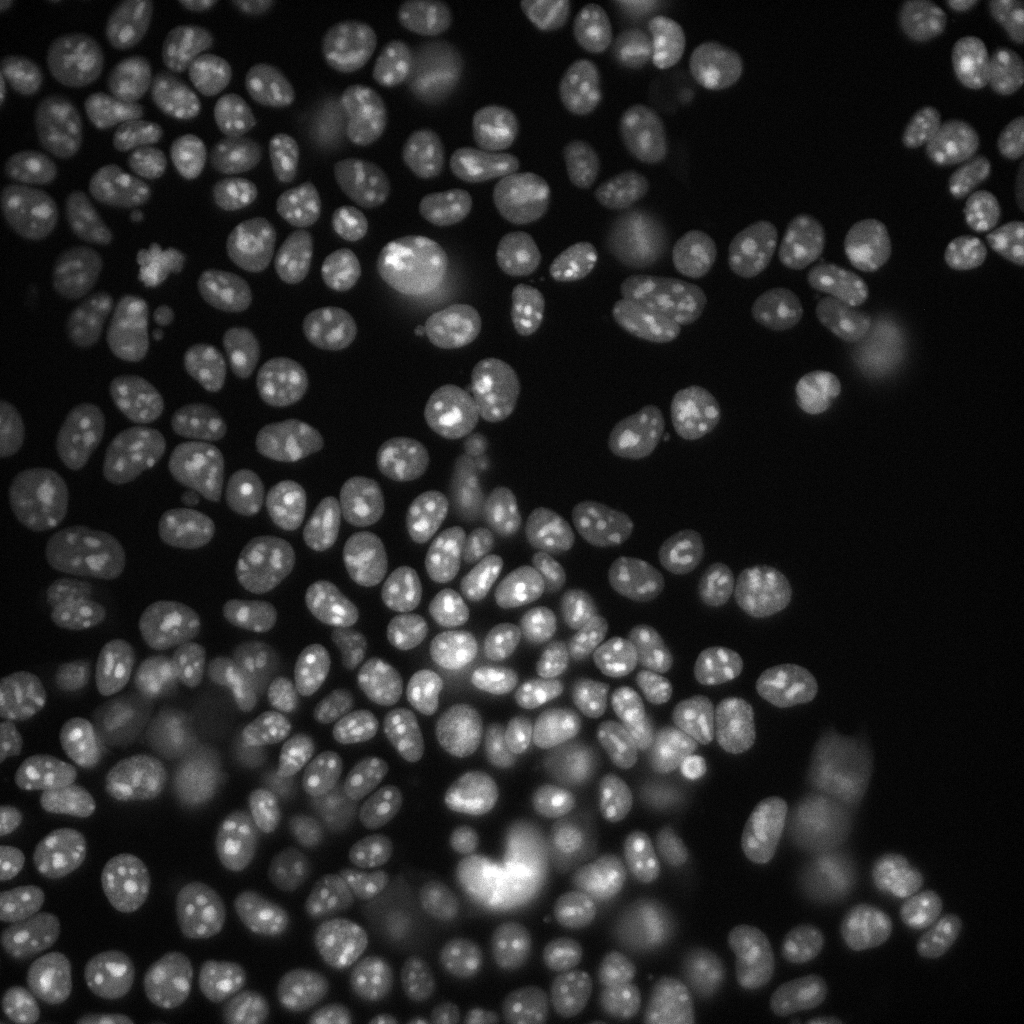

Supplement: Supplementary file 3 — Source Data [file 41467_2020_20757_MOESM3_ESM.zip › source_data/figure 1b-c images/HP1a_woDox_images/Position029_t352_ch02.tif]

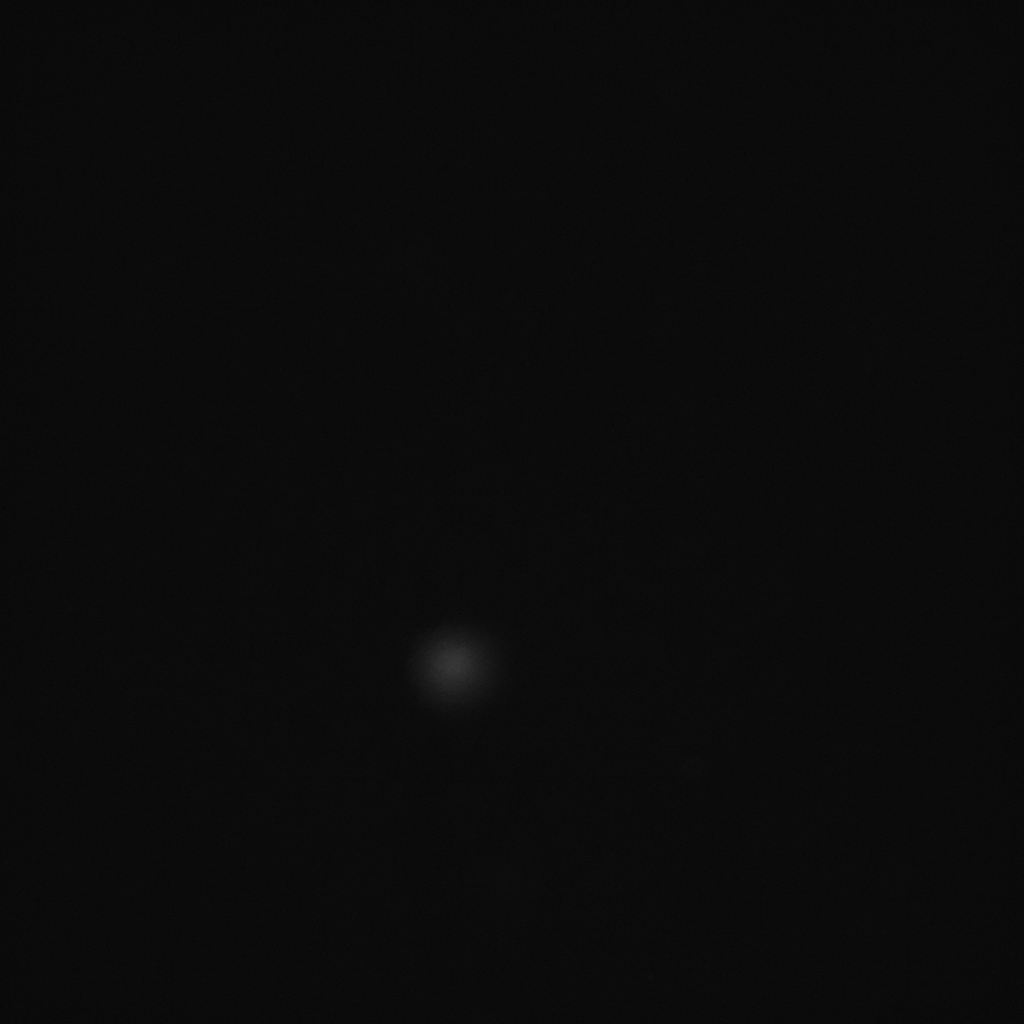

Supplement: Supplementary file 3 — Source Data [file 41467_2020_20757_MOESM3_ESM.zip › source_data/figure 1b-c images/HP1a_woDox_images/Position029_t352_ch03.tif]

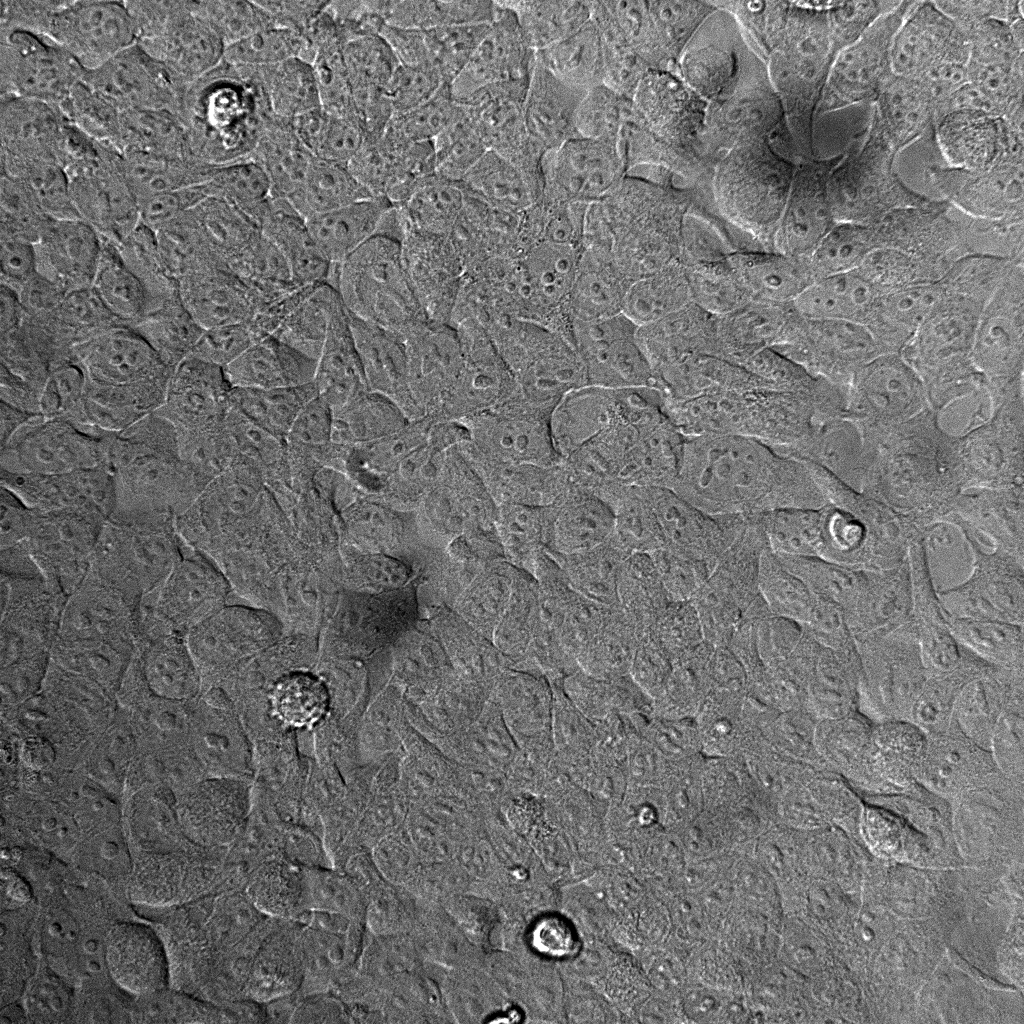

Supplement: Supplementary file 3 — Source Data [file 41467_2020_20757_MOESM3_ESM.zip › source_data/figure 1b-c images/HP1a_woDox_images/Position029_t405_ch00.tif]

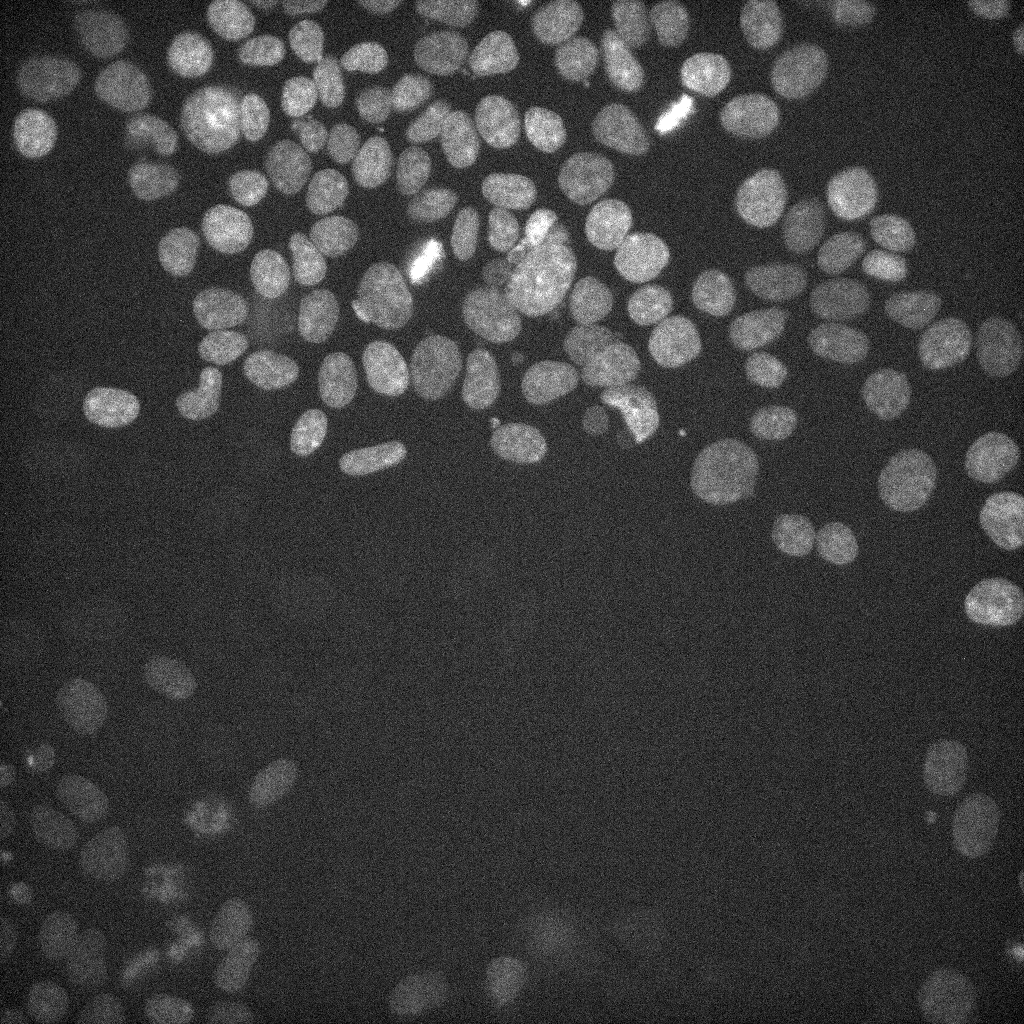

Supplement: Supplementary file 3 — Source Data [file 41467_2020_20757_MOESM3_ESM.zip › source_data/figure 1b-c images/HP1a_woDox_images/Position029_t405_ch01.tif]

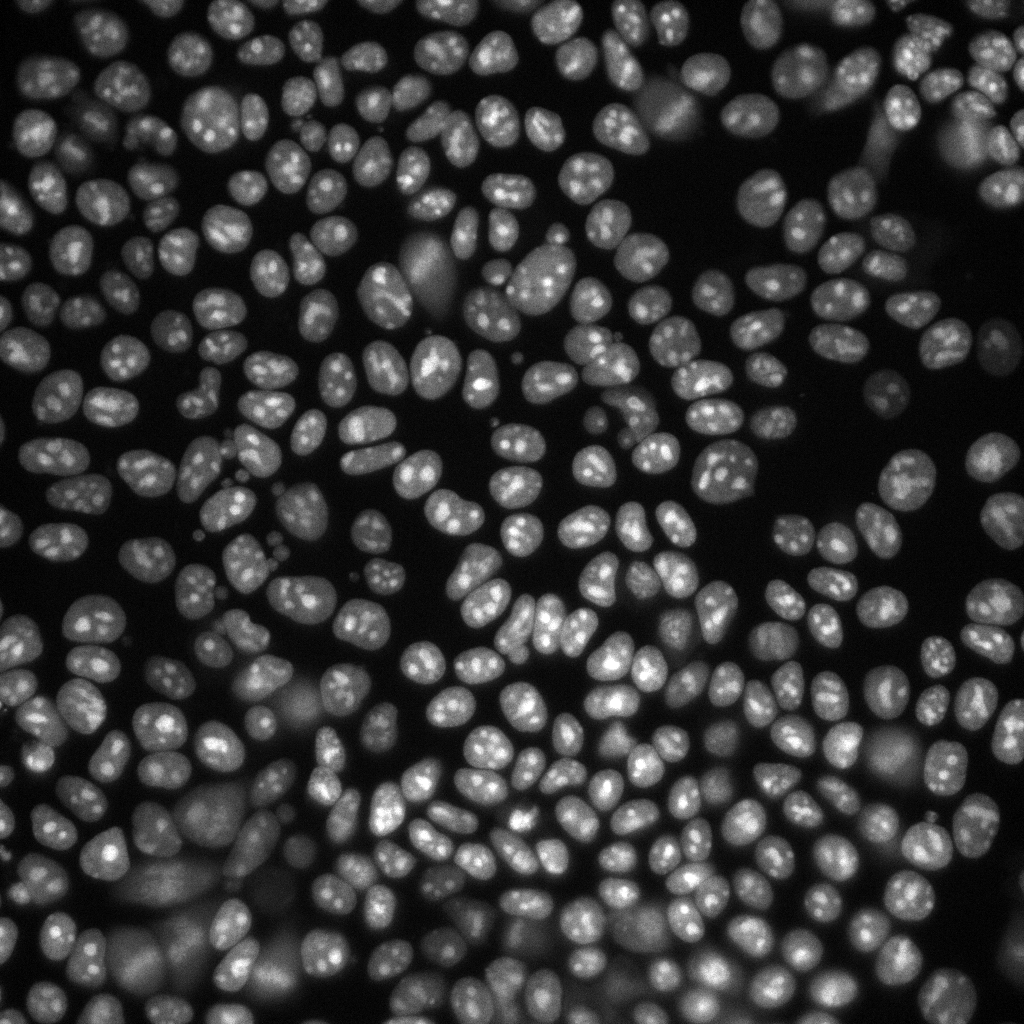

Supplement: Supplementary file 3 — Source Data [file 41467_2020_20757_MOESM3_ESM.zip › source_data/figure 1b-c images/HP1a_woDox_images/Position029_t405_ch02.tif]

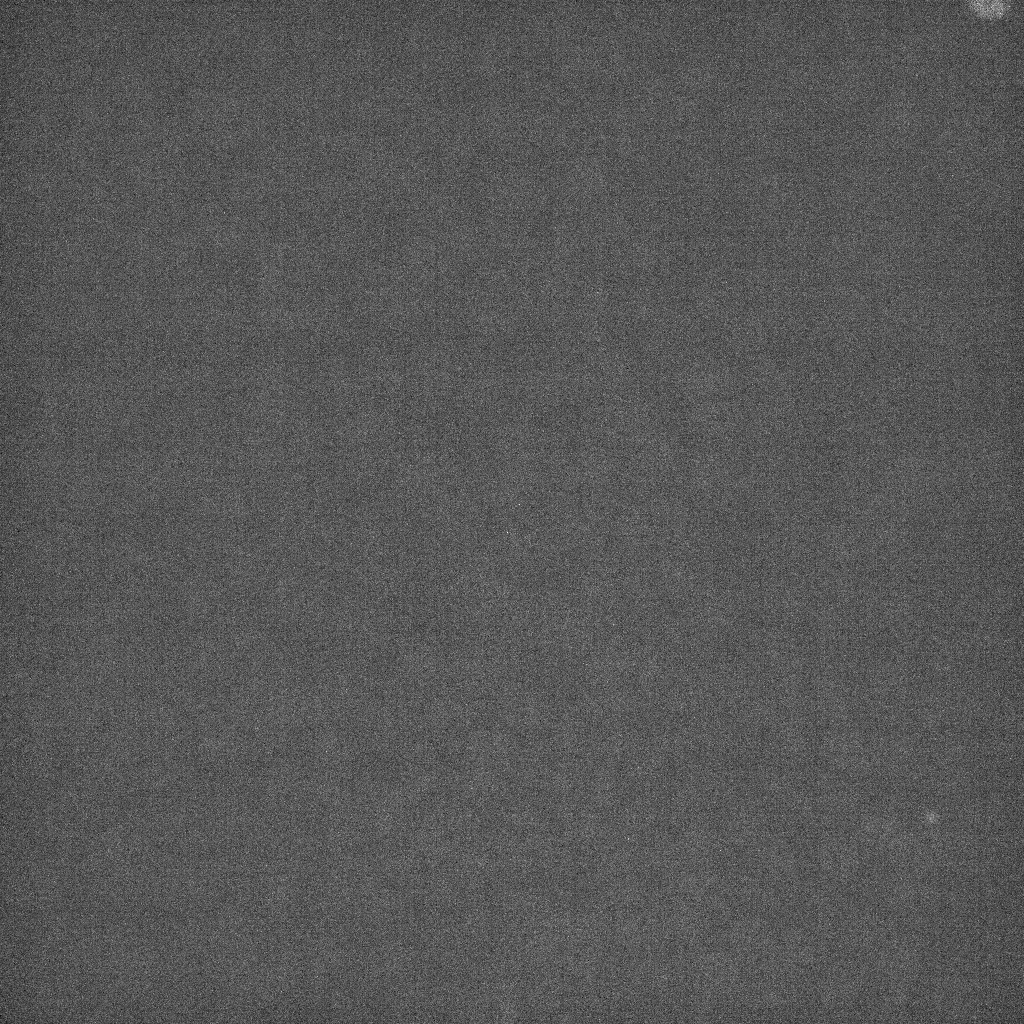

Supplement: Supplementary file 3 — Source Data [file 41467_2020_20757_MOESM3_ESM.zip › source_data/figure 1b-c images/HP1a_woDox_images/Position029_t405_ch03.tif]

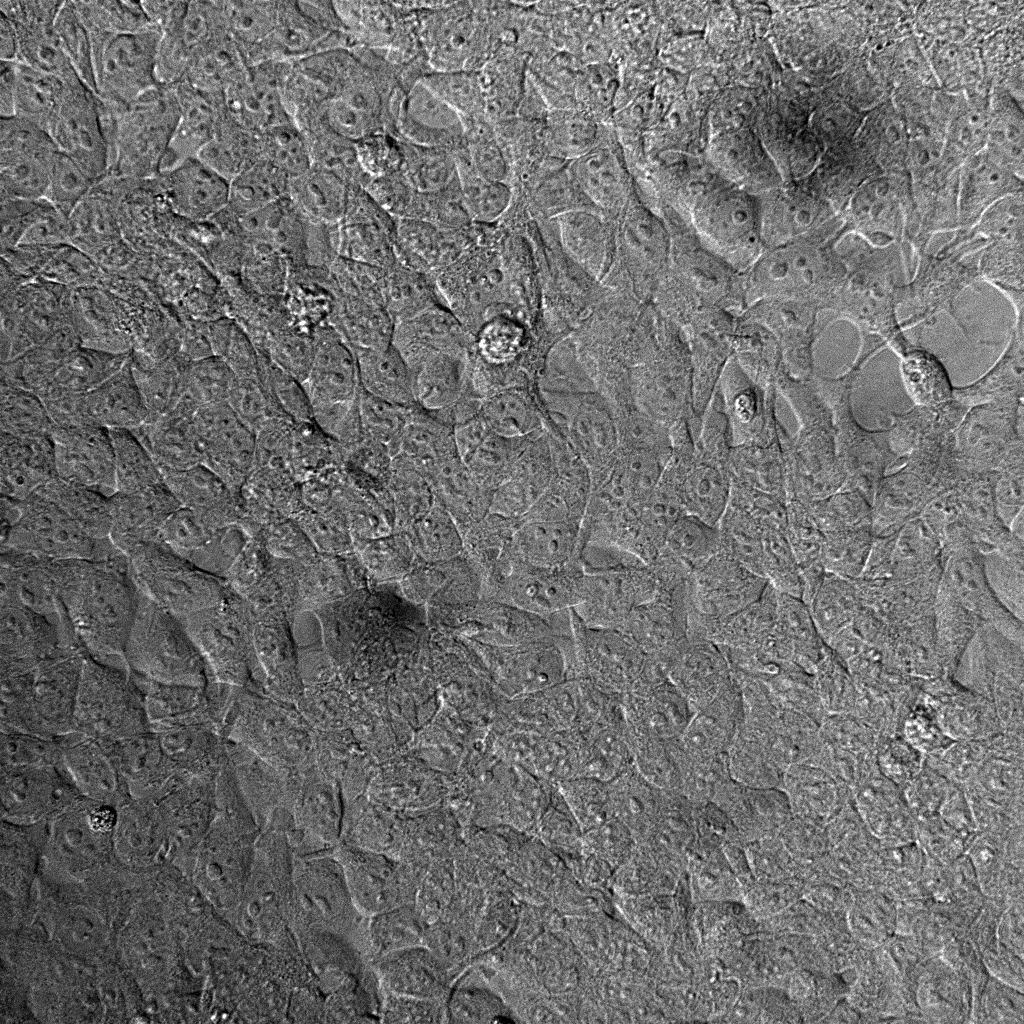

Supplement: Supplementary file 3 — Source Data [file 41467_2020_20757_MOESM3_ESM.zip › source_data/figure 1b-c images/HP1a_woDox_images/Position029_t454_ch00.tif]

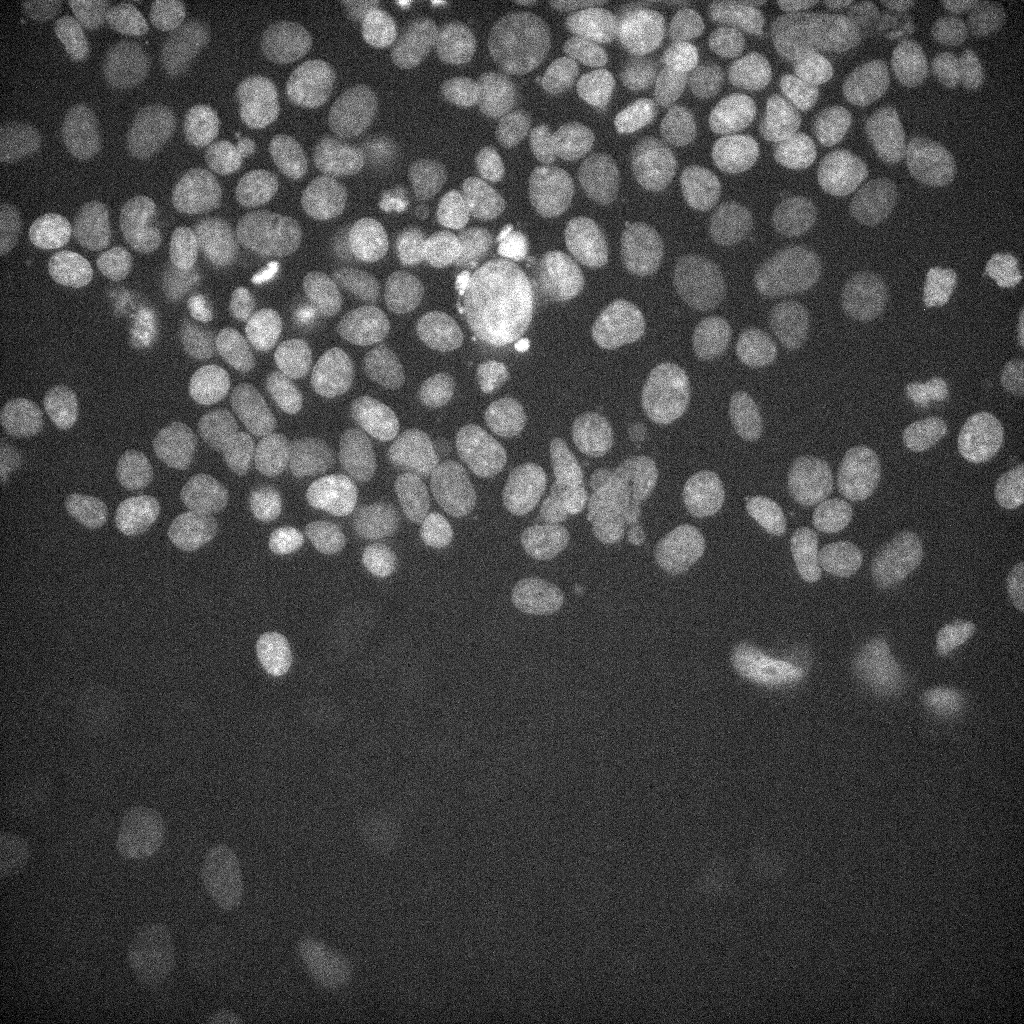

Supplement: Supplementary file 3 — Source Data [file 41467_2020_20757_MOESM3_ESM.zip › source_data/figure 1b-c images/HP1a_woDox_images/Position029_t454_ch01.tif]

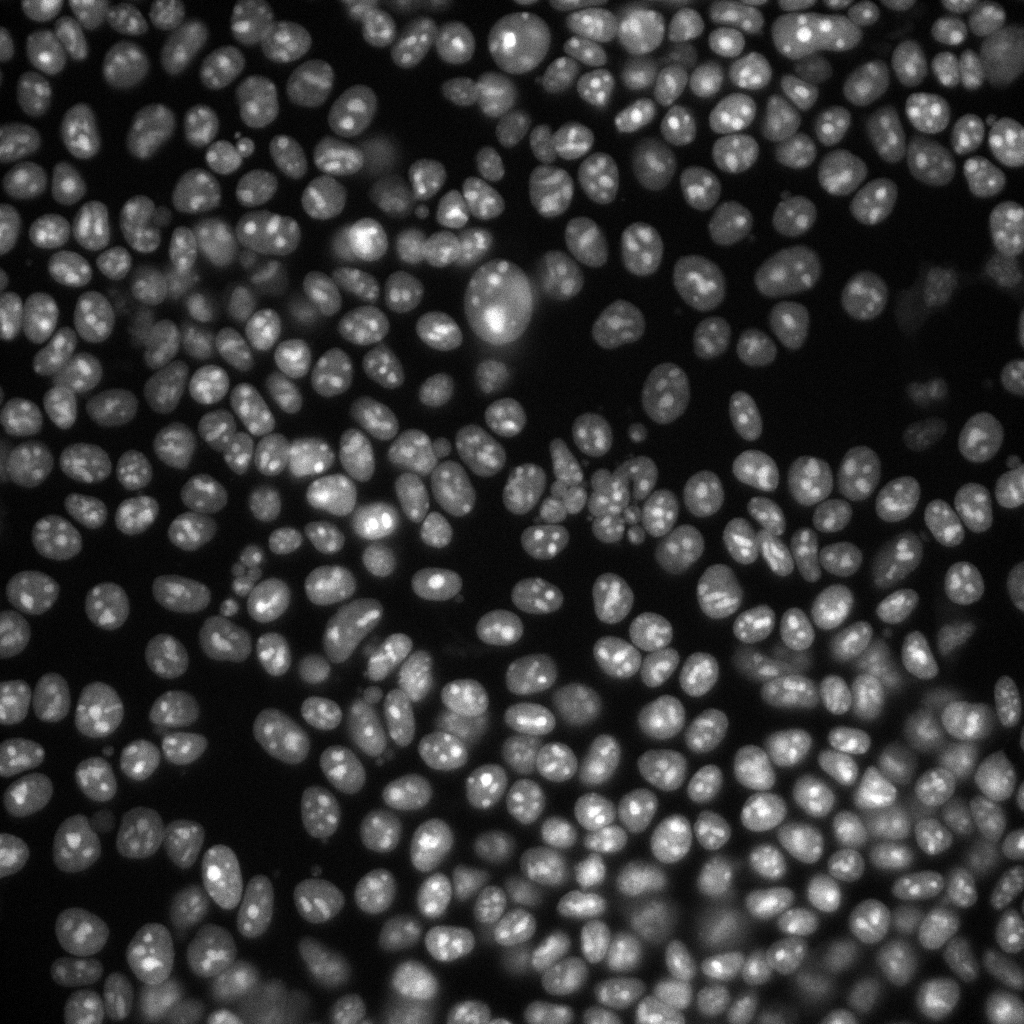

Supplement: Supplementary file 3 — Source Data [file 41467_2020_20757_MOESM3_ESM.zip › source_data/figure 1b-c images/HP1a_woDox_images/Position029_t454_ch02.tif]

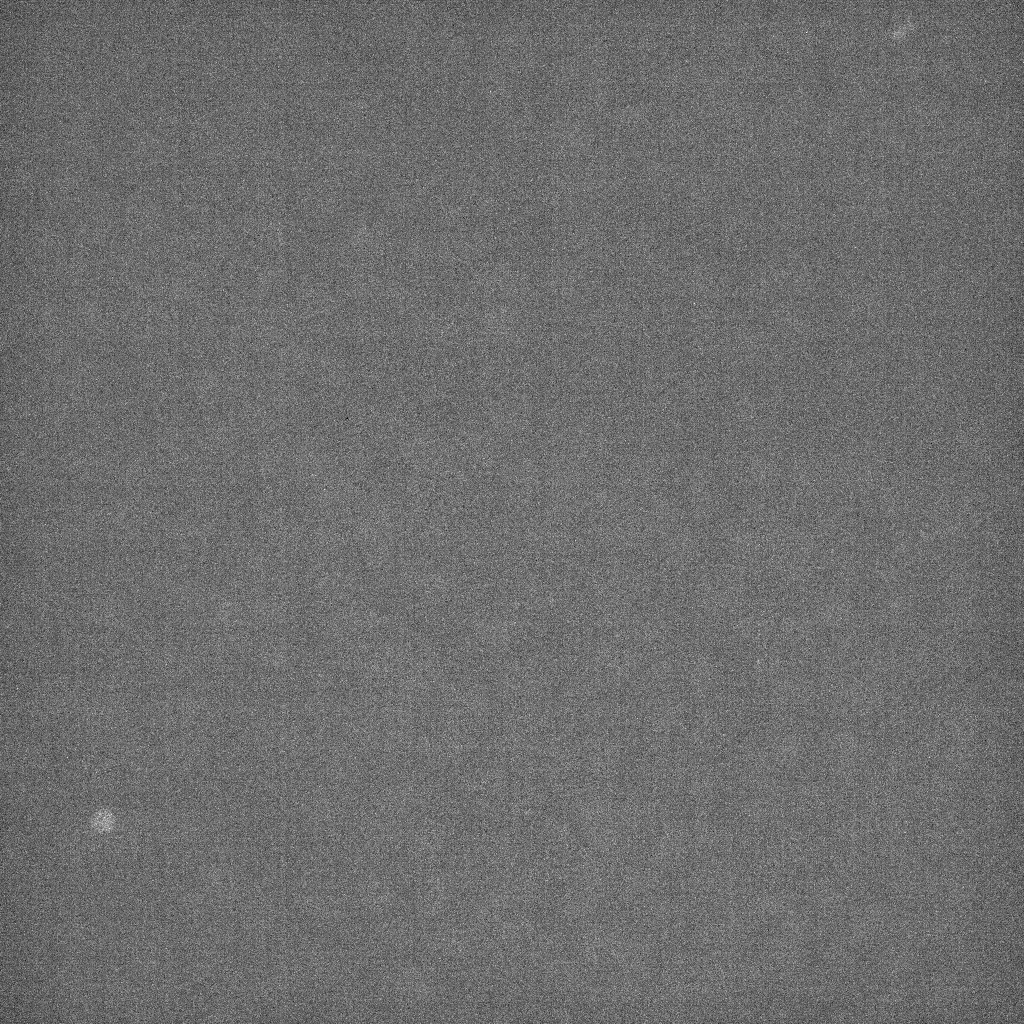

Supplement: Supplementary file 3 — Source Data [file 41467_2020_20757_MOESM3_ESM.zip › source_data/figure 1b-c images/HP1a_woDox_images/Position029_t454_ch03.tif]
